# Supplementary material for: Improved gene therapy for spinal muscular atrophy in mice using codon-optimized hSMN1 transgene and hSMN1 gene-derived promotor
Source: EMBO Mol Med. 2024 Feb 27;16(4):20. doi: 10.1038/s44321-024-00037-x (PMC11018631; doi:10.1038/s44321-024-00037-x)
Supplement: Supplementary file 1 — Appendix [file 44321_2024_37_MOESM1_ESM.pdf]

Appendix Table of Contents

1. Appendix Fig S1
2. Appendix Fig S2

2346 bases
2 features

```
OV    1   CTGCGCGCTCGCTCGCTCACTGAGGCCGCCCGGGCAAAGCCCGGGCGTCGGGCGACCTTTGGTCGCCCGG   70
BMK   1   CTGCGCGCTCGCTCGCTCACTGAGGCCGCCCGGGCAAAGCCCGGGCGTCGGGCGACCTTTGGTCGCCCGG   70
```

2372 bases
10 features

mutant ITR

2346 bases
2 features

```
OV    71  CCTCAGTGAGCGAGCGAGCGCGCAGAGAGGGAGTG-------------------------GAATTCACGC  115
BMK   71  CCTCAGTGAGCGAGCGAGCGCGCAGAGAGGGAGTGTAGCCATGCTCTAGGAAGATCAATTCAATTCACGC  140
```

2372 bases
10 features

mutant ITR

2346 bases
2 features

```
OV    116 G-TGGATCTGAATTCAATTCACGCGTGGTACCTCTGGTCGTTACATAACTTACGGTAAATGGCCCGCCTG  184
BMK   141 GCCGGATCTGAATTCAATTCACGCGTGGTACCTCTGGTCGTTACATAACTTACGGTAAATGGCCCGCCTG  210
```

2372 bases
10 features

CMV enhancer

2346 bases
2 features

```
OV    185 GCTGACCGCCCAACGACCCCCGCCCATTGACGTCAATAATGACGTATGTTCCCATAGTAACGCCAATAGG  254
BMK   211 GCTGACCGCCCAACGACCCCCGCCCATTGACGTCAATAATGACGTATGTTCCCATAGTAACGCCAATAGG  280
```

2372 bases
10 features

CMV enhancer

2346 bases
2 features

```
OV    255 GACTTTCCATTGACGTCAATGGGTGGAGTATTTACGGTAAACTGCCCACTTGGCAGTACATCAAGTGTAT  324
BMK   281 GACTTTCCATTGACGTCAATGGGTGGAGTATTTACGGTAAACTGCCCACTTGGCAGTACATCAAGTGTAT  350
```

2372 bases
10 features

CMV enhancer

2346 bases
2 features

```
OV    325 CATATGCCAAGTACGCCCCCTATTGACGTCAATGACGGTAAATGGCCCGCCTGGCATTATGCCCAGTACA  394
BMK   351 CATATGCCAAGTACGCCCCCTATTGACGTCAATGACGGTAAATGGCCCGCCTGGCATTATGCCCAGTACA  420
```

2372 bases
10 features

CMV enhancer

2346 bases
2 features

```
OV    395 TGACCTTATGGGACTTTCCTACTTGGCAGTACATCTACTCGAGGCCACGTTCTGCTTCACTCTCCCCATC  464
BMK   421 TGACCTTATGGGACTTTCCTACTTGGCAGTACATCTACTCGAGGCCACGTTCTGCTTCACTCTCCCCATC  490
```

2372 bases
10 features

CMV enhancer   Chicken-β- actin core promoter

```
2346 bases
2 features
OV   465  TCCCCCCCCTCCCCACCCCCAATTTTGTATTTATTTATTTTTTAATTATTTTGTGCAGCGATGGGGGCGG  534
          ||||||||||||||||||||||||||||||||||||||||||||||||||||||||||||||||||||||
BMK  491  TCCCCCCCCTCCCCACCCCCAATTTTGTATTTATTTATTTTTTAATTATTTTGTGCAGCGATGGGGGCGG  560
2372 bases
10 features
          Chicken-β- actin core promoter
```

```
2346 bases
2 features
OV   535  GGGGGGGGGGGGGGCGCGCGCCAGGCGGGGCGGGGCGGGGCGAGGGGCGGGGCGGGGCGAGGCGGAGAGG  604
          ||||||||||||||||||||||||||||||||||||||||||||||||||||||||||||||||||||||
BMK  561  GGGGGGGGGGGGGGCGCGCGCCAGGCGGGGCGGGGCGGGGCGAGGGGCGGGGCGGGGCGAGGCGGAGAGG  630
2372 bases
10 features
          Chicken-β- actin core promoter
```

```
2346 bases
2 features
OV   605  TGCGGCGGCAGCCAATCAGAGCGGCGCGCTCCGAAAGTTTCCTTTTATGGCGAGGCGGCGGCGGCGGCGG  674
          ||||||||||||||||||||||||||||||||||||||||||||||||||||||||||||||||||||||
BMK  631  TGCGGCGGCAGCCAATCAGAGCGGCGCGCTCCGAAAGTTTCCTTTTATGGCGAGGCGGCGGCGGCGGCGG  700
2372 bases
10 features
          Chicken-β- actin core promoter
```

```
2346 bases
2 features
OV   675  CCCTATAAAAAGCGAAGCGCGCGGCGGGCGGGAGCGGGATCAGCCACCGCGGTGGCGGCCTAGAGTCGAC  744
          ||||||||||||||||||||||||||||||||||||||||||||||||||||||||||||||||||||||
BMK  701  CCCTATAAAAAGCGAAGCGCGCGGCGGGCGGGAGCGGGATCAGCCACCGCGGTGGCGGCCTAGAGTCGAC  770
2372 bases
10 features
          Chicken-β- actin core promoter
```

```
2346 bases
2 features
          SV40 intron
OV   745  GAGGAACTGAAAAACCAGAAAGTTAACTGGTAAGTTTAGTCTTTTTGTCTTTTATTTCAGGTCCCGGATC  814
          ||||||||||||||||||||||||||||||||||||||||||||||||||||||||||||||||||||||
BMK  771  GAGGAACTGAAAAACCAGAAAGTTAACTGGTAAGTTTAGTCTTTTTGTCTTTTATTTCAGGTCCCGGATC  840
2372 bases
10 features
          SV40 intron
```

```
2346 bases
2 features
          SV40 intron
OV   815  CGGTGGTGGTGCAAATCAAAGAACTGCTCCTCAGTGGATGTTGCCTTTACTTCTAGGCCTGTACGGAAGT  884
          ||||||||||||||||||||||||||||||||||||||||||||||||||||||||||||||||||||||
BMK  841  CGGTGGTGGTGCAAATCAAAGAACTGCTCCTCAGTGGATGTTGCCTTTACTTCTAGGCCTGTACGGAAGT  910
2372 bases
10 features
          SV40 intron
```

```
2346 bases
2 features
OV   885  GTTACTTCTGCTCTAAAAGCTGCGGAATTGTACCCGCGGCCGATCCACCGGTCCGGAATTCCCGGGATAT  954
          ||||||||||||||||||||||||||||||||||||||||||||||||||||||||||||||||||||||
BMK  911  GTTACTTCTGCTCTAAAAGCTGCGGAATTGTACCCGCGGCCGATCCACCGGTCCGGAATTCCCGGGATAT  980
2372 bases
10 features
```

2346 bases
2 features

960 970 980 990 1000 1010 1020

OV 955 CGTCGACCCACGCGTCCGGGCCCCACGCTGCGCACCCGCGGGTTTGCTATGGCGATGAGCAGCGGCGGCA 1024

BMK 981 CGTCGACCCACGCGTCCGGGCCCCACGCTGCGCACCCGCGGGTTTGCTATGGCGATGAGCAGCGGCGGCA 1050

990 1000 1010 1020 1030 1040 1050

2372 bases
10 features

1 5
Met Ala Met Ser Ser Gly Gly
SMN cDNA

2346 bases
2 features

1030 1040 1050 1060 1070 1080 1090

OV 1025 GTGGTGGCGGCGTCCCGGAGCAGGAGGATTCCGTGCTGTTCCGGCGCGGCACAGGCCAGAGCGATGATTC 1094

BMK 1051 GTGGTGGCGGCGTCCCGGAGCAGGAGGATTCCGTGCTGTTCCGGCGCGGCACAGGCCAGAGCGATGATTC 1120

1060 1070 1080 1090 1100 1110 1120

2372 bases
10 features

10 15 20 25 30
Ser Gly Gly Gly Val Pro Glu Gln Glu Asp Ser Val Leu Phe Arg Arg Gly Thr Gly Gln Ser Asp Asp Ser
SMN cDNA

2346 bases
2 features

1100 1110 1120 1130 1140 1150 1160

OV 1095 TGACATTTGGGATGATACAGCACTGATAAAAGCATATGATAAAGCTGTGGCTTCATTTAAGCATGCTCTA 1164

BMK 1121 TGACATTTGGGATGATACAGCACTGATAAAAGCATATGATAAAGCTGTGGCTTCATTTAAGCATGCTCTA 1190

1130 1140 1150 1160 1170 1180 1190

2372 bases
10 features

35 40 45 50
Asp Ile Trp Asp Asp Thr Ala Leu Ile Lys Ala Tyr Asp Lys Ala Val Ala Ser Phe Lys His Ala Leu
SMN cDNA

2346 bases
2 features

1170 1180 1190 1200 1210 1220 1230

OV 1165 AAGAATGGTGACATTTGTGAAACTTCGGGTAAACCAAAAACCACACCTAAAAGAAAACCTGCTAAGAAGA 1234

BMK 1191 AAGAATGGTGACATTTGTGAAACTTCGGGTAAACCAAAAACCACACCTAAAAGAAAACCTGCTAAGAAGA 1260

1200 1210 1220 1230 1240 1250 1260

2372 bases
10 features

55 60 65 70 75
Lys Asn Gly Asp Ile Cys Glu Thr Ser Gly Lys Pro Lys Thr Thr Pro Lys Arg Lys Pro Ala Lys Lys
SMN cDNA

2346 bases
2 features

1240 1250 1260 1270 1280 1290 1300

OV 1235 ATAAAAGCCAAAAAGAAGAATACTGCAGCTTCCTTACAACAGTGGAAAGTTGGGGACAAATGTTCTGCCAT 1304

BMK 1261 ATAAAAGCCAAAAAGAAGAATACTGCAGCTTCCTTACAACAGTGGAAAGTTGGGGACAAATGTTCTGCCAT 1330

1270 1280 1290 1300 1310 1320 1330

2372 bases
10 features

80 85 90 95 100
Asn Lys Ser Gln Lys Lys Asn Thr Ala Ala Ser Leu Gln Gln Trp Lys Val Gly Asp Lys Cys Ser Ala Ile
SMN cDNA

2346 bases
2 features

1310 1320 1330 1340 1350 1360 1370

OV 1305 TTGGTCAGAAGACGGTTGCATTTACCCAGCTACCATTGCTTCAATTGATTTTAAGAGAGAAACCTGTGTT 1374

BMK 1331 TTGGTCAGAAGACGGTTGCATTTACCCAGCTACCATTGCTTCAATTGATTTTAAGAGAGAAACCTGTGTT 1400

1340 1350 1360 1370 1380 1390 1400

2372 bases
10 features

105 110 115 120
Trp Ser Glu Asp Gly Cys Ile Tyr Pro Ala Thr Ile Ala Ser Ile Asp Phe Lys Arg Glu Thr Cys Val
SMN cDNA

```
2346 bases
2 features
        1380|     1390|     1400|     1410|     1420|     1430|     1440|
OV   1375 GTGGTTTACACTGGATATGGAAATAGAGAGGAGCAAAATCTGTCCGATCTACTTTCCCCAATCTGTGAAG 1444
          ||||||||||||||||||||||||||||||||||||||||||||||||||||||||||||||||||||||
BMK  1401 GTGGTTTACACTGGATATGGAAATAGAGAGGAGCAAAATCTGTCCGATCTACTTTCCCCAATCTGTGAAG 1470
              1410|     1420|     1430|     1440|     1450|     1460|     1470|
2372 bases
10 features
125 130 135 140 145
Val Val Tyr Thr Gly Tyr Gly Asn Arg Glu Glu Gln Asn Leu Ser Asp Leu Leu Ser Pro Ile Cys Glu
SMN cDNA

2346 bases
2 features
        1450|     1460|     1470|     1480|     1490|     1500|     1510|
OV   1445 TAGCTAATAATATAGAACAGAATGCTCAAGAGAATGAAAATGAAAGCCAAGTTTCAACAGATGAAAGTGA 1514
          ||||||||||||||||||||||||||||||||||||||||||||||||||||||||||||||||||||||
BMK  1471 TAGCTAATAATATAGAACAGAATGCTCAAGAGAATGAAAATGAAAGCCAAGTTTCAACAGATGAAAGTGA 1540
              1480|     1490|     1500|     1510|     1520|     1530|     1540|
2372 bases
10 features
150 155 160 165 170
Val Ala Asn Asn Ile Glu Gln Asn Ala Gln Glu Asn Glu Asn Glu Ser Gln Val Ser Thr Asp Glu Ser Glu
SMN cDNA

2346 bases
2 features
        1520|     1530|     1540|     1550|     1560|     1570|     1580|
OV   1515 GAACTCCAGGTCTCCTGGAAATAAATCAGATAACATCAAGCCCAAATCTGCTCCATGGAACTCTTTTCTC 1584
          ||||||||||||||||||||||||||||||||||||||||||||||||||||||||||||||||||||||
BMK  1541 GAACTCCAGGTCTCCTGGAAATAAATCAGATAACATCAAGCCCAAATCTGCTCCATGGAACTCTTTTCTC 1610
              1550|     1560|     1570|     1580|     1590|     1600|     1610|
2372 bases
10 features
175 180 185 190
Asn Ser Arg Ser Pro Gly Asn Lys Ser Asp Asn Ile Lys Pro Lys Ser Ala Pro Trp Asn Ser Phe Leu
SMN cDNA

2346 bases
2 features
        1590|     1600|     1610|     1620|     1630|     1640|     1650|
OV   1585 CCTCCACCACCCCCCATGCCAGGGCCAAGACTGGGACCAGGAAAGCCAGGTCTAAAATTCAATGGCCCAC 1654
          ||||||||||||||||||||||||||||||||||||||||||||||||||||||||||||||||||||||
BMK  1611 CCTCCACCACCCCCCATGCCAGGGCCAAGACTGGGACCAGGAAAGCCAGGTCTAAAATTCAATGGCCCAC 1680
              1620|     1630|     1640|     1650|     1660|     1670|     1680|
2372 bases
10 features
195 200 205 210 215
Pro Pro Pro Pro Pro Met Pro Gly Pro Arg Leu Gly Pro Gly Lys Pro Gly Leu Lys Phe Asn Gly Pro
SMN cDNA

2346 bases
2 features
        1660|     1670|     1680|     1690|     1700|     1710|     1720|
OV   1655 CACCGCCACCGCCACCACCACCACCCCACTTACTATCATGCTGGCTGCCTCCATTTCCTTCTGGACCACC 1724
          ||||||||||||||||||||||||||||||||||||||||||||||||||||||||||||||||||||||
BMK  1681 CACCGCCACCGCCACCACCACCACCCCACTTACTATCATGCTGGCTGCCTCCATTTCCTTCTGGACCACC 1750
              1690|     1700|     1710|     1720|     1730|     1740|     1750|
2372 bases
10 features
220 225 230 235 240
Pro Pro Pro Pro Pro Pro Pro Pro Pro His Leu Leu Ser Cys Trp Leu Pro Pro Phe Pro Ser Gly Pro Pro
SMN cDNA

2346 bases
2 features
        1730|     1740|     1750|     1760|     1770|     1780|     1790|
OV   1725 AATAATTCCCCCACCACCTCCCATATGTCCAGATTCTCTTGATGATGCTGATGCTTTGGGAAGTATGTTA 1794
          ||||||||||||||||||||||||||||||||||||||||||||||||||||||||||||||||||||||
BMK  1751 AATAATTCCCCCACCACCTCCCATATGTCCAGATTCTCTTGATGATGCTGATGCTTTGGGAAGTATGTTA 1820
              1760|     1770|     1780|     1790|     1800|     1810|     1820|
2372 bases
10 features
245 250 255 260
Ile Ile Pro Pro Pro Pro Pro Ile Cys Pro Asp Ser Leu Asp Asp Ala Asp Ala Leu Gly Ser Met Leu
SMN cDNA
```

```
2346 bases
2 features
        1800      1810      1820      1830      1840      1850      1860
OV   1795 ATTTCATGGTACATGAGTGGCTATCATACTGGCTATTATATGGGTTTTAGACAAAATCAAAAAGAAGGAA 1864
          ||||||||||||||||||||||||||||||||||||||||||||||||||||||||||||||||||||||
BMK  1821 ATTTCATGGTACATGAGTGGCTATCATACTGGCTATTATATGGGTTTTAGACAAAATCAAAAAGAAGGAA 1890
              1830      1840      1850      1860      1870      1880      1890
2372 bases
10 features
     265           270             275             280            285
     Ile Ser Trp Tyr Met Ser Gly Tyr His Thr Gly Tyr Tyr Met Gly Phe Arg Gln Asn Gln Lys Glu Gly
     SMN cDNA

2346 bases
2 features
        1870      1880      1890      1900      1910      1920      1930
OV   1865 GGTGCTCACATTCCTTAAATTAAGGAGAAATGCTGGCATAGAGCAGCACTAAATGACACCACTAAAGAAA 1934
          ||||||||||||||||||||||||||||||||||||||||||||||||||||||||||||||||||||||
BMK  1891 GGTGCTCACATTCCTTAAATTAAGGAGAAATGCTGGCATAGAGCAGCACTAAATGACACCACTAAAGAAA 1960
              1900      1910      1920      1930      1940      1950      1960
2372 bases
10 features
             290               295
     Arg Cys Ser His Ser Leu Asn *
     SMN cDNA

                                                          bGH poly(A) signal
2346 bases
2 features
        1940      1950      1960      1970      1980      1990      2000
OV   1935 CGATCAGACAGATCTAGAAAGCTTATCGATACCGTCGACTAGAGCTCGCTGATCAGCCTCGACTGTGCCT 2004
          ||||||||||||||||||||||||||||||||||||||||||||||||||||||||||||||||||||||
BMK  1961 CGATCAGACAGATCTAGAAAGCTTATCGATACCGTCGACTAGAGCTCGCTGATCAGCCTCGACTGTGCCT 2030
              1970      1980      1990      2000      2010      2020      2030
2372 bases
10 features
                                          bGH poly(A) signal

2346 bases
2 features
                              bGH poly(A) signal
        2010      2020      2030      2040      2050      2060      2070
OV   2005 TCTAGTTGCCAGCCATCTGTTGTTTGCCCCTCCCCCGTGCCTTCCTTGACCCTGGAAGGTGCCACTCCCA 2074
          ||||||||||||||||||||||||||||||||||||||||||||||||||||||||||||||||||||||
BMK  2031 TCTAGTTGCCAGCCATCTGTTGTTTGCCCCTCCCCCGTGCCTTCCTTGACCCTGGAAGGTGCCACTCCCA 2100
              2040      2050      2060      2070      2080      2090      2100
2372 bases
10 features
                              bGH poly(A) signal

2346 bases
2 features
                              bGH poly(A) signal
        2080      2090      2100      2110      2120      2130      2140
OV   2075 CTGTCCTTTCCTAATAAAATGAGGAAATTGCATCGCATTGTCTGAGTAGGTGTCATTCTATTCTGGGGGG 2144
          ||||||||||||||||||||||||||||||||||||||||||||||||||||||||||||||||||||||
BMK  2101 CTGTCCTTTCCTAATAAAATGAGGAAATTGCATCGCATTGTCTGAGTAGGTGTCATTCTATTCTGGGGGG 2170
              2110      2120      2130      2140      2150      2160      2170
2372 bases
10 features
                              bGH poly(A) signal

2346 bases
2 features
                          bGH poly(A) signal
        2150      2160      2170      2180      2190      2200      2210
OV   2145 TGGGGTGGGGCAGGACAGCAAGGGGGAGGATTGGGAAGACAATAGCAGGCATGCTGGGGAGAGATCGATC 2214
          ||||||||||||||||||||||||||||||||||||||||||||||||||||||||||||||||||||||
BMK  2171 TGGGGTGGGGCAGGACAGCAAGGGGGAGGATTGGGAAGACAATAGCAGGCATGCTGGGGAGAGATCGATC 2240
              2180      2190      2200      2210      2220      2230      2240
2372 bases
10 features
                          bGH poly(A) signal
```

```
2346 bases
2 features

OV    2215  TGAGGAACCCCTAGTGATGGAGTTGGCCACTCCCTCTCTGCGCGCTCGCTCGCTCACTGAGGCCGGGCGA  2284
            ||||||||||||||||||||||||||||||||||||||||||||||||||||||||||||||||||||||
BMK   2241  TGAGGAACCCCTAGTGATGGAGTTGGCCACTCCCTCTCTGCGCGCTCGCTCGCTCACTGAGGCCGGGCGA  2310

2372 bases
10 features
WT ITR

2346 bases
2 features

OV    2285  CCAAAGGTCGCCCGACGCCCGGGCTTTGCCCGGGCGGCCTCAGTGAGCGAGCGAGCGCGCAG  2346
            ||||||||||||||||||||||||||||||||||||||||||||||||||||||||||||||
BMK   2311  CCAAAGGTCGCCCGACGCCCGGGCTTTGCCCGGGCGGCCTCAGTGAGCGAGCGAGCGCGCAG  2372

2372 bases
10 features
WT ITR
```

Appendix Figure S1. Vector genome alignment between the benchmark vector and Zolgensma®. Unaligned sequences are indicated by the red box.

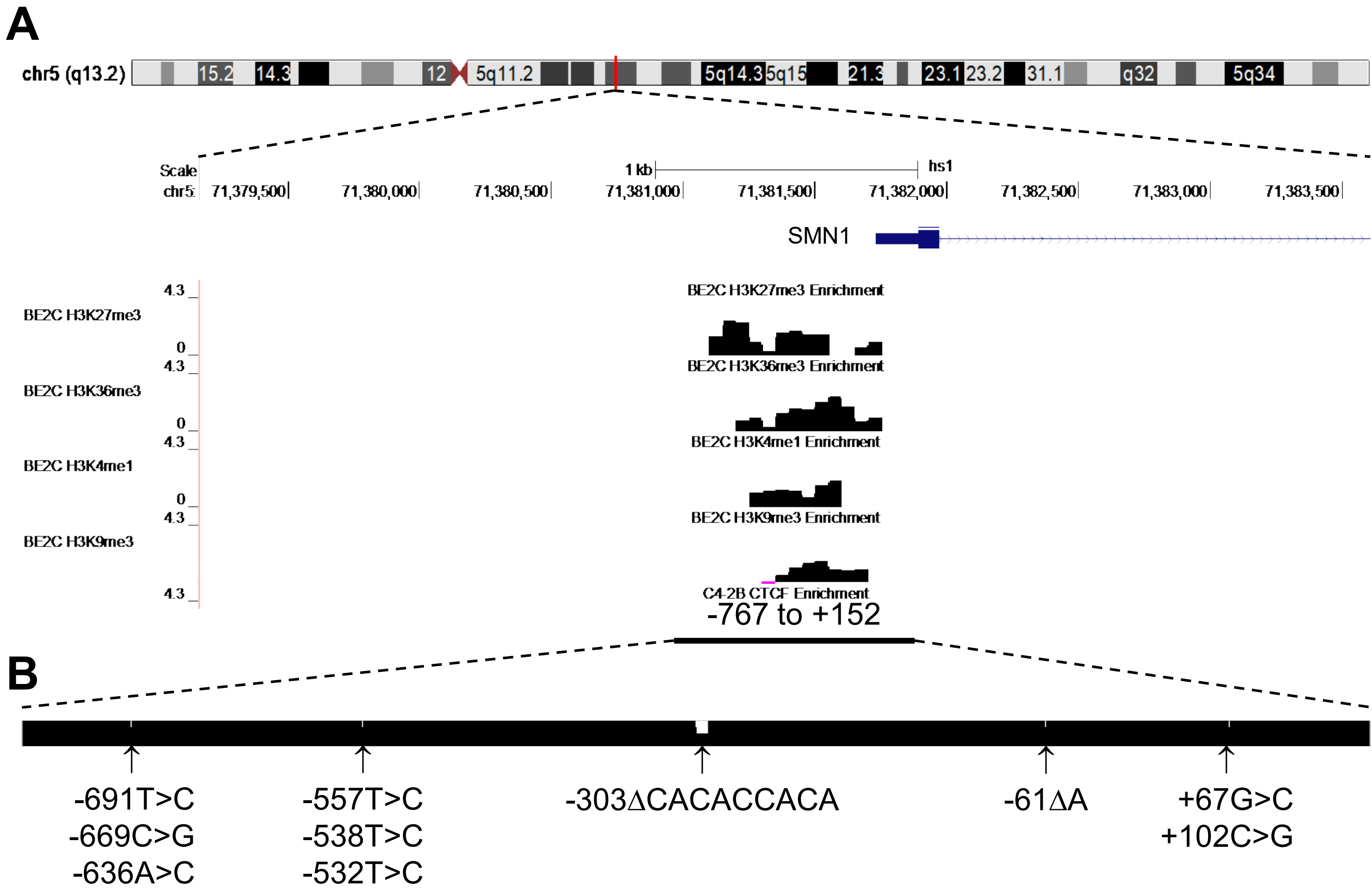

Appendix Figure S2. The SMN promoter (-767 to +152) encompasses enrichment of all epigenetic marks within 2 kb of the transcriptional start site. (A) UCSC genome browser view of epigenetic marks (H3K27me3, H3K36me3, H3K4me1, and H3K9me3) as reported by T2T Encode ENCODE Reanalyses (PMID: 35357915). The four tracks shown for BE2C (neuroblast), are from the most relevant cell line in the T2T CHM13v2.0/hs1 build. **(B)** The SMN promoter, as described in Echaniz-Laguna *et al.*, and used in our 2nd-generation vector design, has ten differences from the T2T build. Each position reported here is in reference to the TSS (+1) reported in Echaniz-Laguna *et al.* For example, "-691T>C" means that the base at position -691 was reported as a thymine (T) in Echaniz-Laguna *et al.* and is annotated as a cytosine (C) in the T2T build; at position -303, there is a 9-nt deletion of CACACCACA
