# Supplementary figures and images for: Improved gene therapy for spinal muscular atrophy in mice using codon-optimized hSMN1 transgene and hSMN1 gene-derived promotor (part 1 of 2)
Source: EMBO Mol Med. 2024 Feb 27;16(4):20. doi: 10.1038/s44321-024-00037-x (PMC11018631; doi:10.1038/s44321-024-00037-x)

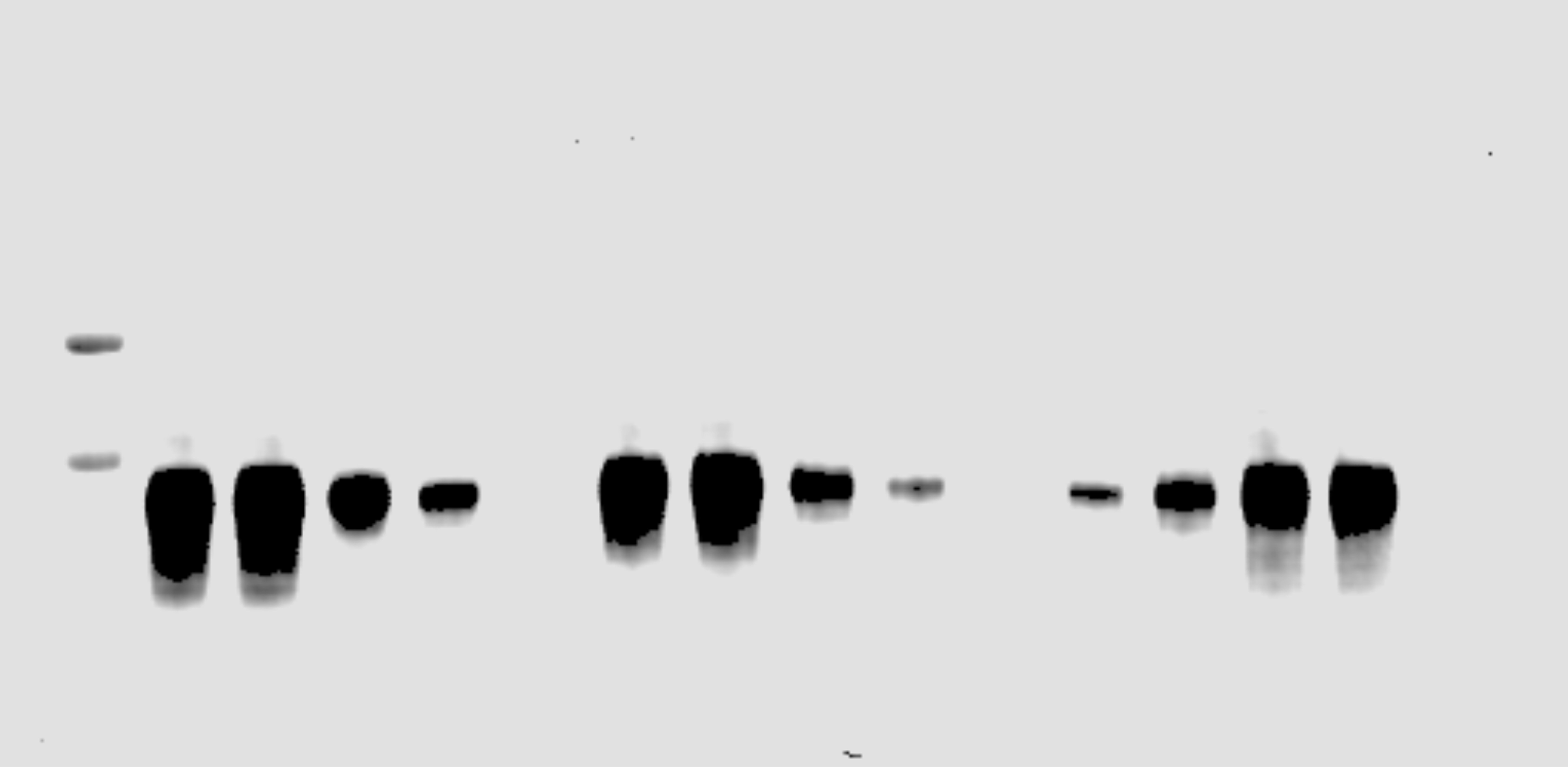

Supplement: Supplementary file 4 — Source Data Fig. 1 [file 44321_2024_37_MOESM4_ESM.zip › Fig 1/Fig1b/Fig. 1b SMN.tif]

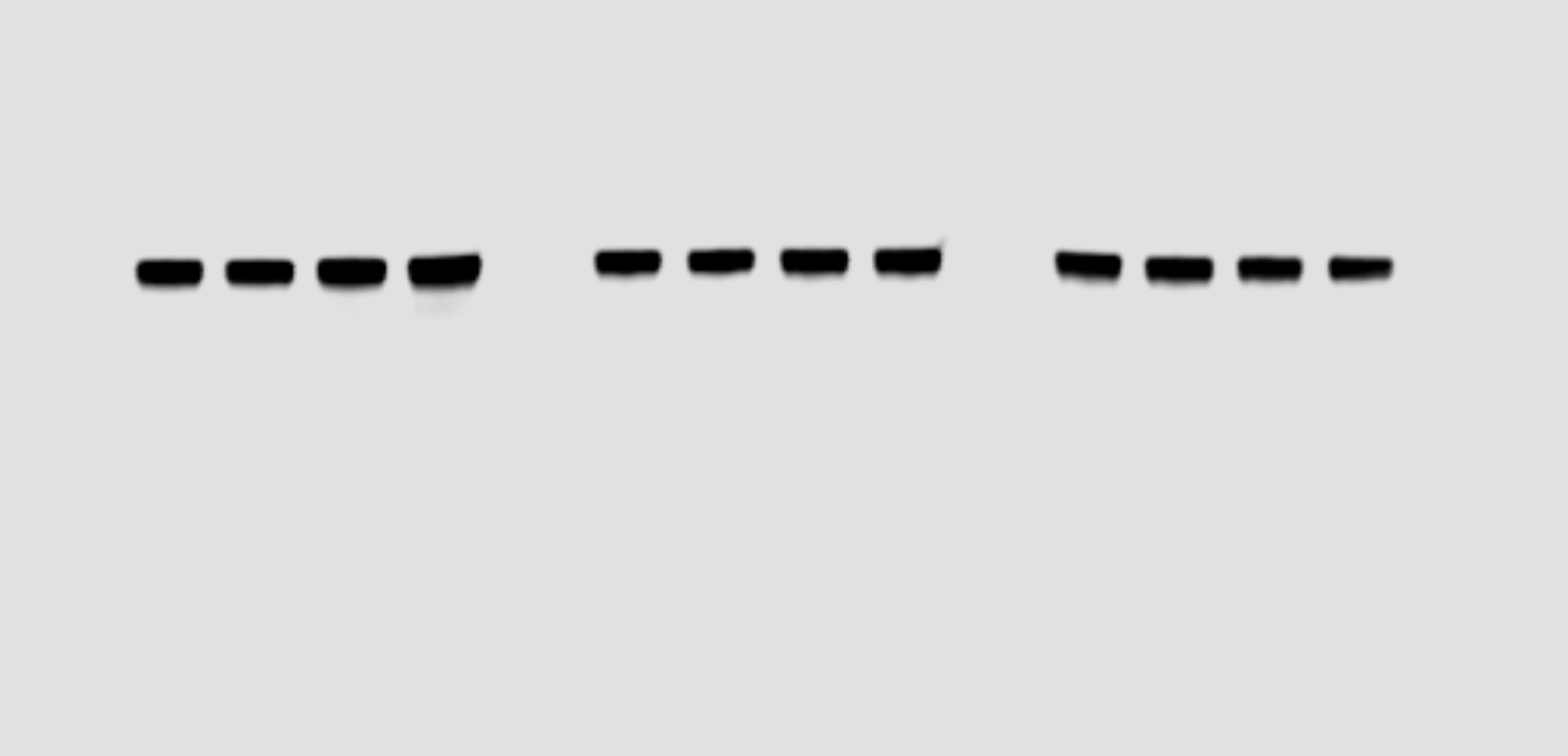

Supplement: Supplementary file 4 — Source Data Fig. 1 [file 44321_2024_37_MOESM4_ESM.zip › Fig 1/Fig1b/Fig. 1b tubulin.tif]

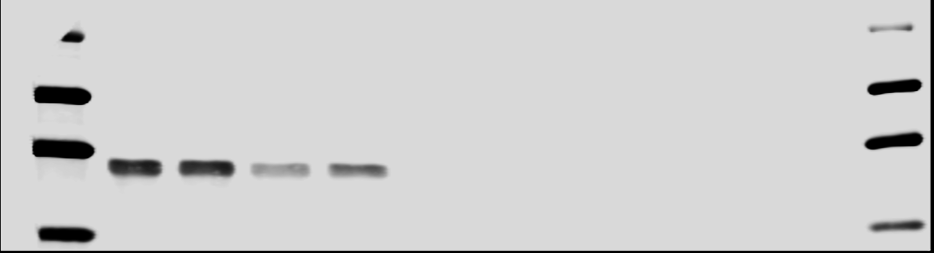

Supplement: Supplementary file 4 — Source Data Fig. 1 [file 44321_2024_37_MOESM4_ESM.zip › Fig 1/Fig1d/Figure 1d SMN low exposure.tif]

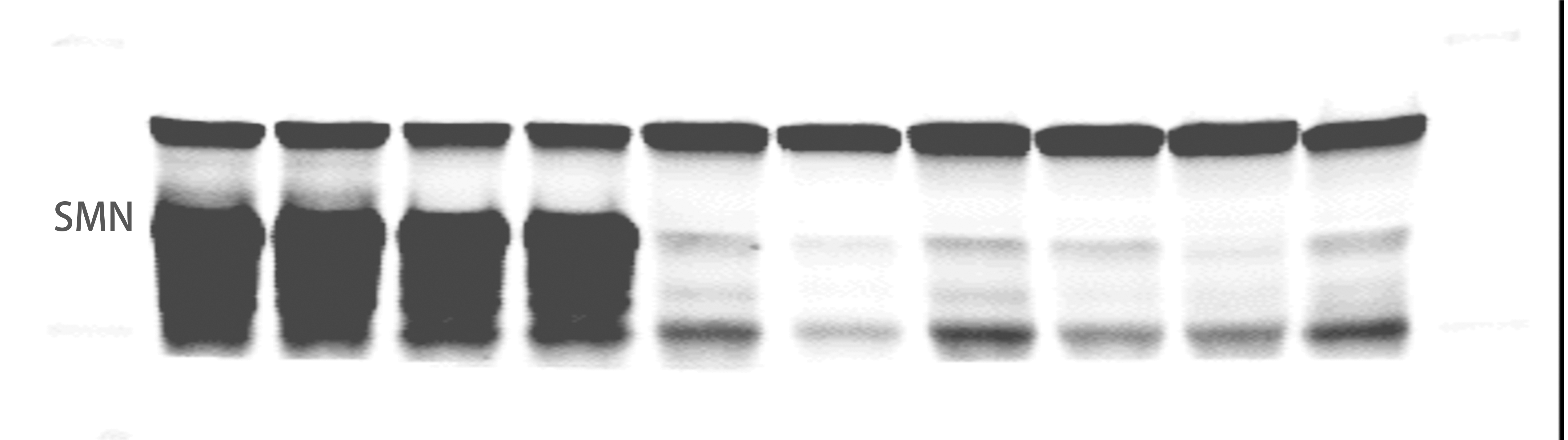

Supplement: Supplementary file 4 — Source Data Fig. 1 [file 44321_2024_37_MOESM4_ESM.zip › Fig 1/Fig1d/Figure 1d SMN.tif]

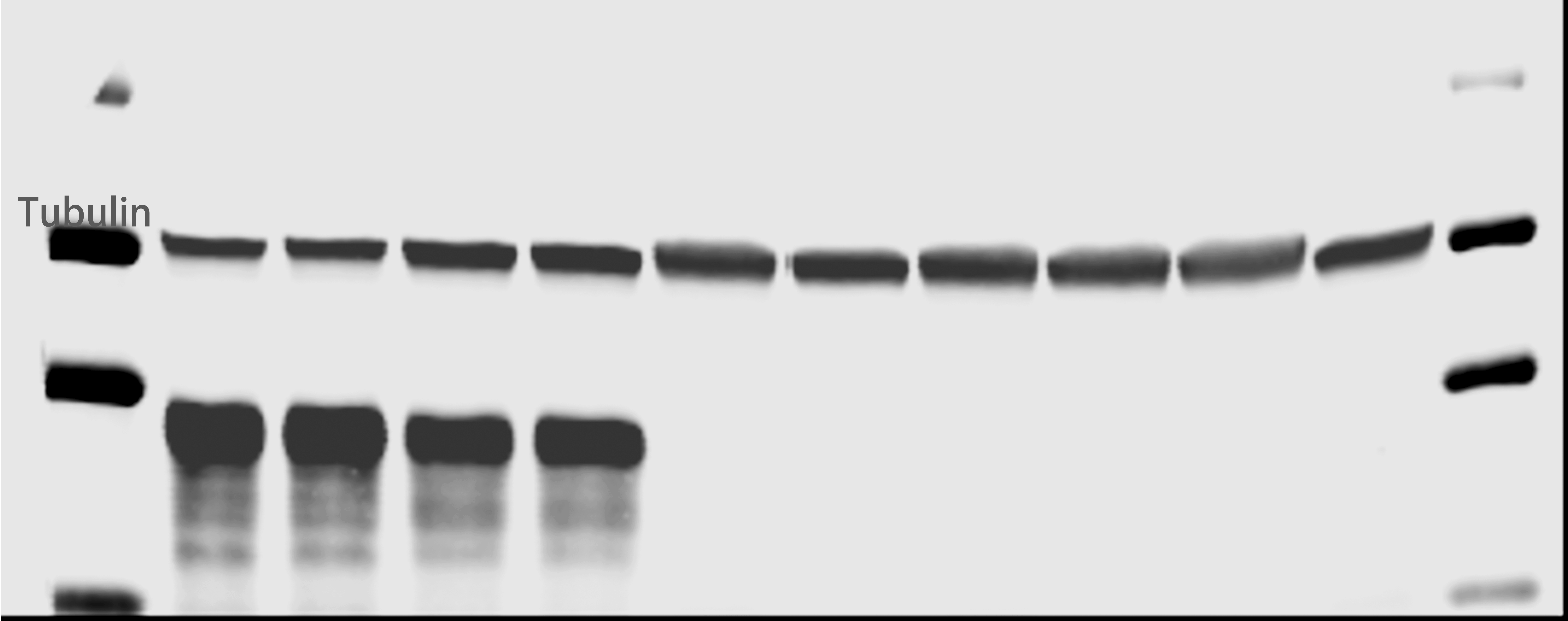

Supplement: Supplementary file 4 — Source Data Fig. 1 [file 44321_2024_37_MOESM4_ESM.zip › Fig 1/Fig1d/Figure 1d Tubulin.tif]

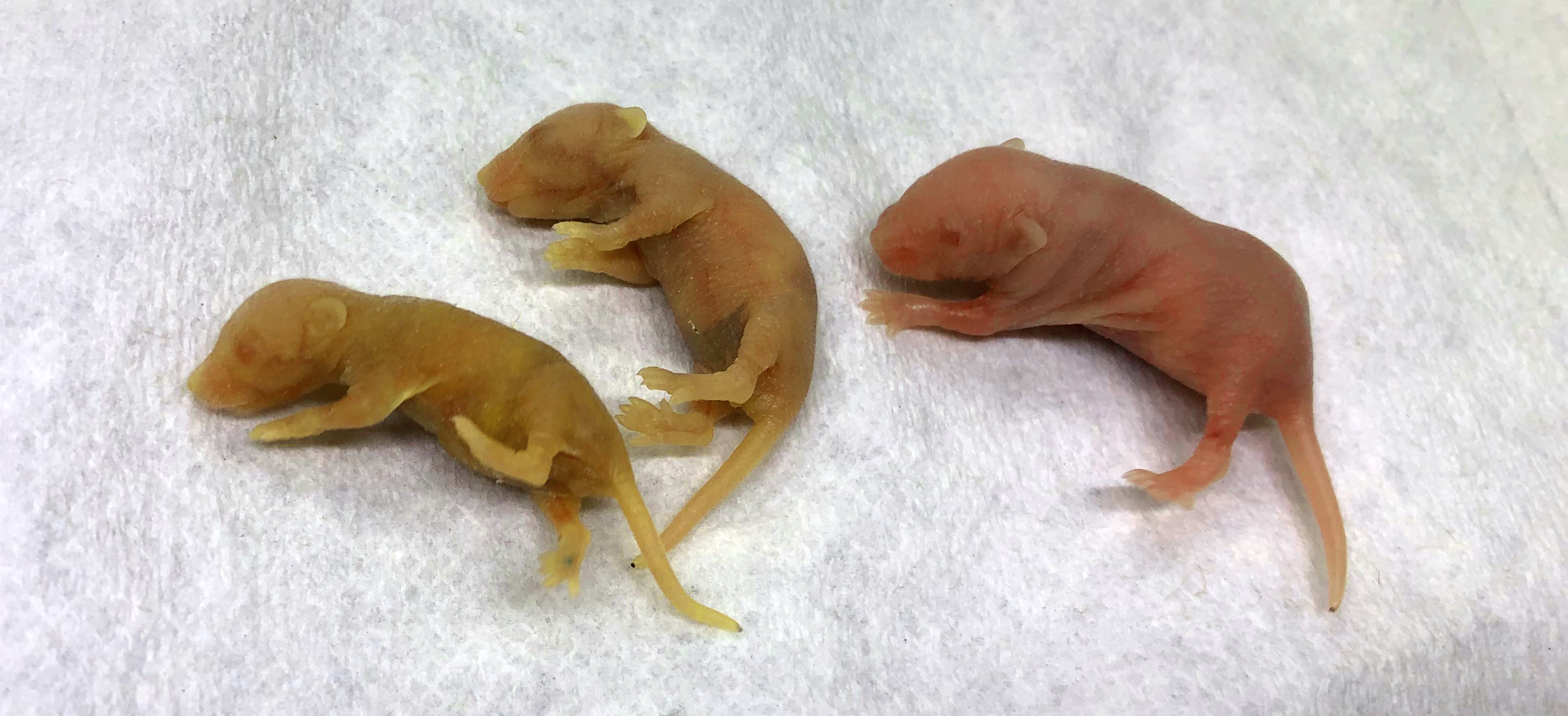

Supplement: Supplementary file 4 — Source Data Fig. 1 [file 44321_2024_37_MOESM4_ESM.zip › Fig 1/Fig1e/Yellow skin_original3.tif]

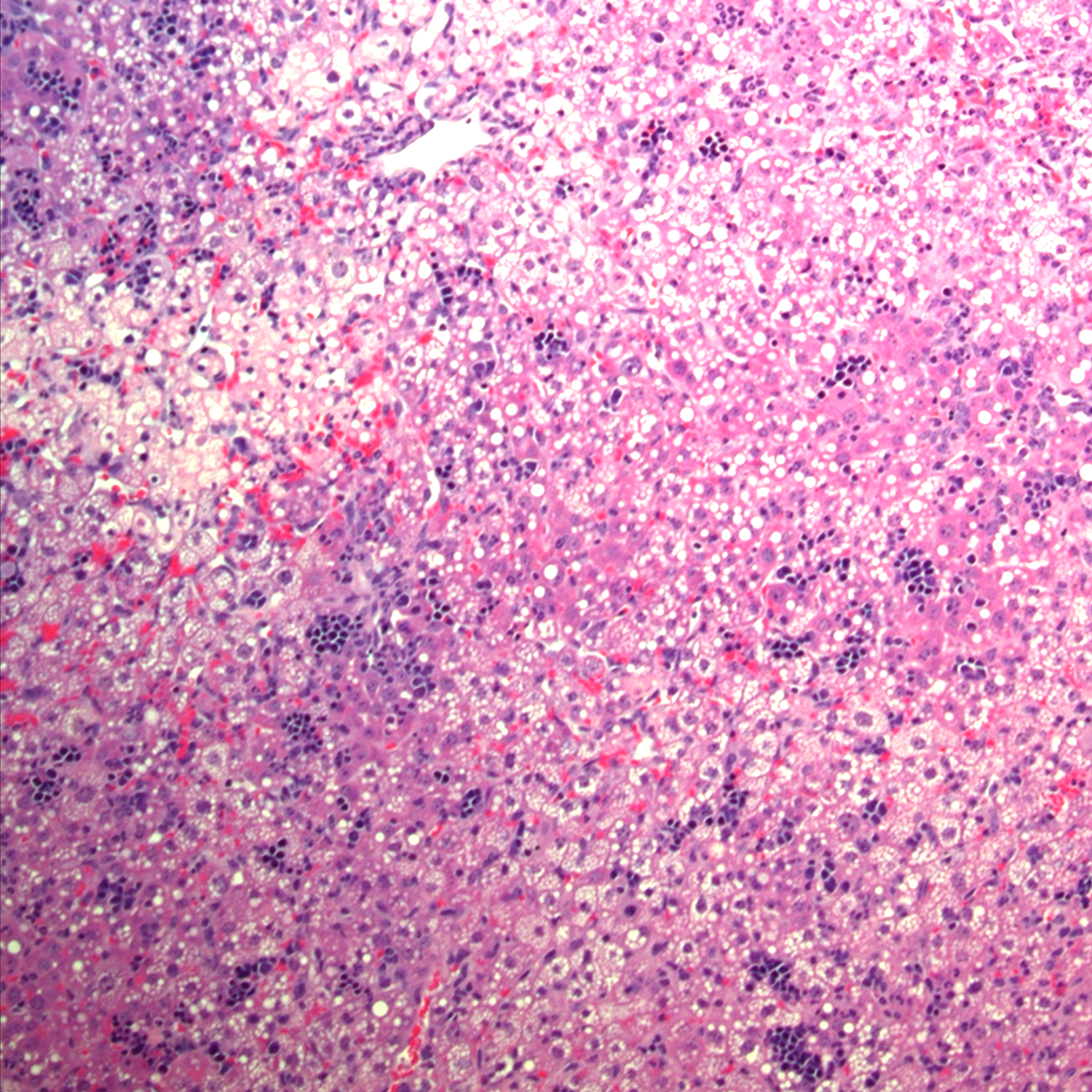

Supplement: Supplementary file 4 — Source Data Fig. 1 [file 44321_2024_37_MOESM4_ESM.zip › Fig 1/Fig1f/Figure 1C_Liver pathology_MD2.tif]

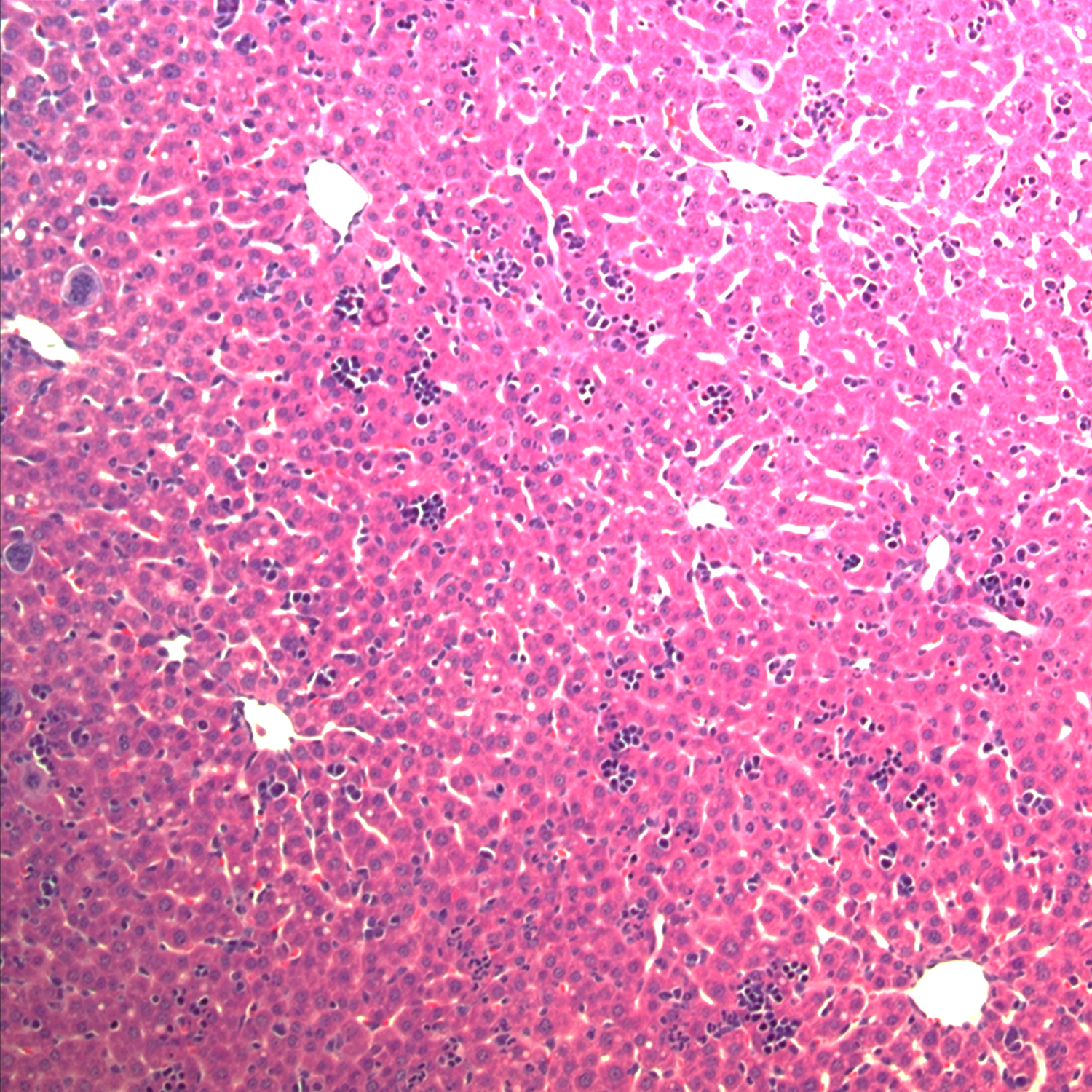

Supplement: Supplementary file 4 — Source Data Fig. 1 [file 44321_2024_37_MOESM4_ESM.zip › Fig 1/Fig1f/Figure 1C_Normal liver_MD2.tif]

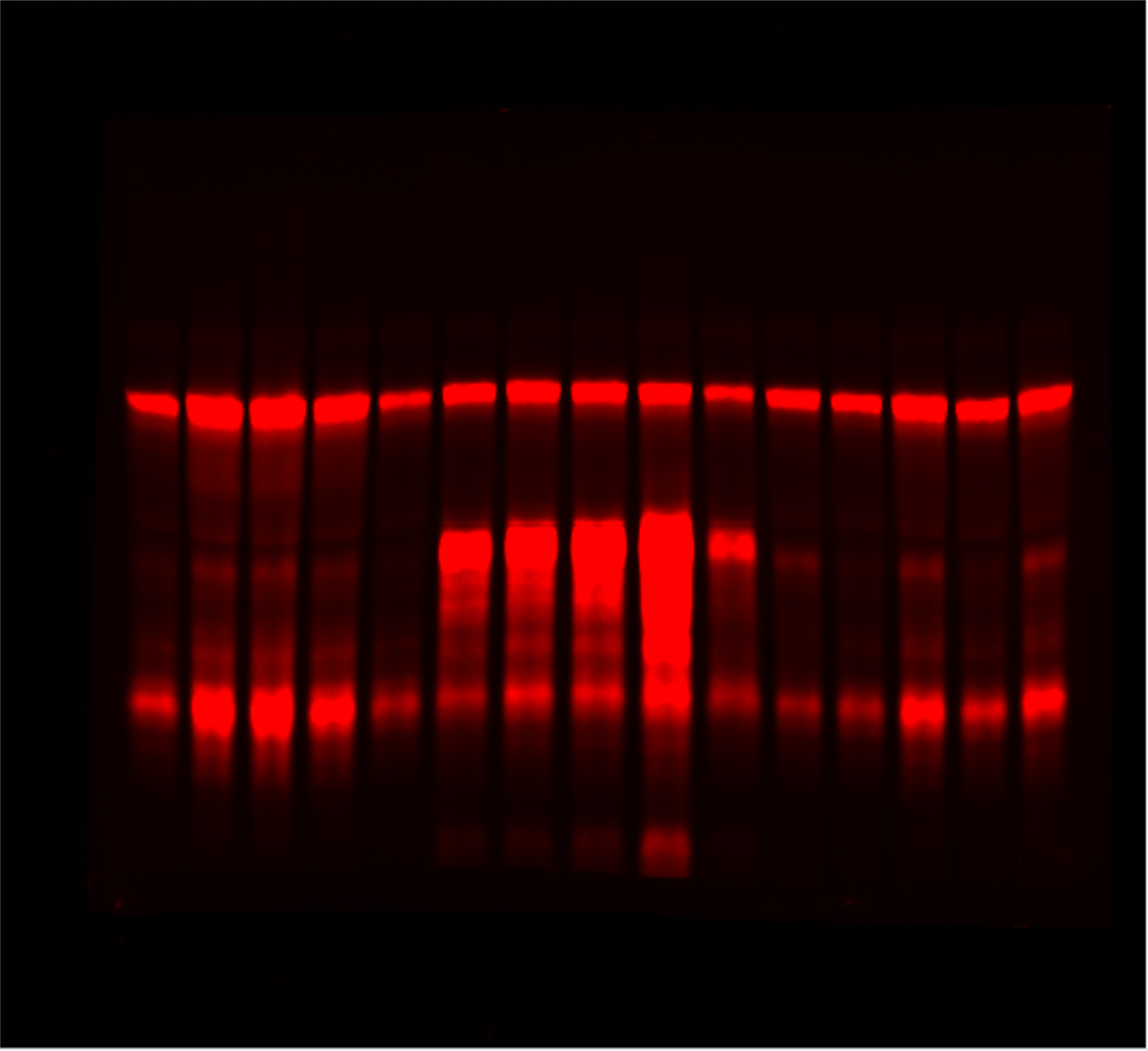

Supplement: Supplementary file 4 — Source Data Fig. 1 [file 44321_2024_37_MOESM4_ESM.zip › Fig 1/Fig1h/Figure 1H_2 Original.tif]

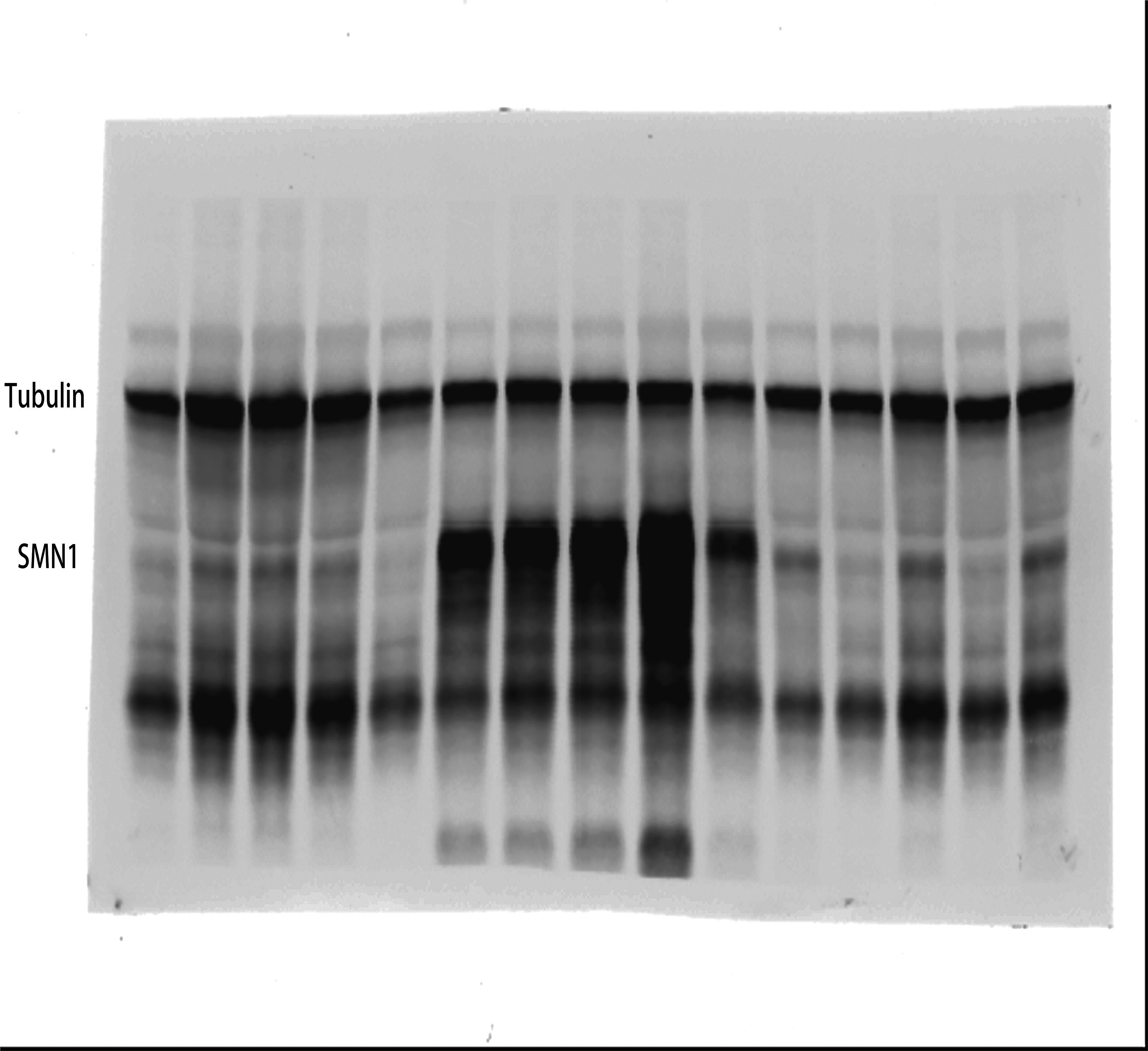

Supplement: Supplementary file 4 — Source Data Fig. 1 [file 44321_2024_37_MOESM4_ESM.zip › Fig 1/Fig1h/Figure 1H_2.tif]

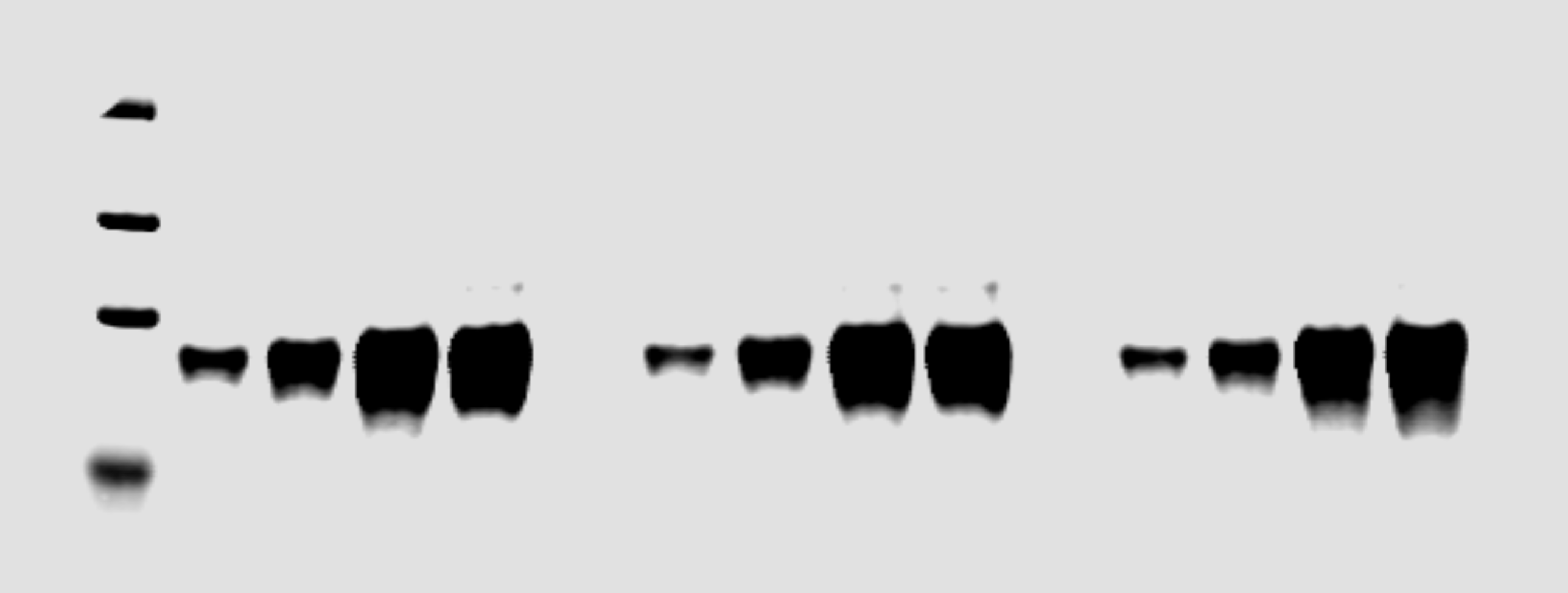

Supplement: Supplementary file 5 — Source Data Fig. 2 [file 44321_2024_37_MOESM5_ESM.zip › Fig 2/Fig2a/figure 2a SMN.tif]

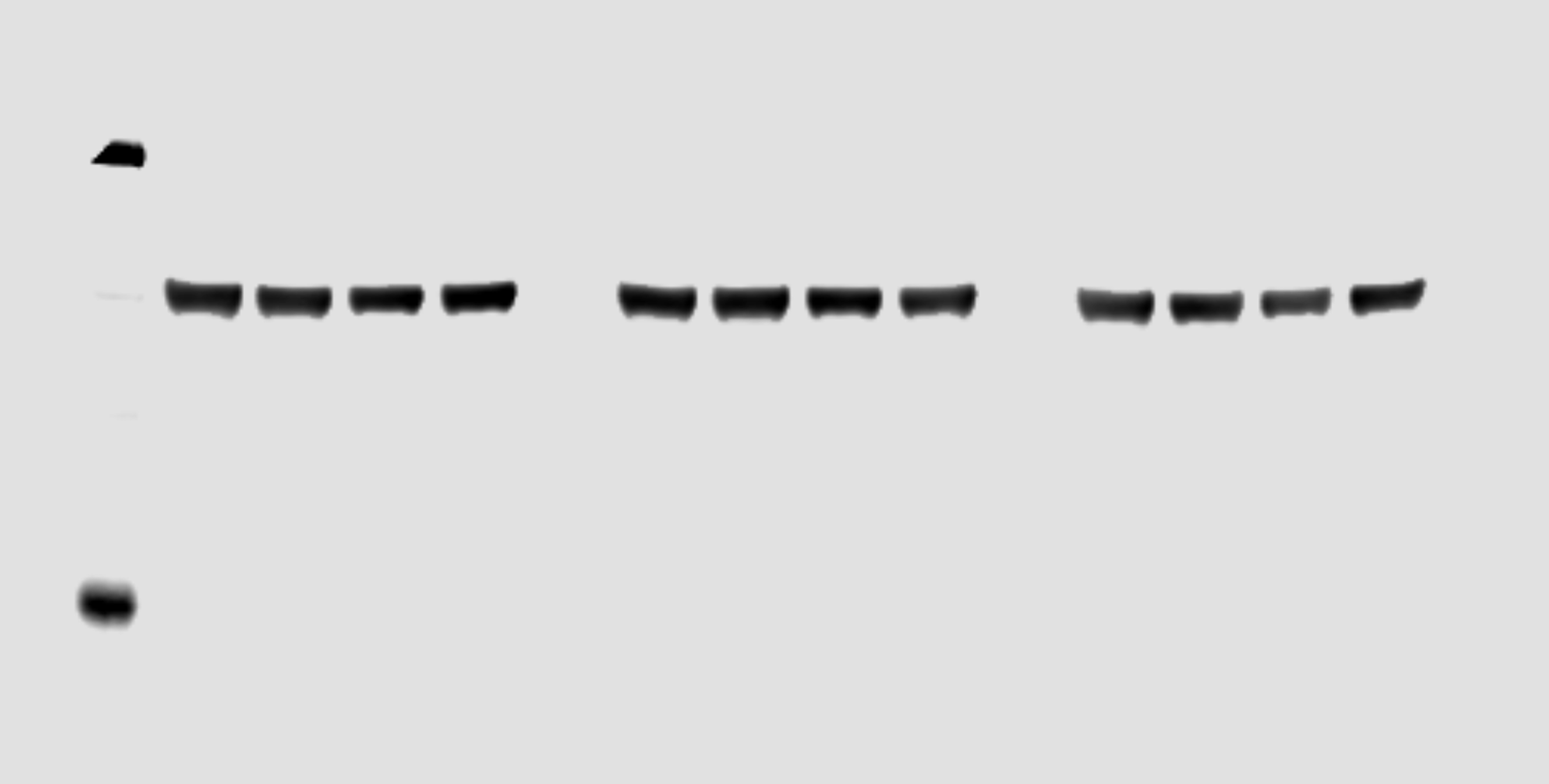

Supplement: Supplementary file 5 — Source Data Fig. 2 [file 44321_2024_37_MOESM5_ESM.zip › Fig 2/Fig2a/figure 2a tubulin.tif]

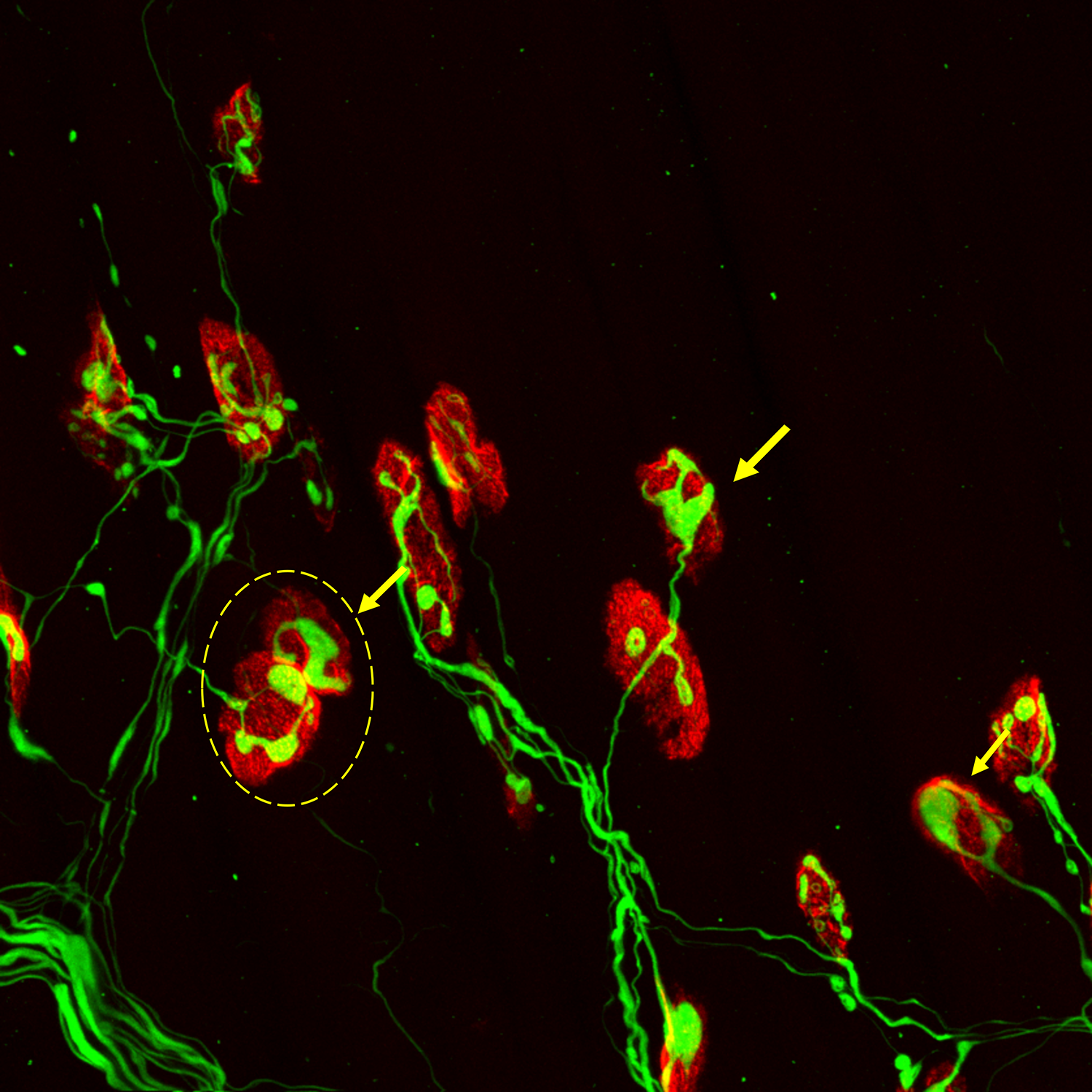

Supplement: Supplementary file 6 — Source Data Fig. 3 [file 44321_2024_37_MOESM6_ESM.zip › Fig 3/Fig3d/Figure 3d_Denervated Large.tif]

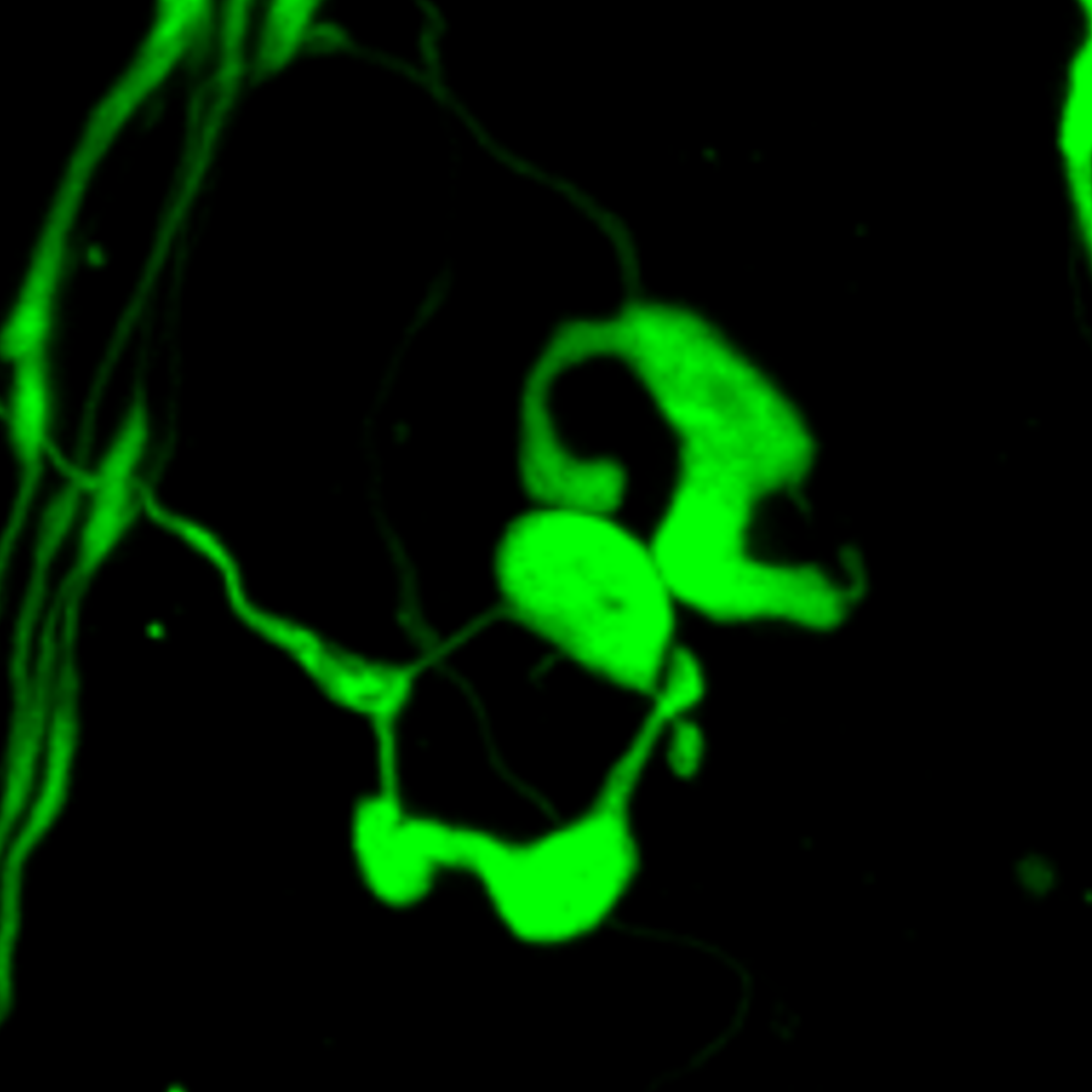

Supplement: Supplementary file 6 — Source Data Fig. 3 [file 44321_2024_37_MOESM6_ESM.zip › Fig 3/Fig3d/Figure 3d_Denervated Small_Green.tif]

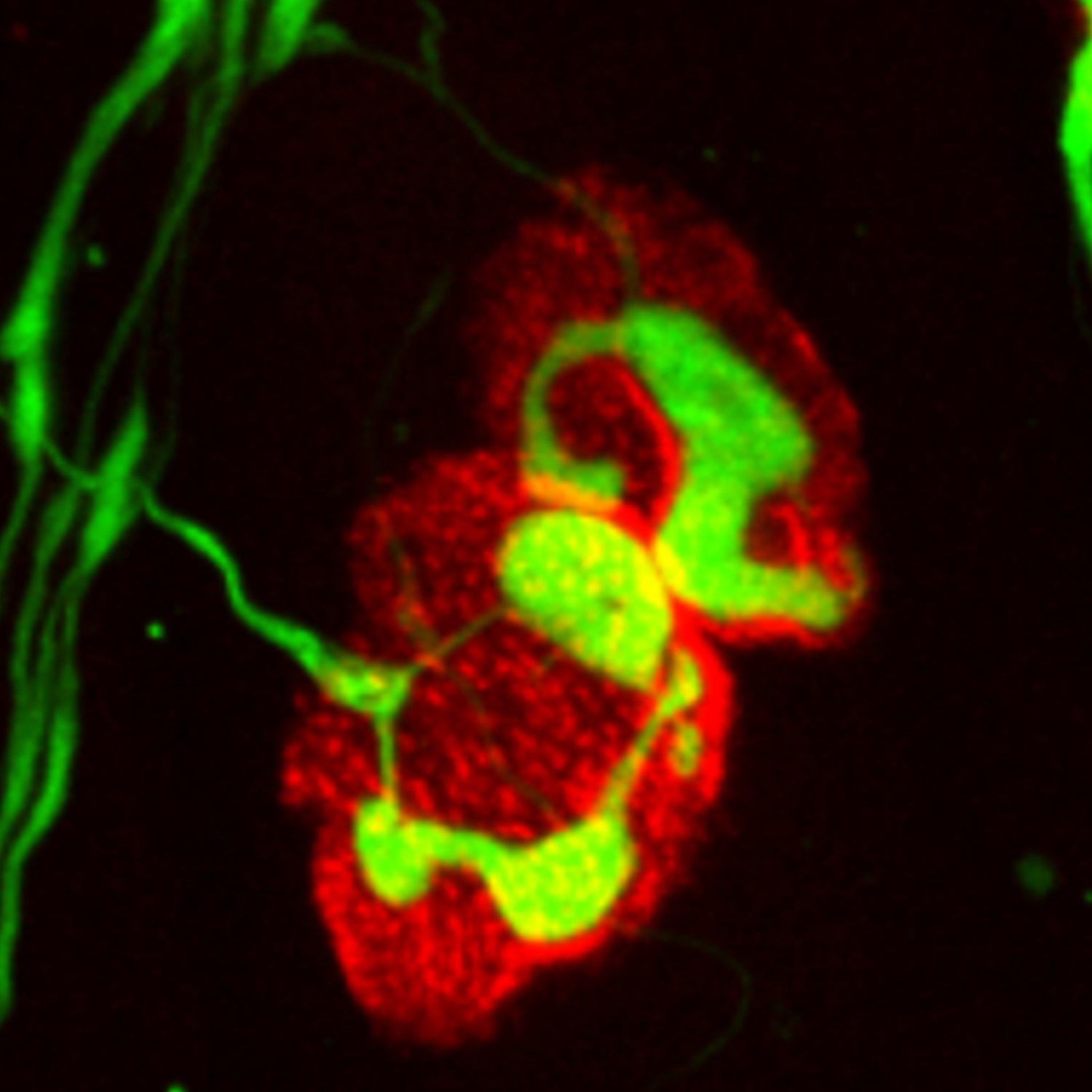

Supplement: Supplementary file 6 — Source Data Fig. 3 [file 44321_2024_37_MOESM6_ESM.zip › Fig 3/Fig3d/Figure 3d_Denervated Small_Merge.tif]

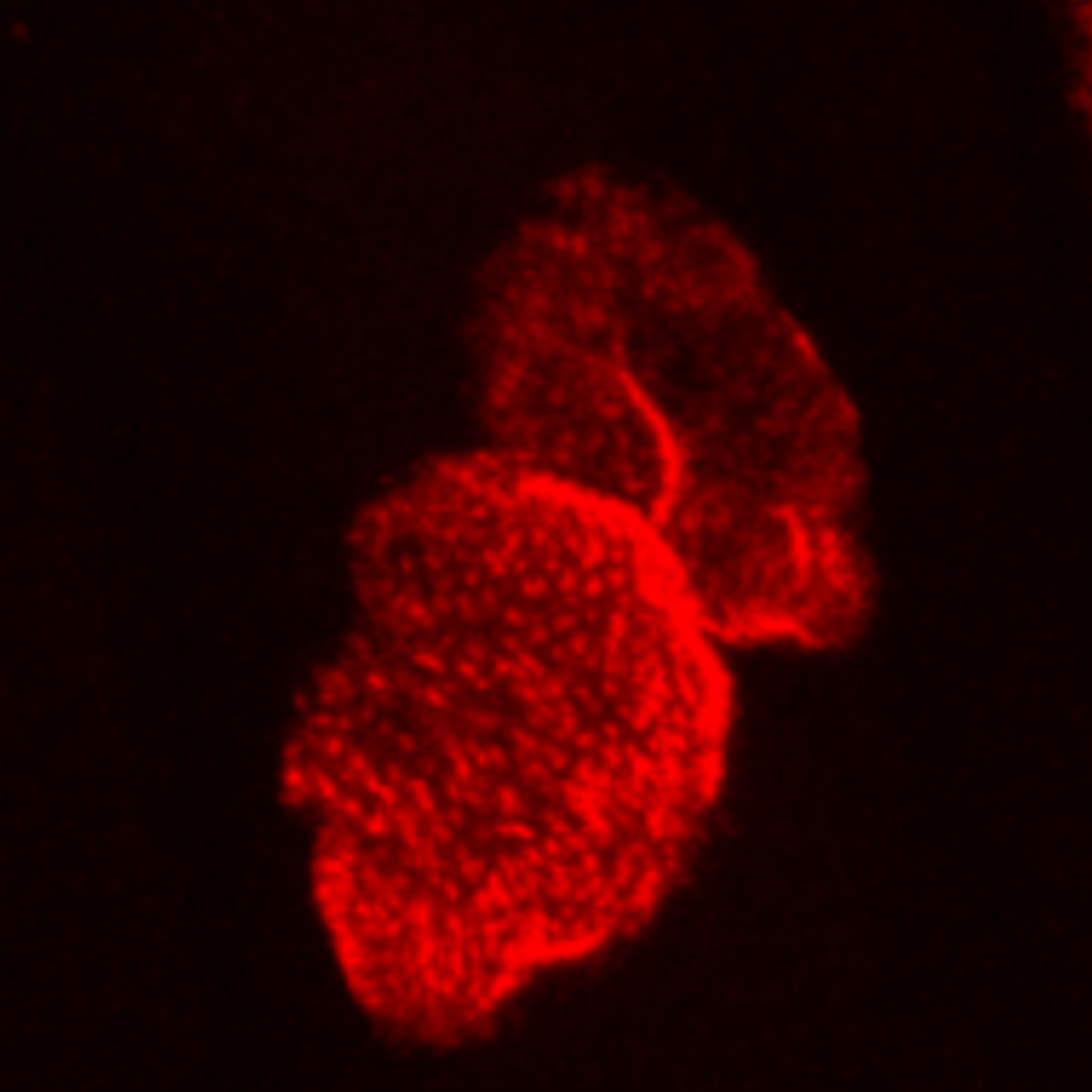

Supplement: Supplementary file 6 — Source Data Fig. 3 [file 44321_2024_37_MOESM6_ESM.zip › Fig 3/Fig3d/Figure 3d_Denervated Small_Red.tif]

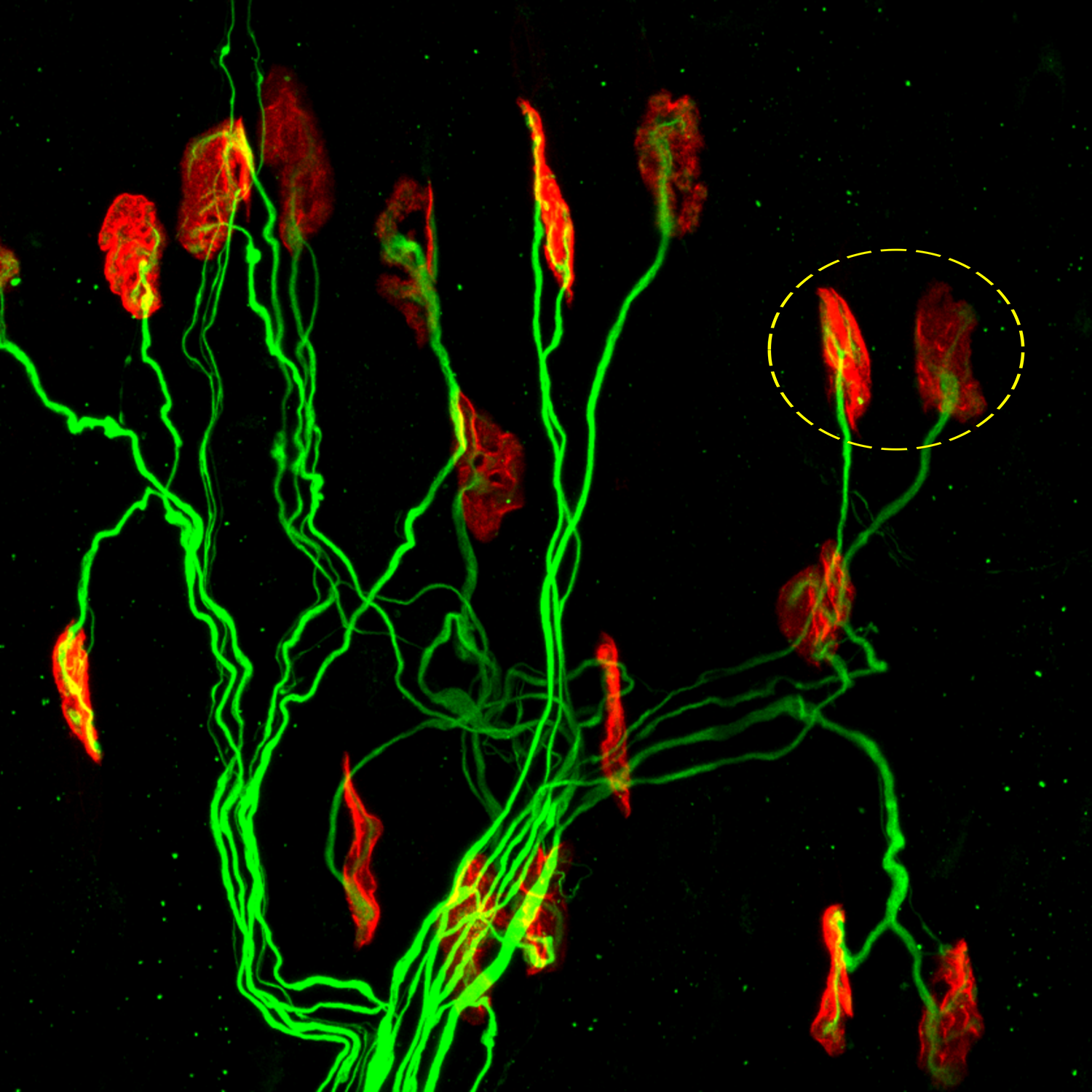

Supplement: Supplementary file 6 — Source Data Fig. 3 [file 44321_2024_37_MOESM6_ESM.zip › Fig 3/Fig3d/Figure 3d_Innervated Large.tif]

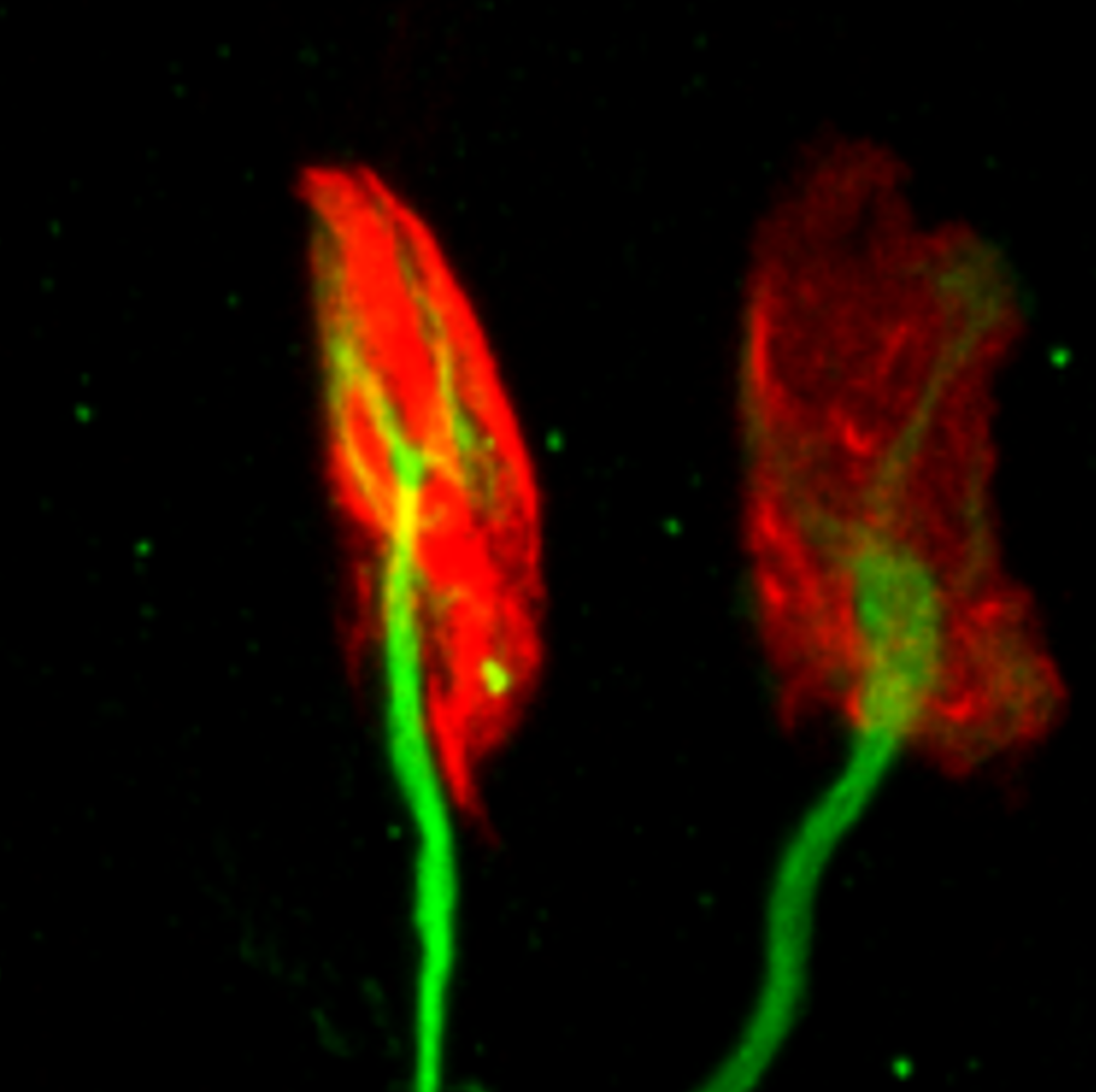

Supplement: Supplementary file 6 — Source Data Fig. 3 [file 44321_2024_37_MOESM6_ESM.zip › Fig 3/Fig3d/Figure 3d_Innervated Large_Merge.tif]

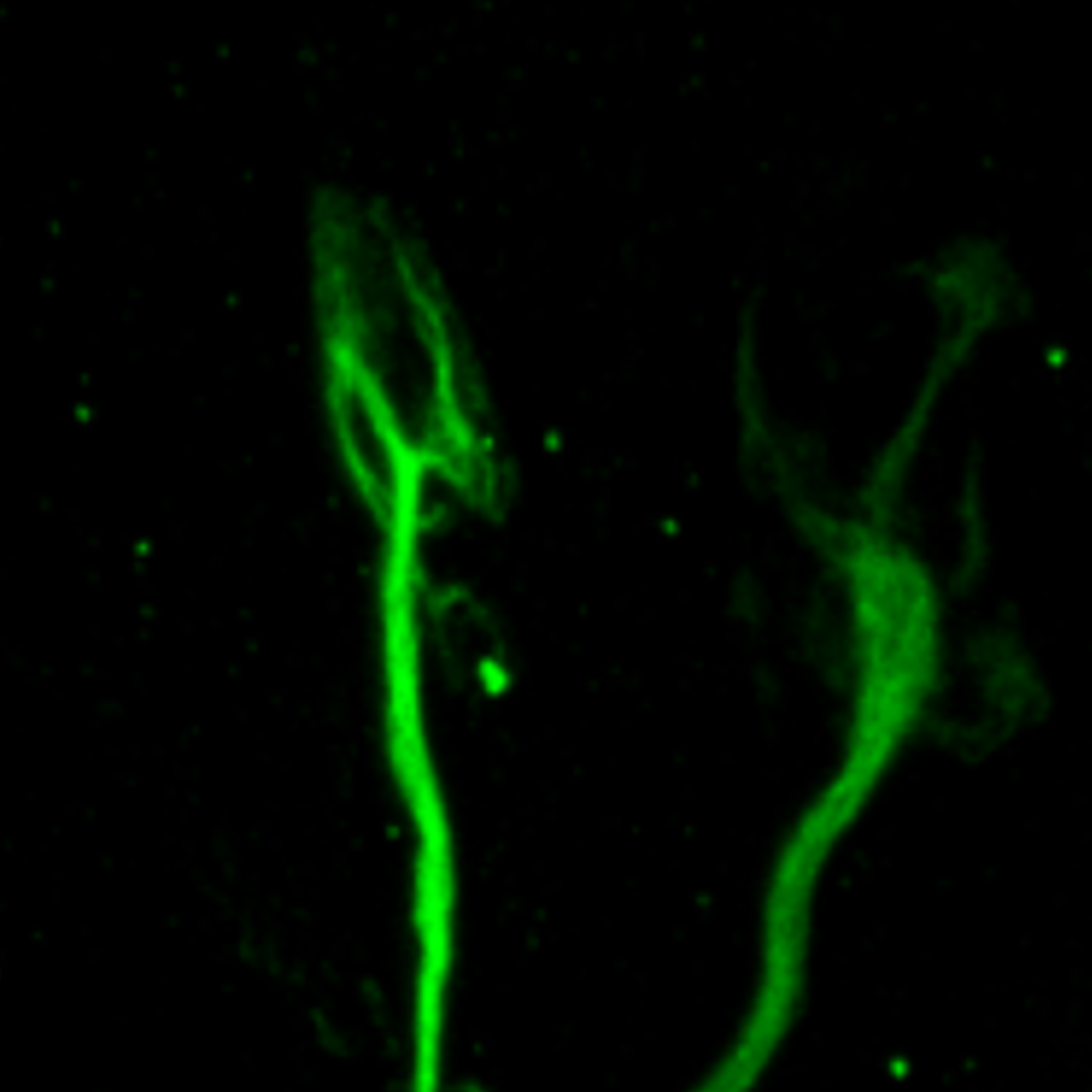

Supplement: Supplementary file 6 — Source Data Fig. 3 [file 44321_2024_37_MOESM6_ESM.zip › Fig 3/Fig3d/Figure 3d_Innervated Small_Green.tif]

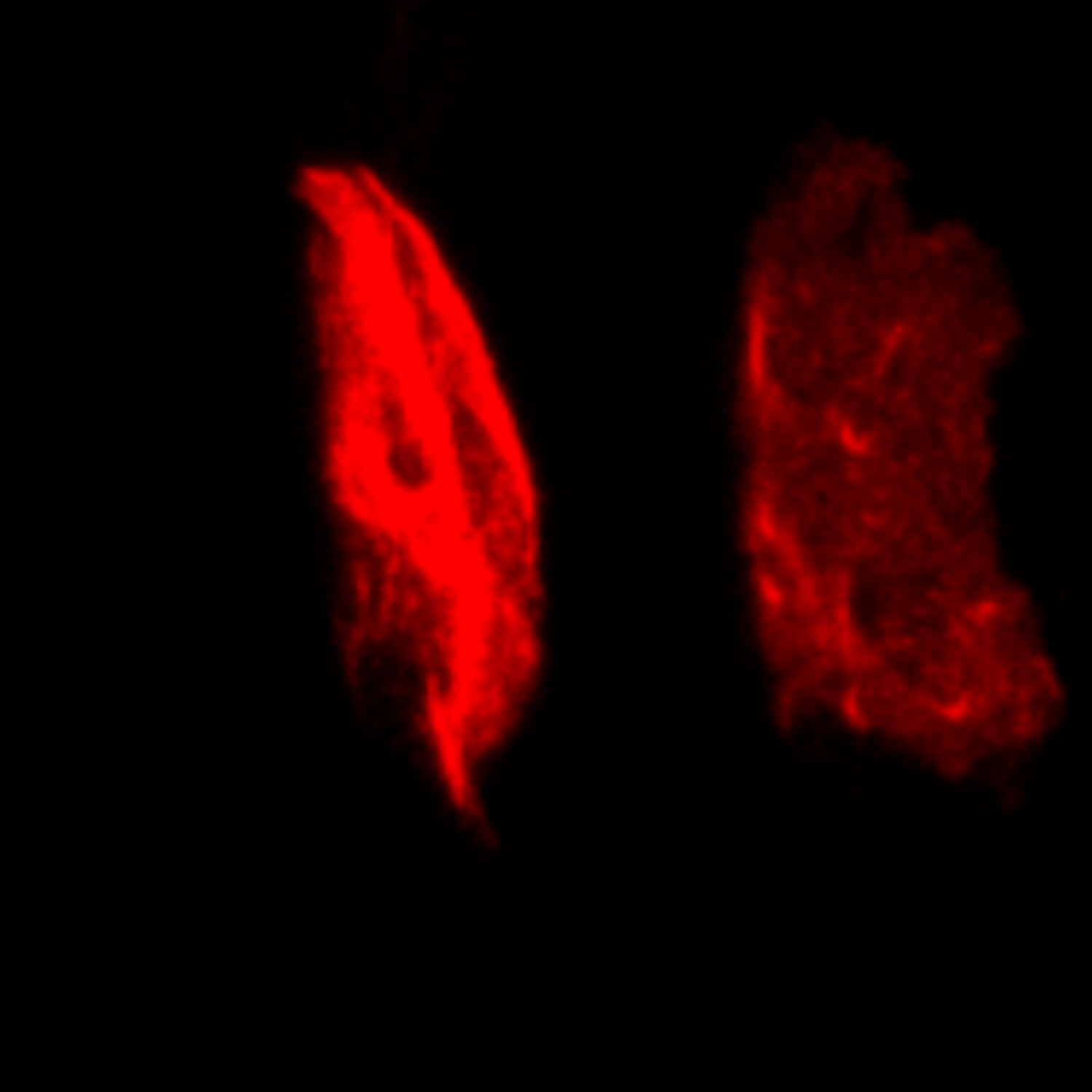

Supplement: Supplementary file 6 — Source Data Fig. 3 [file 44321_2024_37_MOESM6_ESM.zip › Fig 3/Fig3d/Figure 3d_Innervated Small_Red.tif]

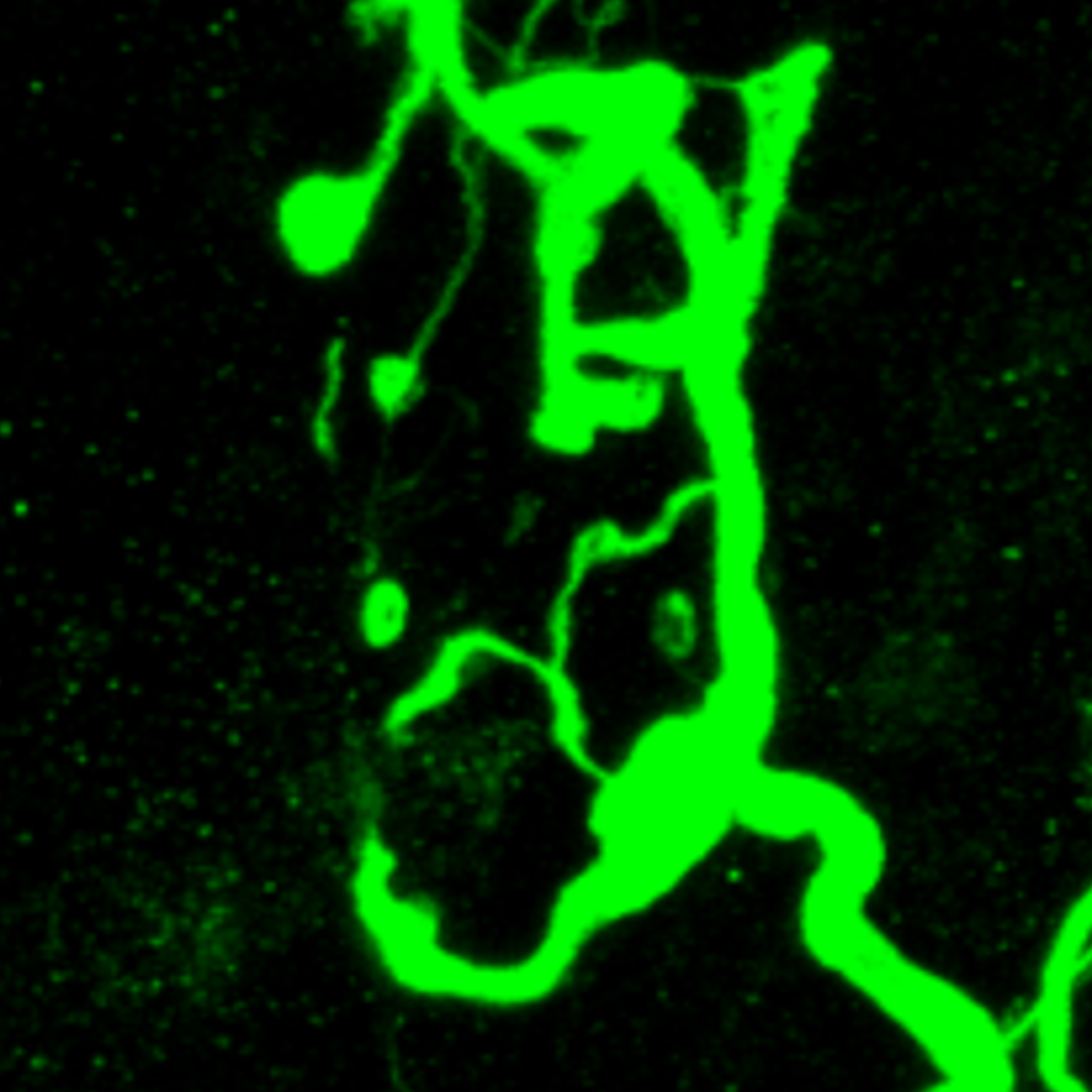

Supplement: Supplementary file 6 — Source Data Fig. 3 [file 44321_2024_37_MOESM6_ESM.zip › Fig 3/Fig3d/Figure 3d_Partially innervated Small_Green.tif]

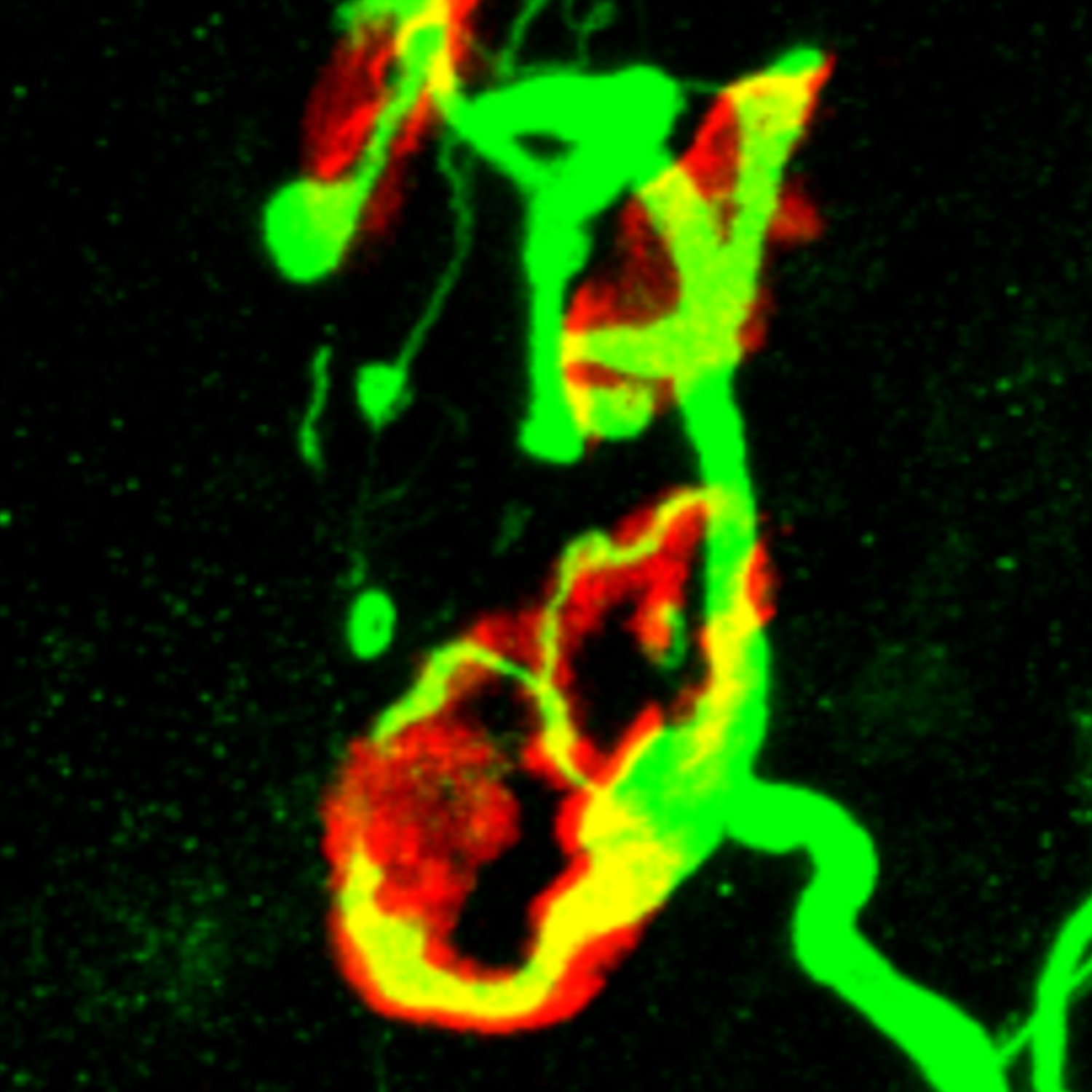

Supplement: Supplementary file 6 — Source Data Fig. 3 [file 44321_2024_37_MOESM6_ESM.zip › Fig 3/Fig3d/Figure 3d_Partially innervated Small_Merge.tif]

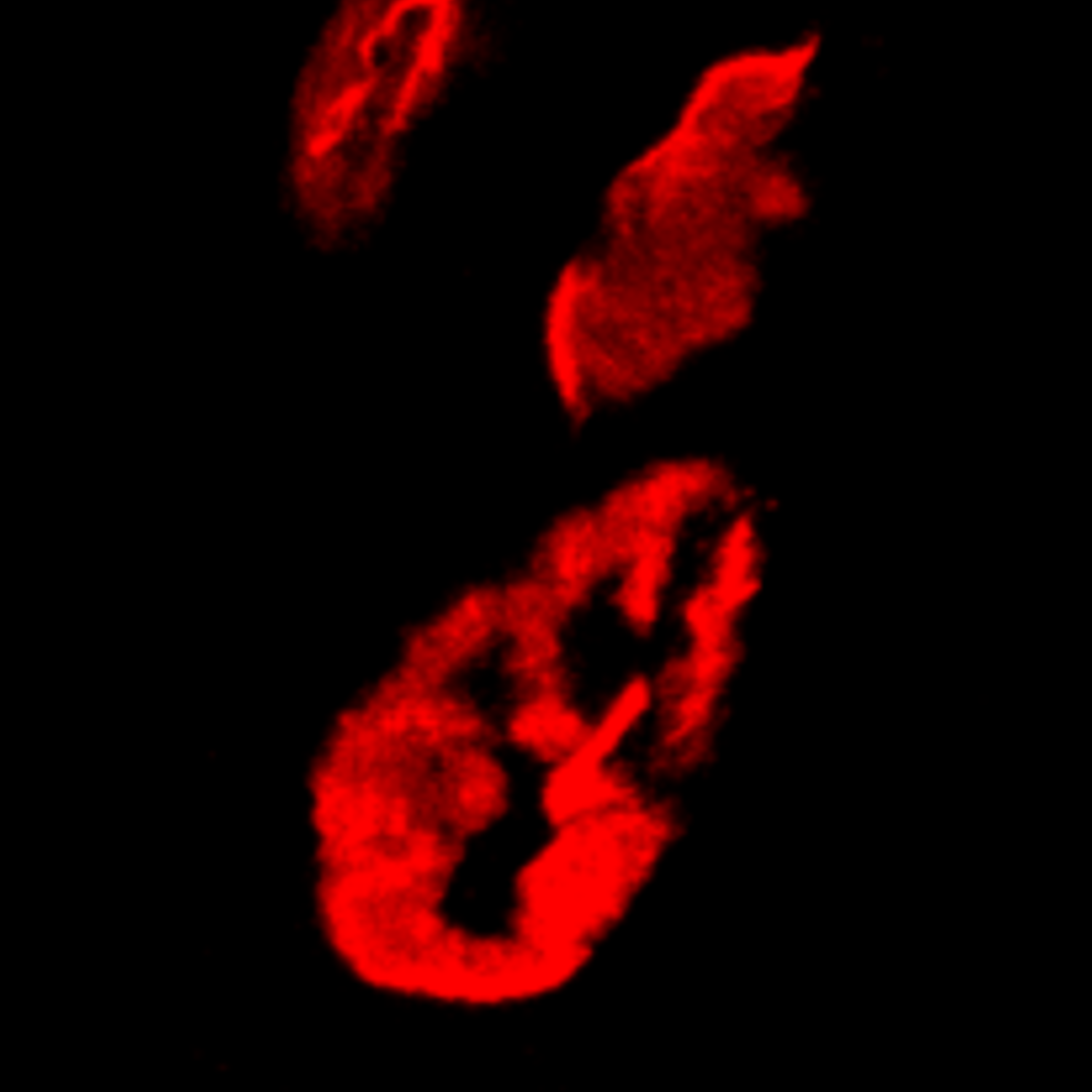

Supplement: Supplementary file 6 — Source Data Fig. 3 [file 44321_2024_37_MOESM6_ESM.zip › Fig 3/Fig3d/Figure 3d_Partially innervated Small_Red.tif]

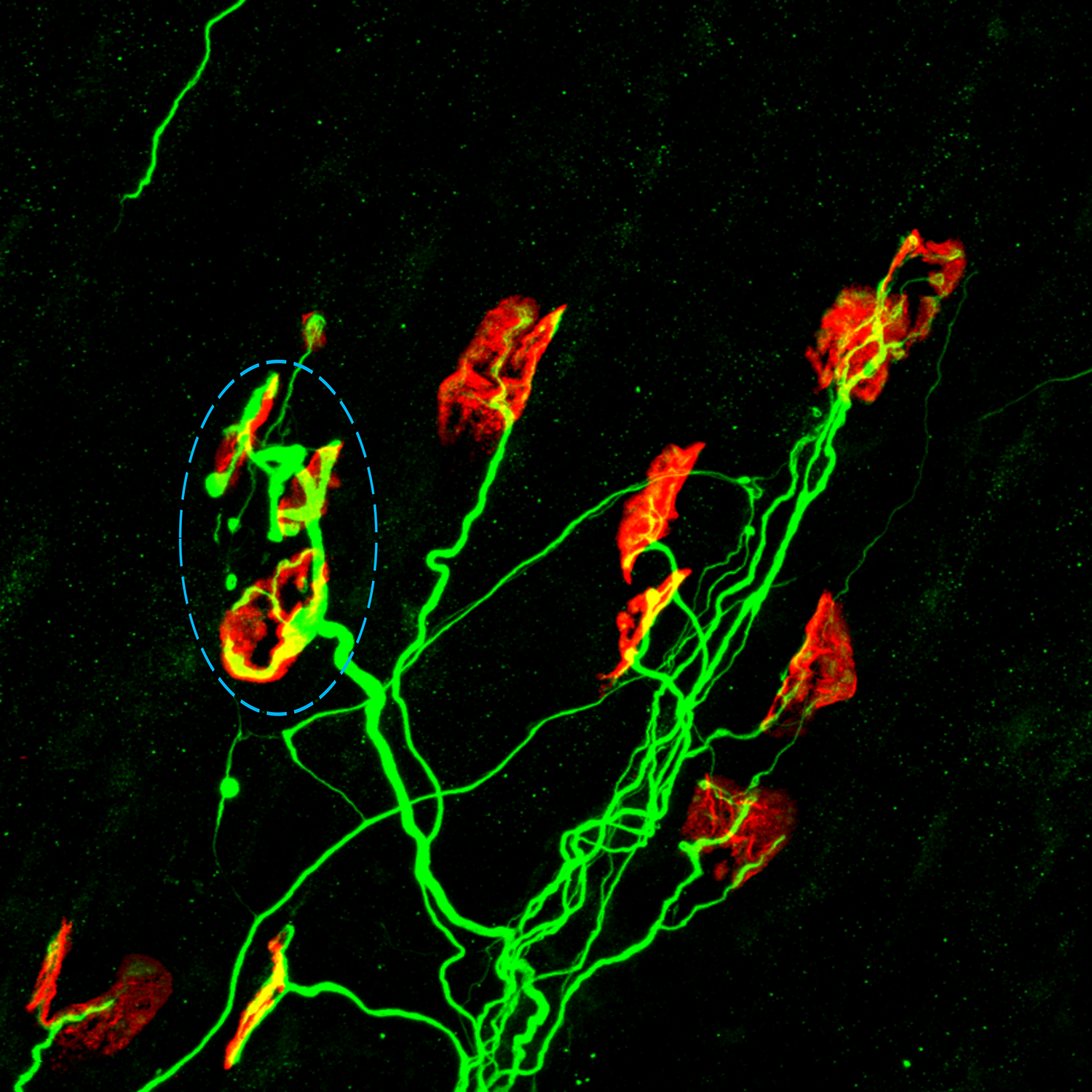

Supplement: Supplementary file 6 — Source Data Fig. 3 [file 44321_2024_37_MOESM6_ESM.zip › Fig 3/Fig3d/Figure 3d_Partially innervated.tif]

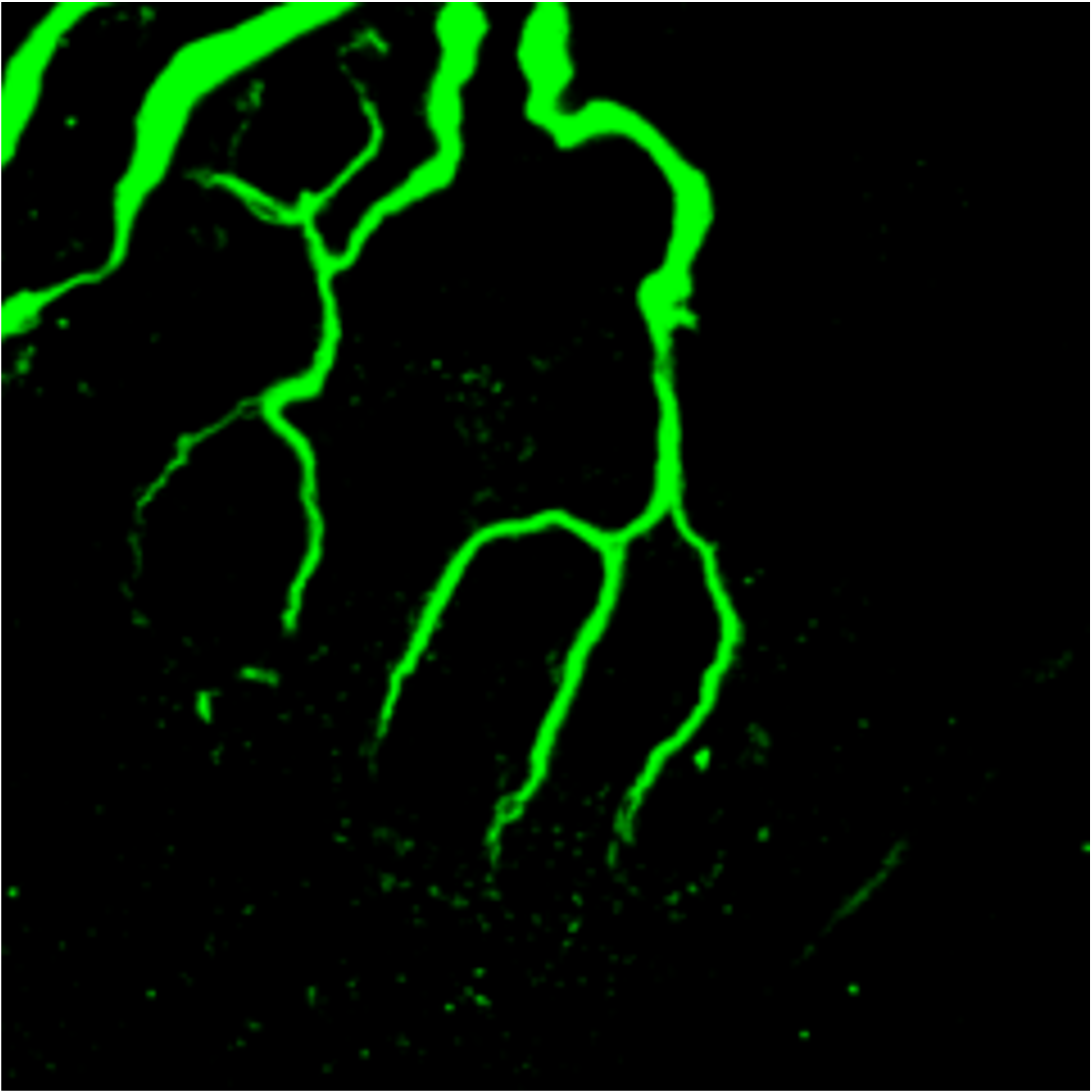

Supplement: Supplementary file 6 — Source Data Fig. 3 [file 44321_2024_37_MOESM6_ESM.zip › Fig 3/Fig3f/Figure 3f 2nd gen Green.png]

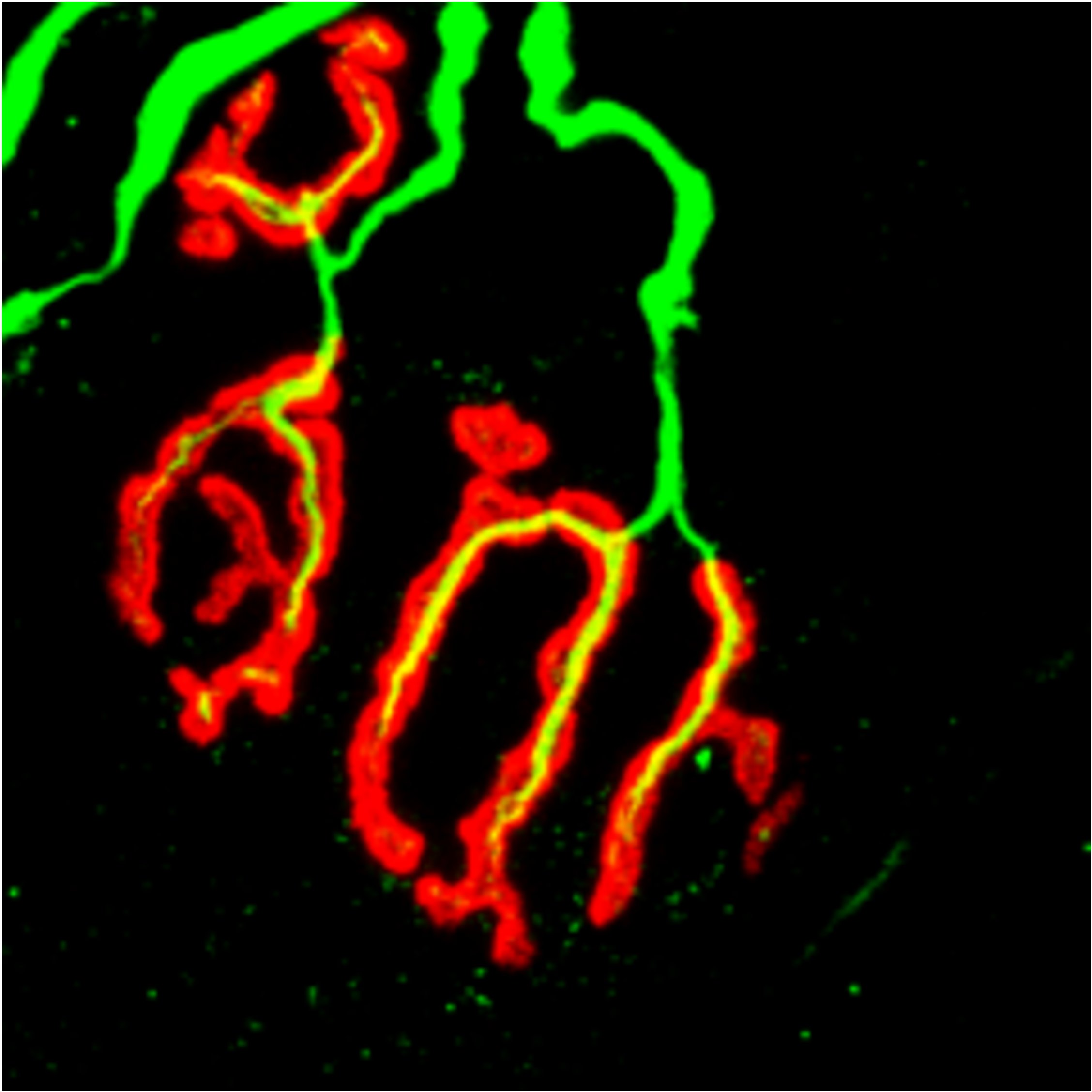

Supplement: Supplementary file 6 — Source Data Fig. 3 [file 44321_2024_37_MOESM6_ESM.zip › Fig 3/Fig3f/Figure 3f 2nd gen merge.png]

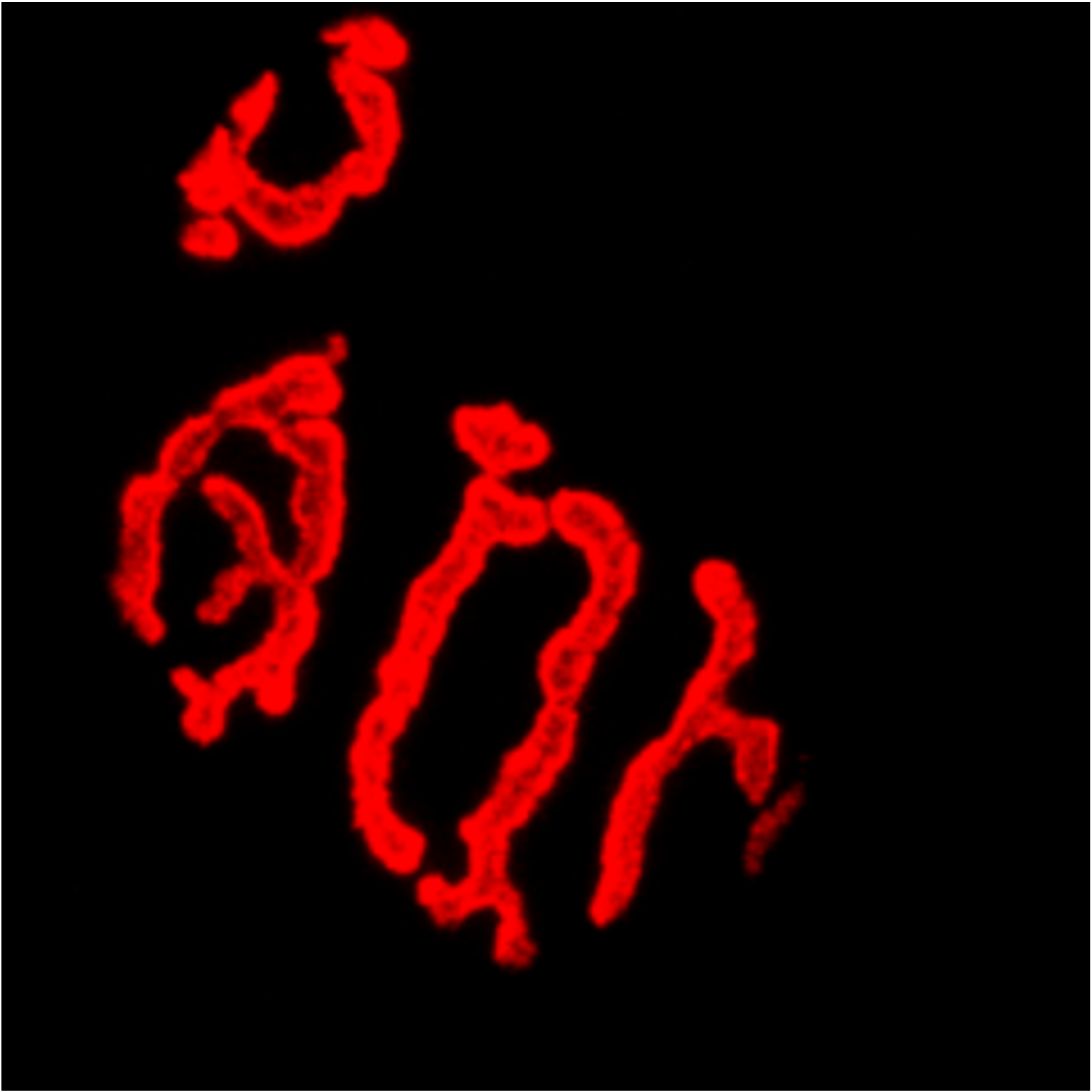

Supplement: Supplementary file 6 — Source Data Fig. 3 [file 44321_2024_37_MOESM6_ESM.zip › Fig 3/Fig3f/Figure 3f 2nd gen Red.png]

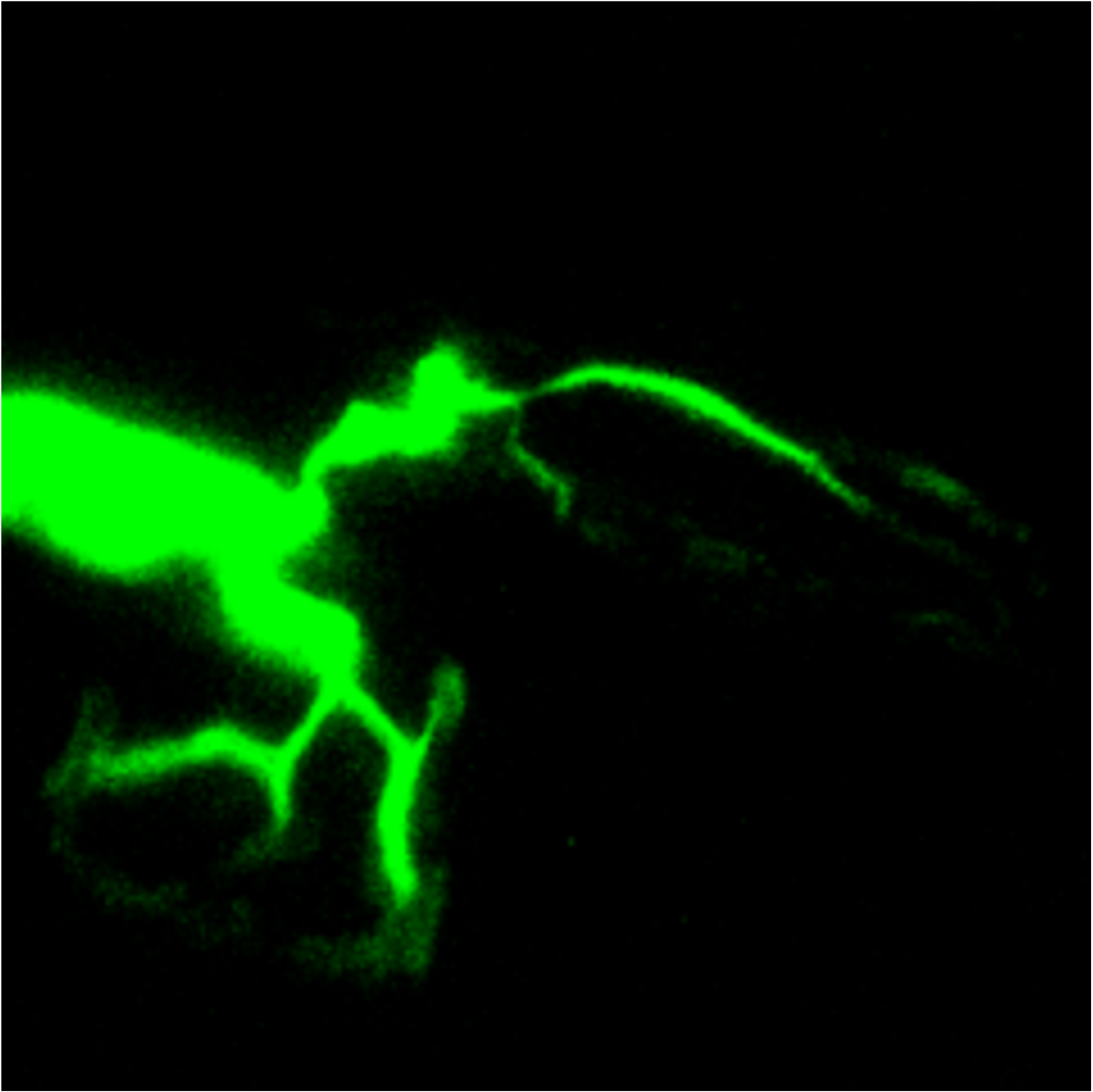

Supplement: Supplementary file 6 — Source Data Fig. 3 [file 44321_2024_37_MOESM6_ESM.zip › Fig 3/Fig3f/Figure 3f BMK green.png]

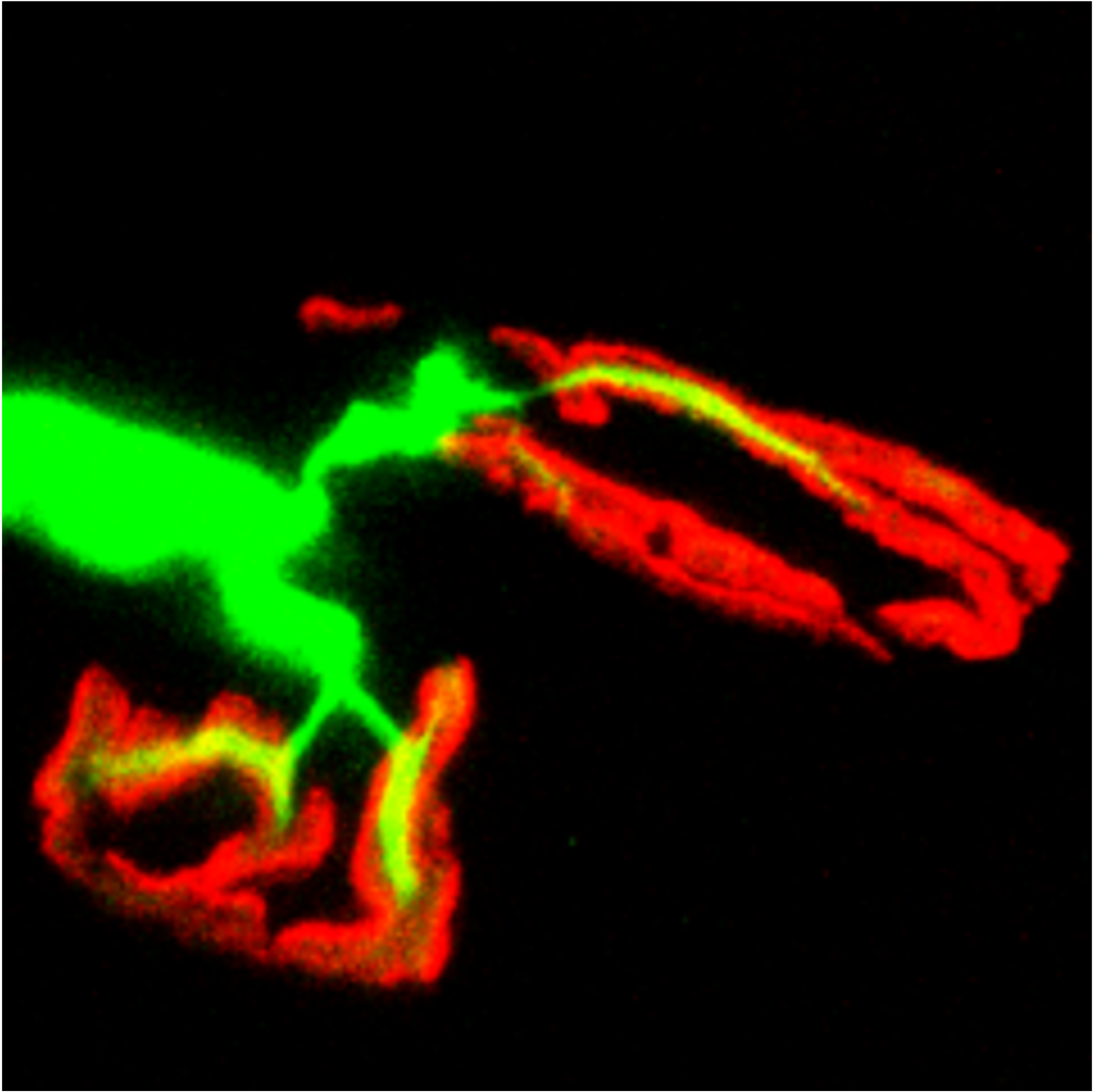

Supplement: Supplementary file 6 — Source Data Fig. 3 [file 44321_2024_37_MOESM6_ESM.zip › Fig 3/Fig3f/Figure 3f BMK merge.png]

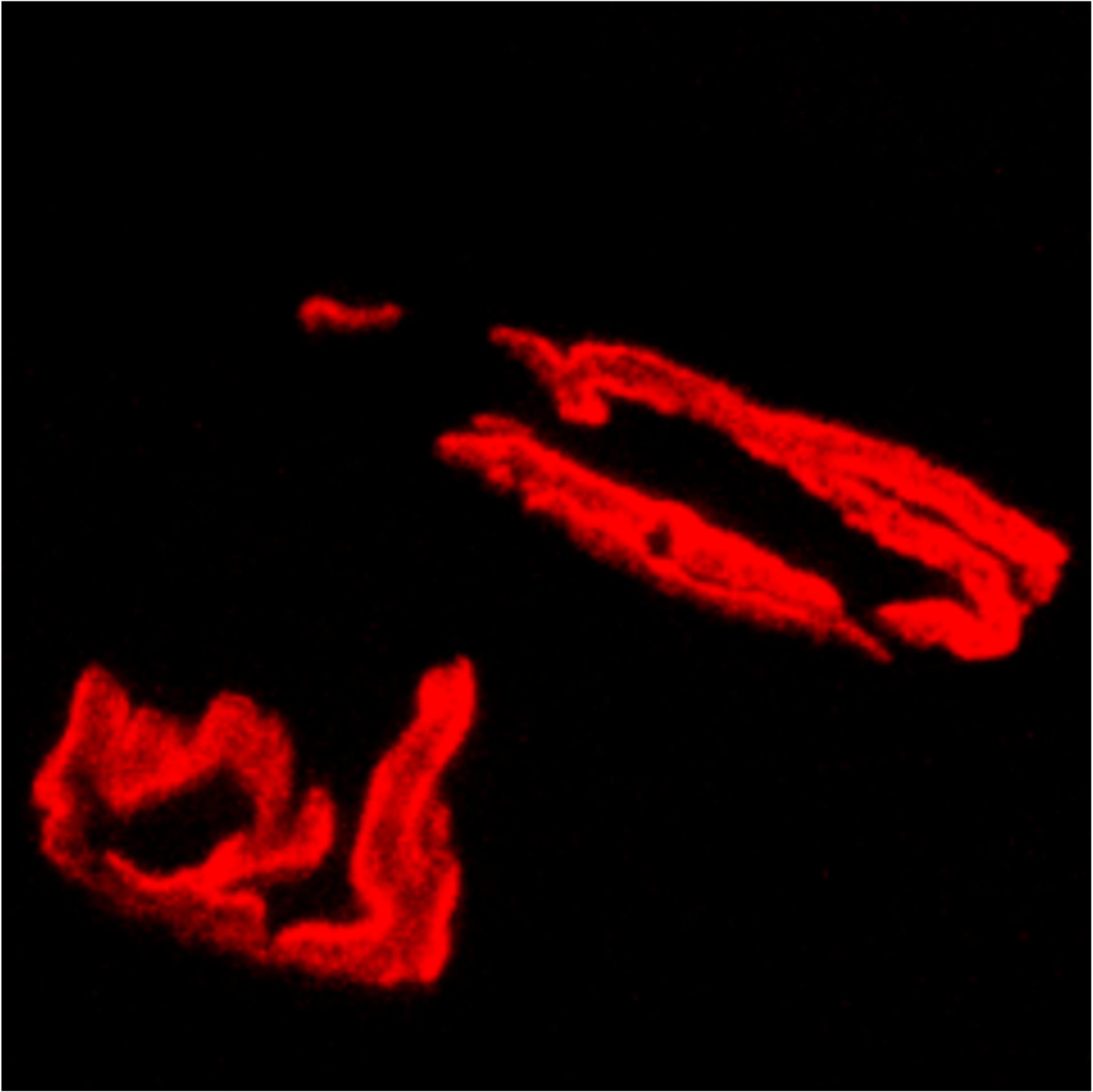

Supplement: Supplementary file 6 — Source Data Fig. 3 [file 44321_2024_37_MOESM6_ESM.zip › Fig 3/Fig3f/Figure 3f BMK red.png]

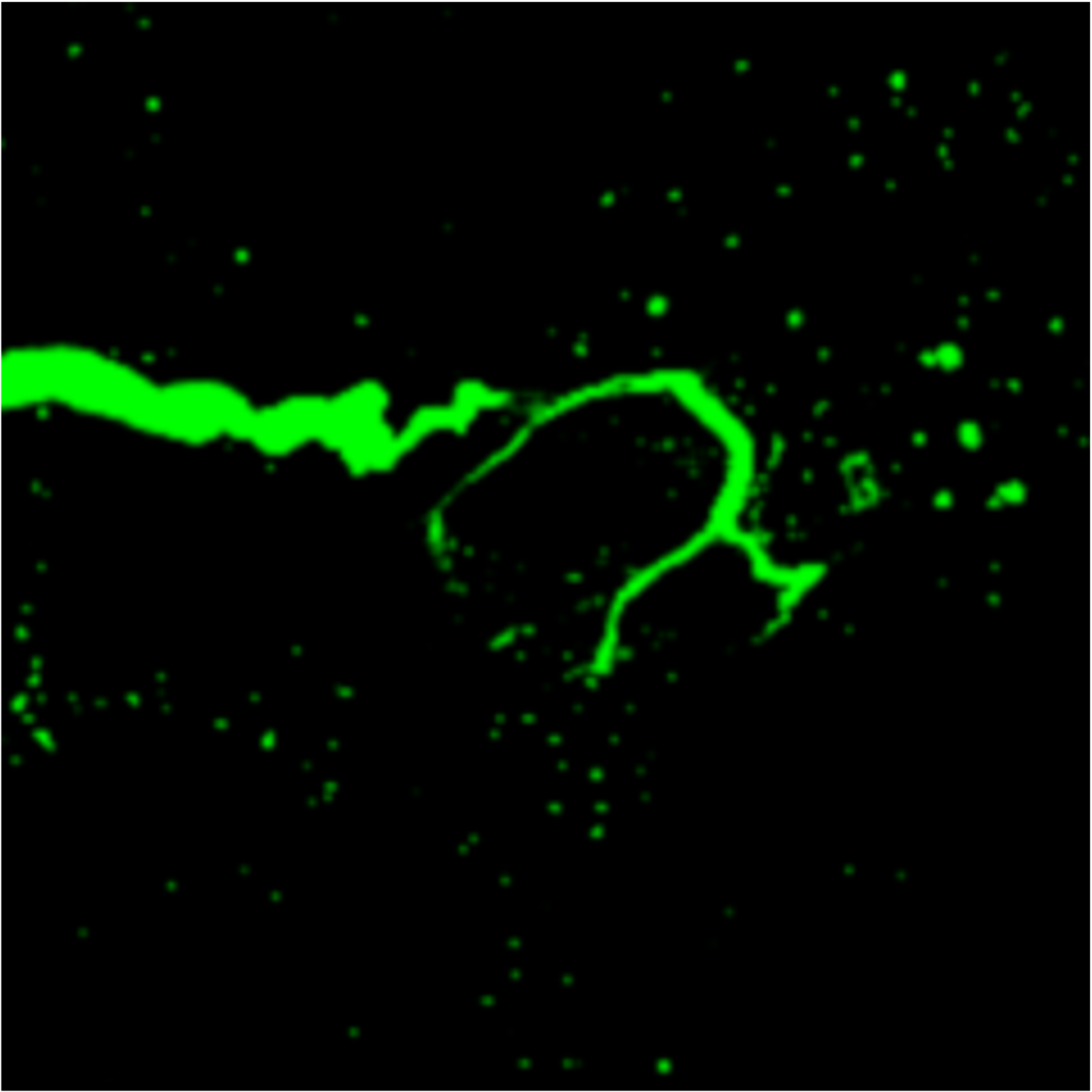

Supplement: Supplementary file 6 — Source Data Fig. 3 [file 44321_2024_37_MOESM6_ESM.zip › Fig 3/Fig3f/Figure 3f HC green.png]

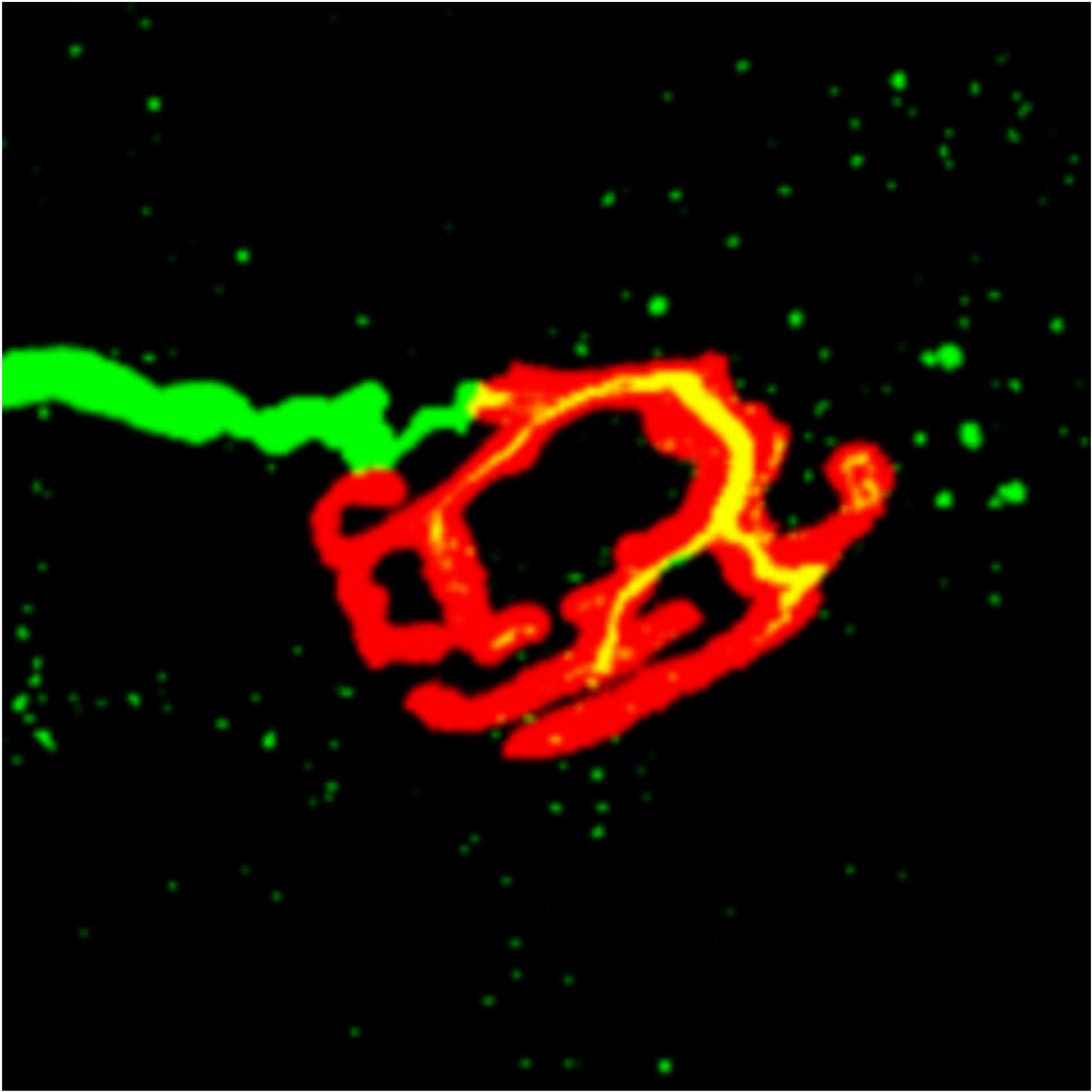

Supplement: Supplementary file 6 — Source Data Fig. 3 [file 44321_2024_37_MOESM6_ESM.zip › Fig 3/Fig3f/Figure 3f HC merge.png]

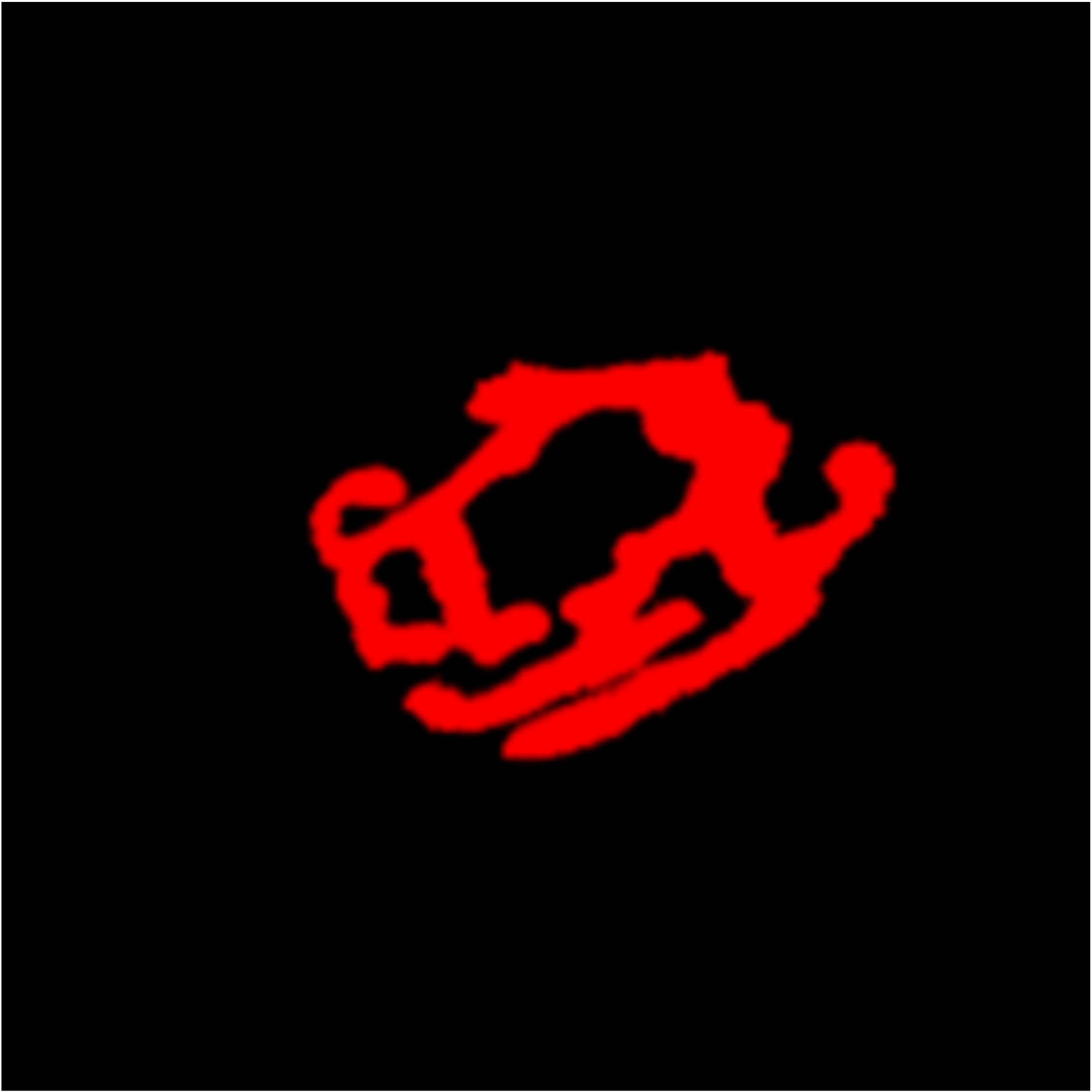

Supplement: Supplementary file 6 — Source Data Fig. 3 [file 44321_2024_37_MOESM6_ESM.zip › Fig 3/Fig3f/Figure 3f HC red.png]

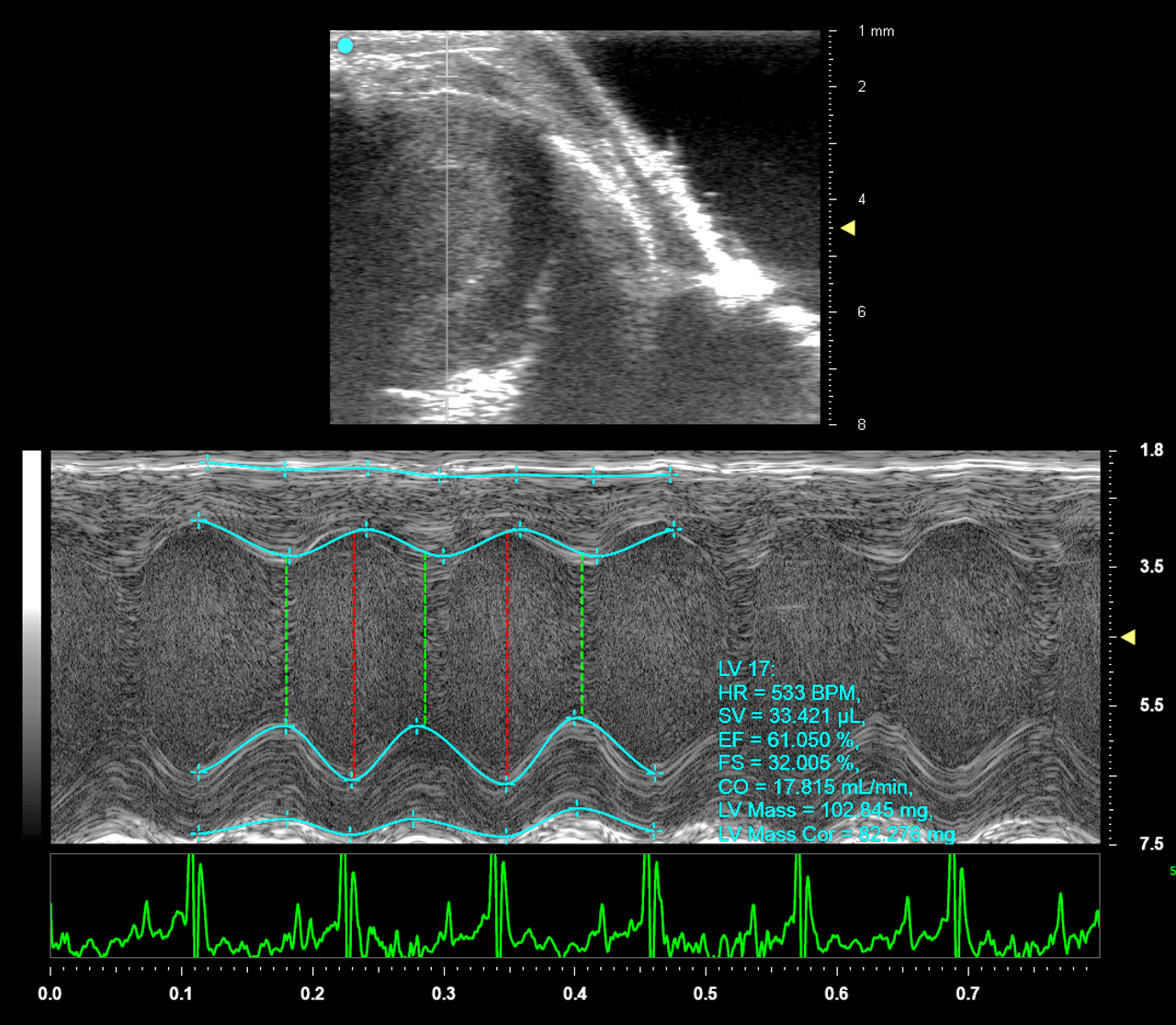

Supplement: Supplementary file 7 — Source Data Fig. 4 [file 44321_2024_37_MOESM7_ESM.zip › Fig 4/Fig4b/figure 4b 2nd.tif.png]

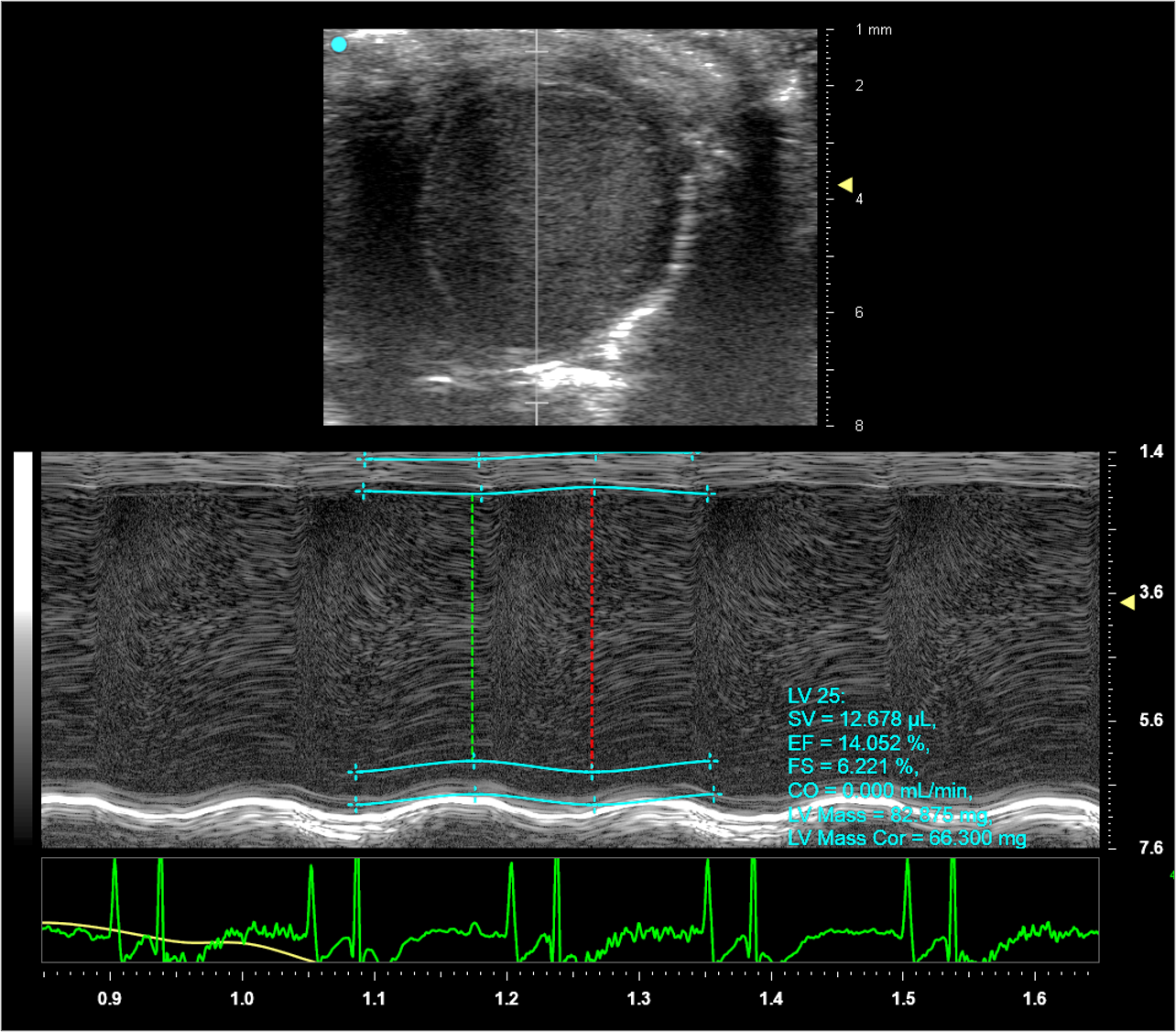

Supplement: Supplementary file 7 — Source Data Fig. 4 [file 44321_2024_37_MOESM7_ESM.zip › Fig 4/Fig4b/figure 4b benchmark.tif.png]

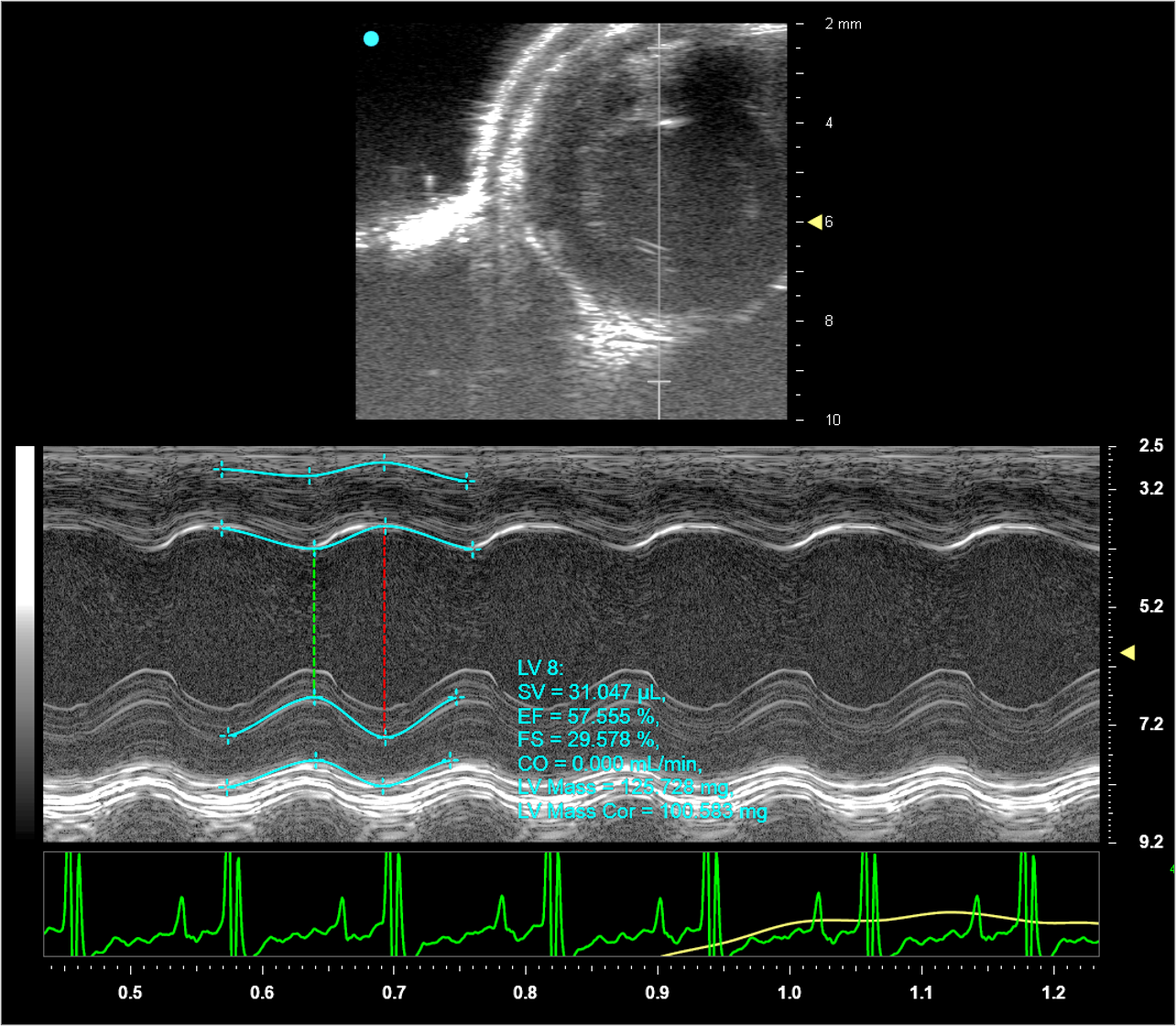

Supplement: Supplementary file 7 — Source Data Fig. 4 [file 44321_2024_37_MOESM7_ESM.zip › Fig 4/Fig4b/figure 4b healthy carrier.tif.png]

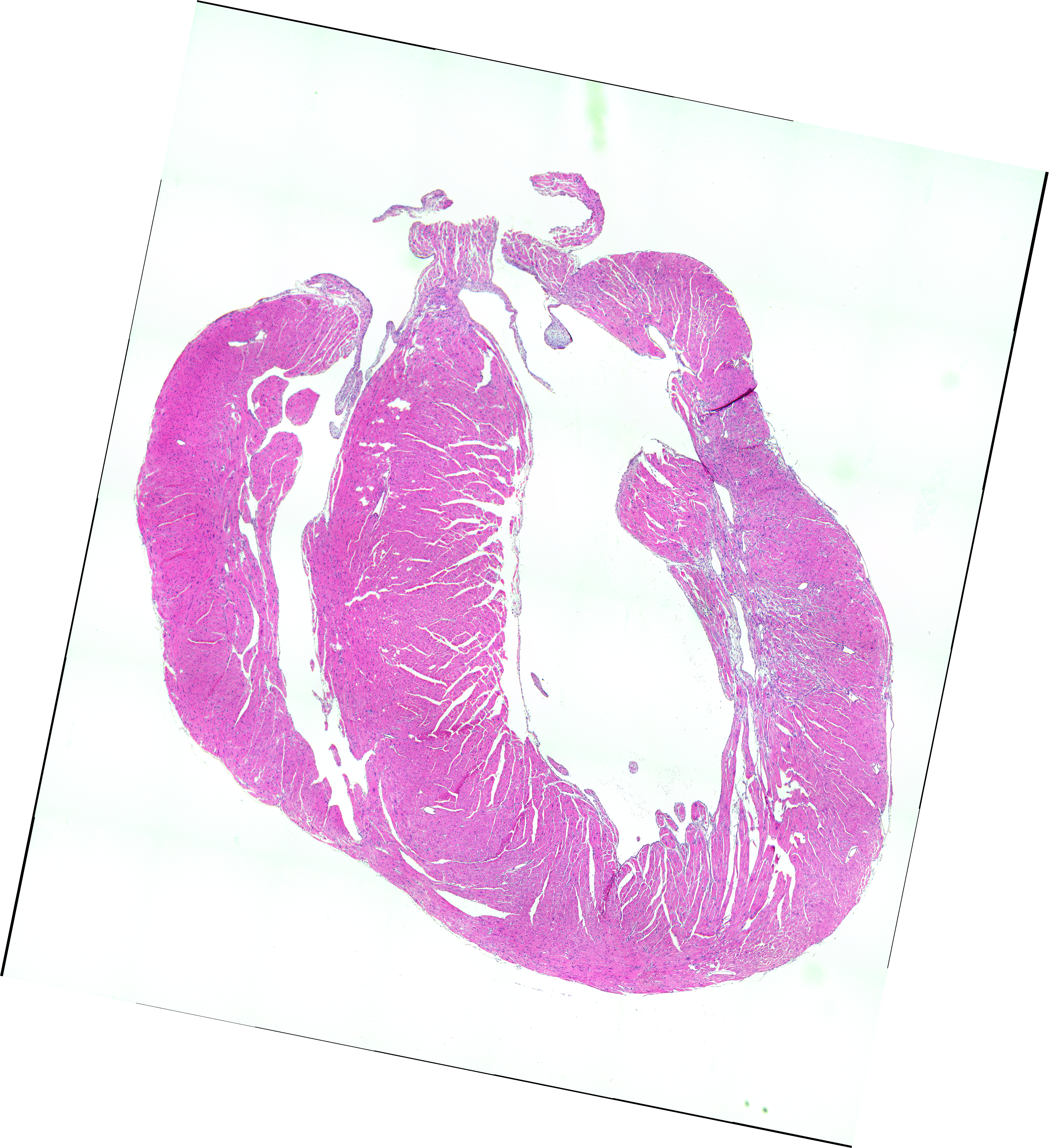

Supplement: Supplementary file 7 — Source Data Fig. 4 [file 44321_2024_37_MOESM7_ESM.zip › Fig 4/Fig4c/figure 4c 2nd.png.tif]

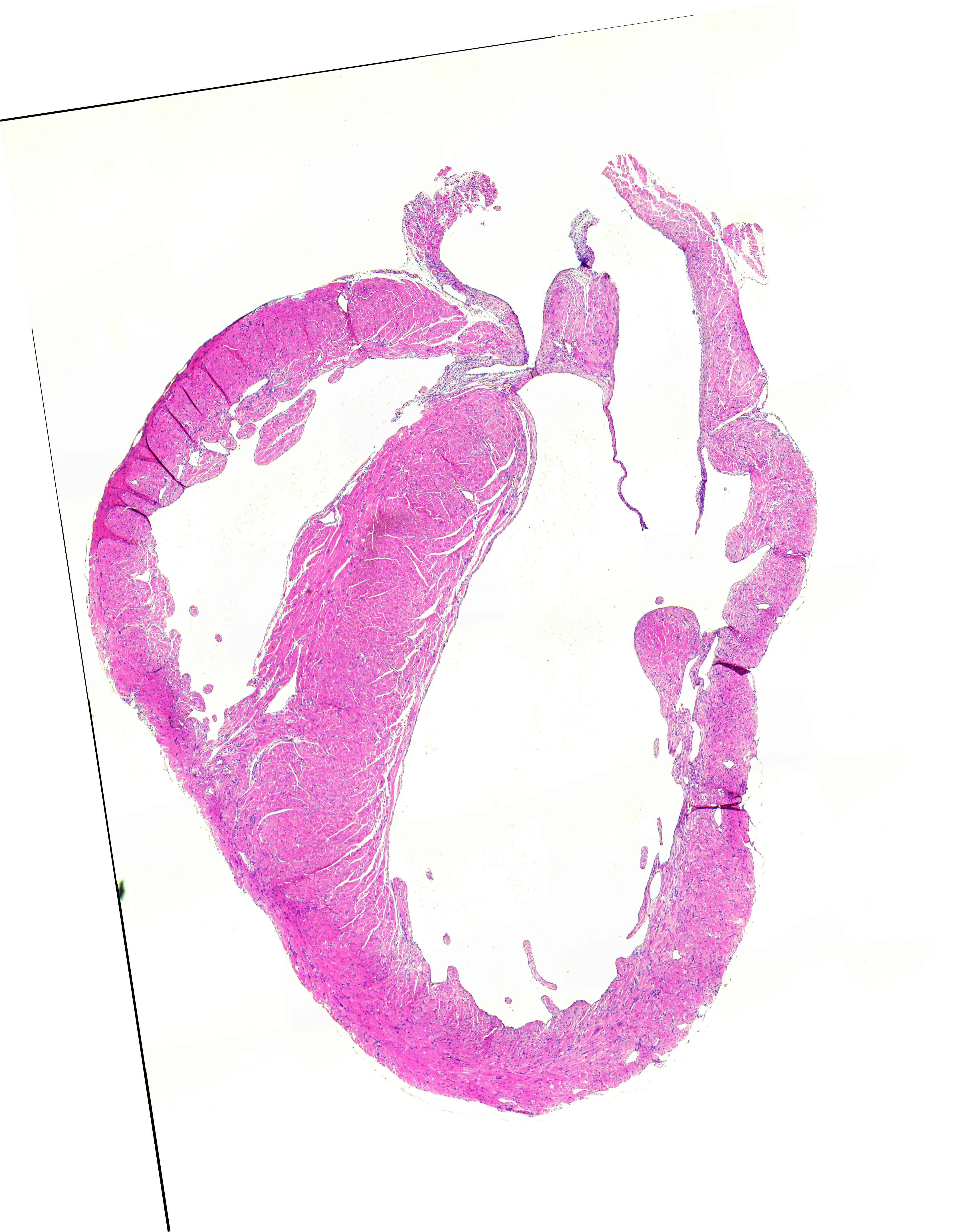

Supplement: Supplementary file 7 — Source Data Fig. 4 [file 44321_2024_37_MOESM7_ESM.zip › Fig 4/Fig4c/figure 4c benchmark.png]

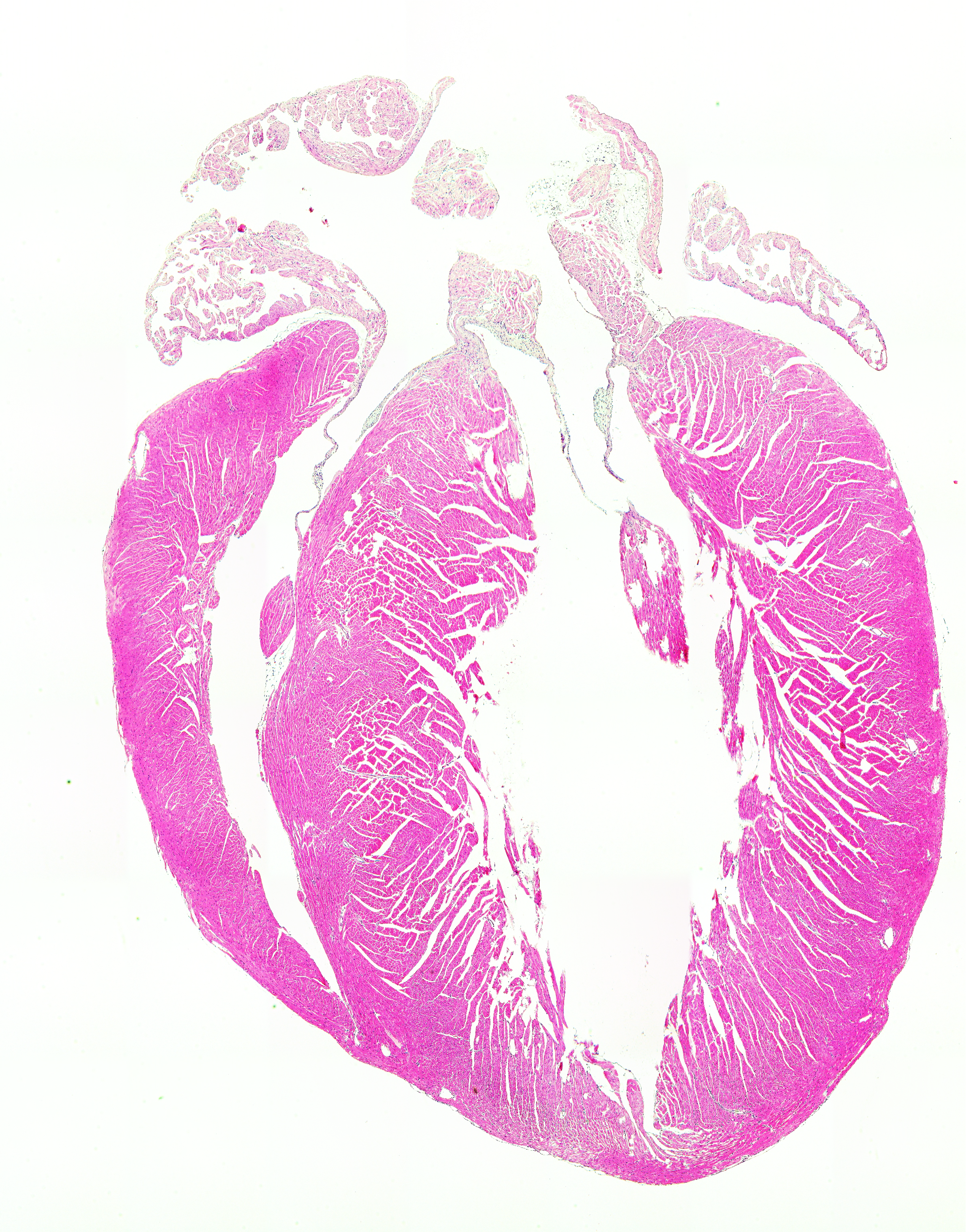

Supplement: Supplementary file 7 — Source Data Fig. 4 [file 44321_2024_37_MOESM7_ESM.zip › Fig 4/Fig4c/figure 4c Healthy carrier.png]

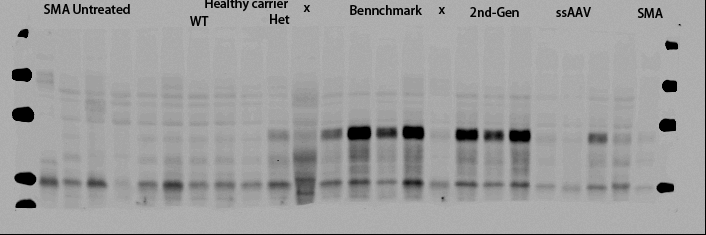

Supplement: Supplementary file 7 — Source Data Fig. 4 [file 44321_2024_37_MOESM7_ESM.zip › Fig 4/Fig4e/Figure 4e 12 days Liver SMN (ref).tif]

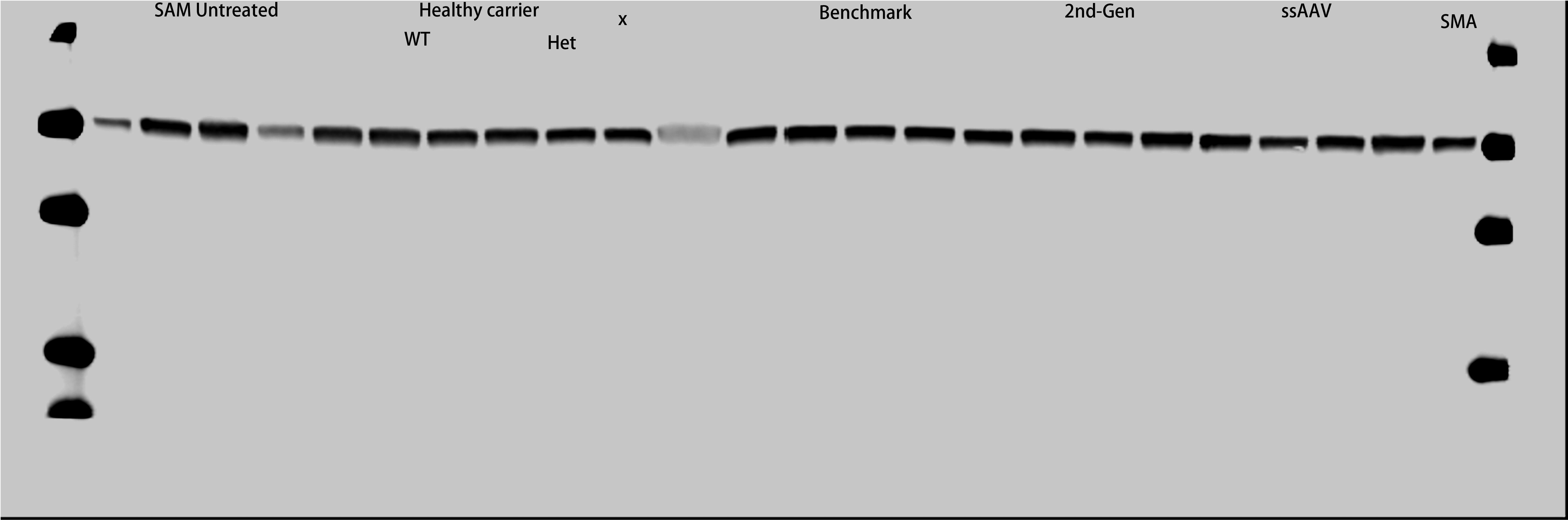

Supplement: Supplementary file 7 — Source Data Fig. 4 [file 44321_2024_37_MOESM7_ESM.zip › Fig 4/Fig4e/Figure 4e 12 days Tubulin (ref).tif]

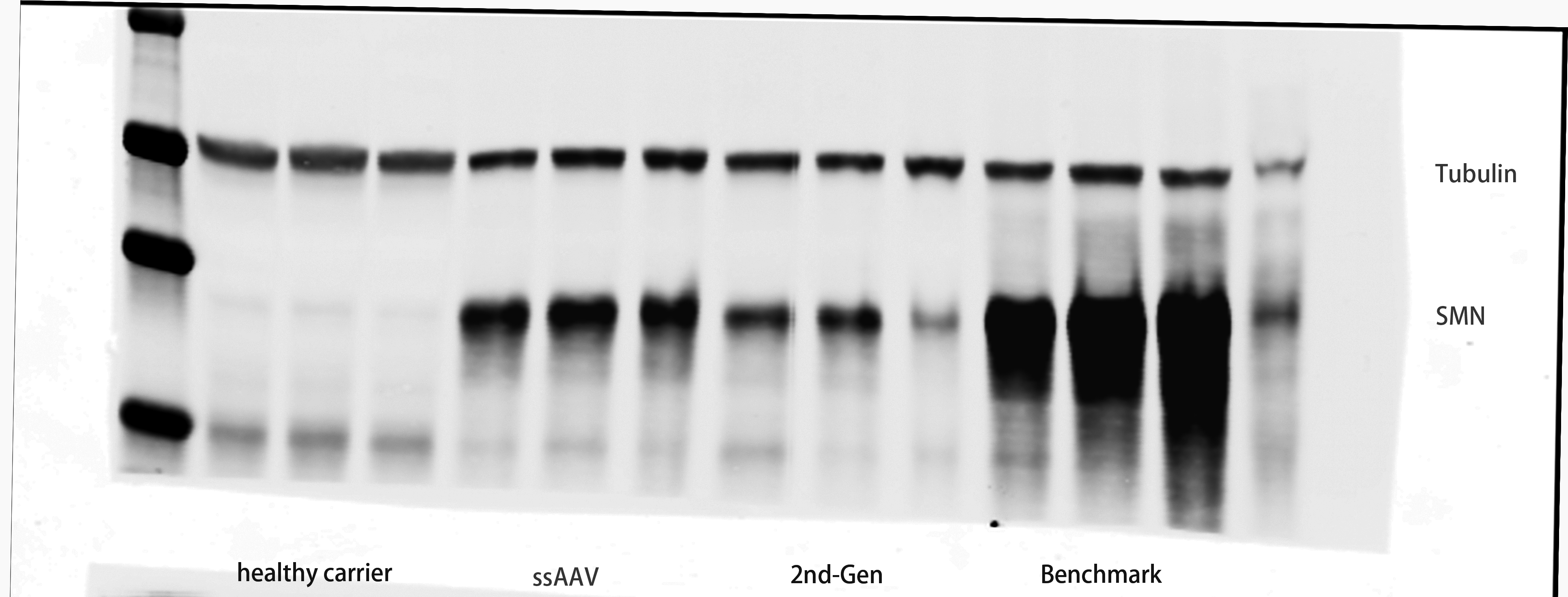

Supplement: Supplementary file 7 — Source Data Fig. 4 [file 44321_2024_37_MOESM7_ESM.zip › Fig 4/Fig4e/Figure 4e 3 days SMN & Tubulin.tif]

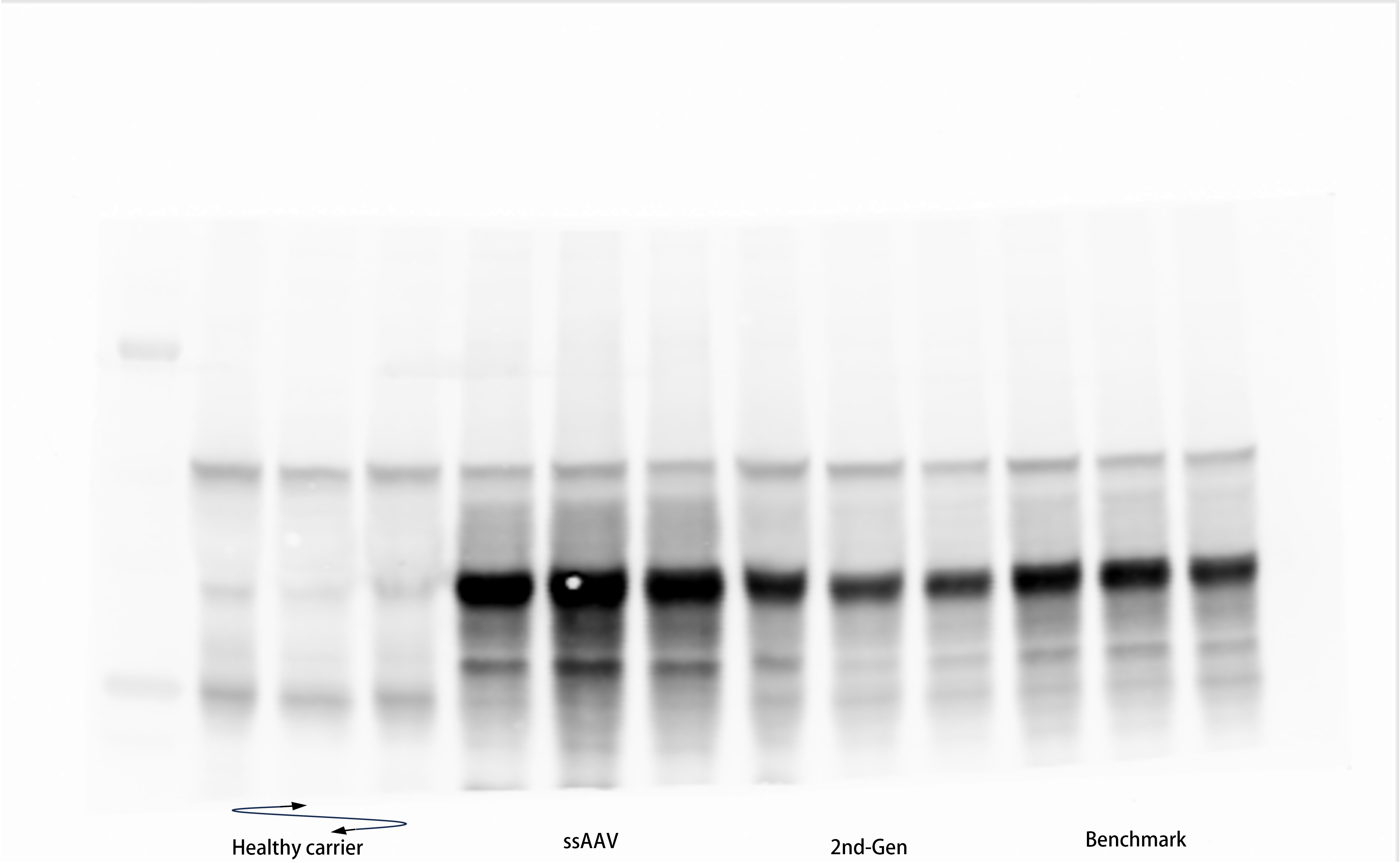

Supplement: Supplementary file 7 — Source Data Fig. 4 [file 44321_2024_37_MOESM7_ESM.zip › Fig 4/Fig4e/Figure 4e 8 days SMN.tif]

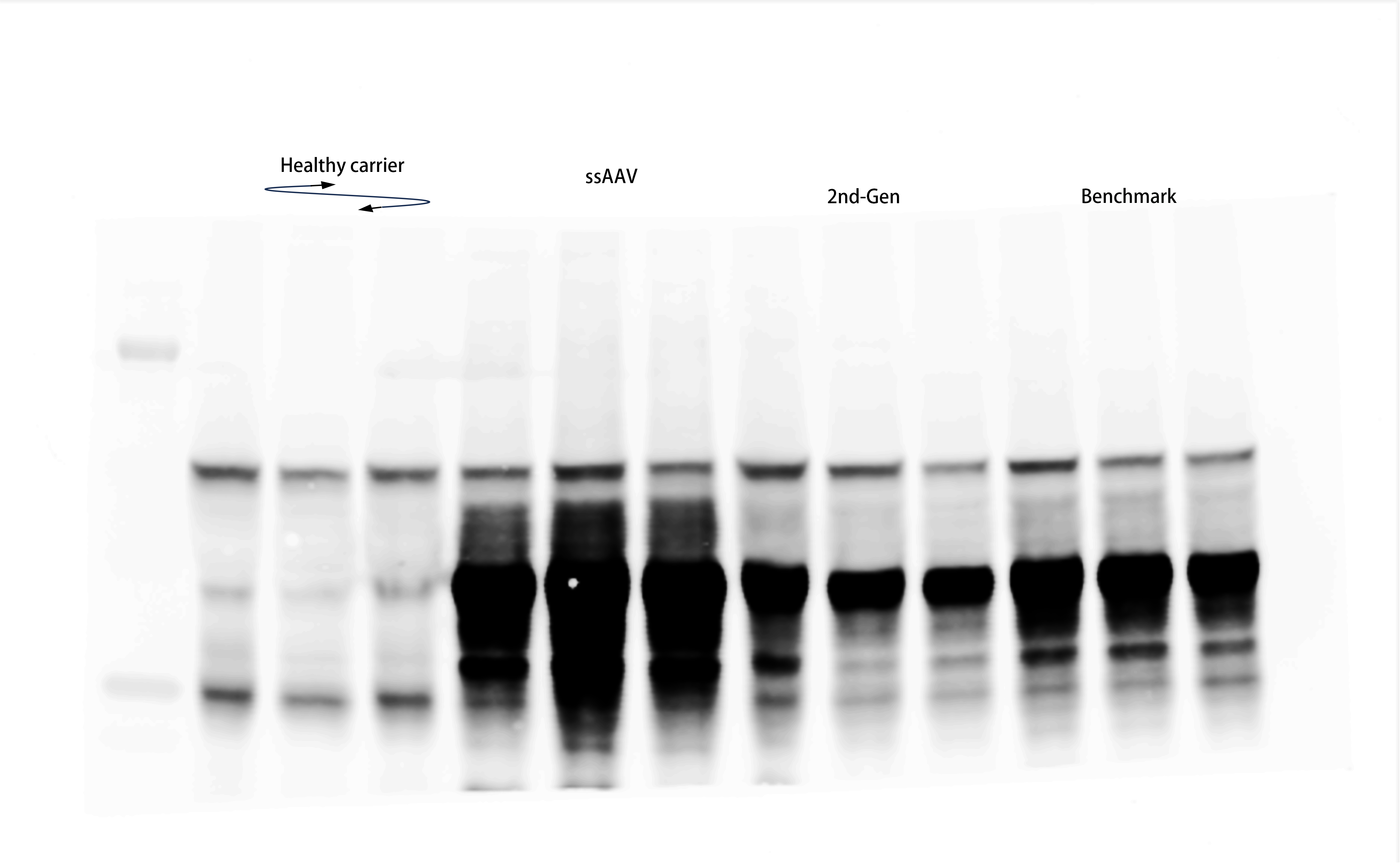

Supplement: Supplementary file 7 — Source Data Fig. 4 [file 44321_2024_37_MOESM7_ESM.zip › Fig 4/Fig4e/Figure 4e 8 days Tubulin.tif]

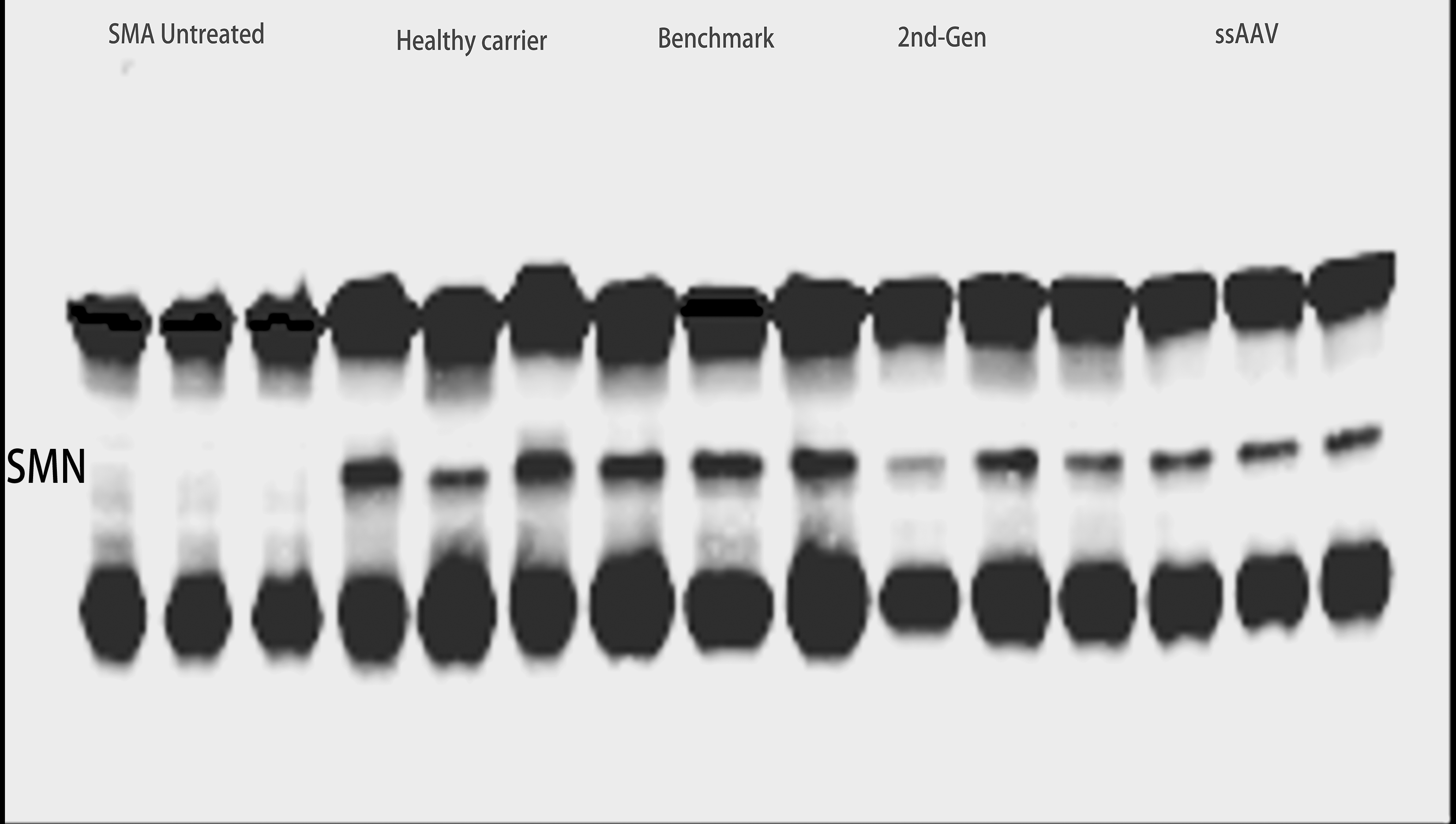

Supplement: Supplementary file 7 — Source Data Fig. 4 [file 44321_2024_37_MOESM7_ESM.zip › Fig 4/Fig4e/Figure 4e 90 days Liver SMN.tif]

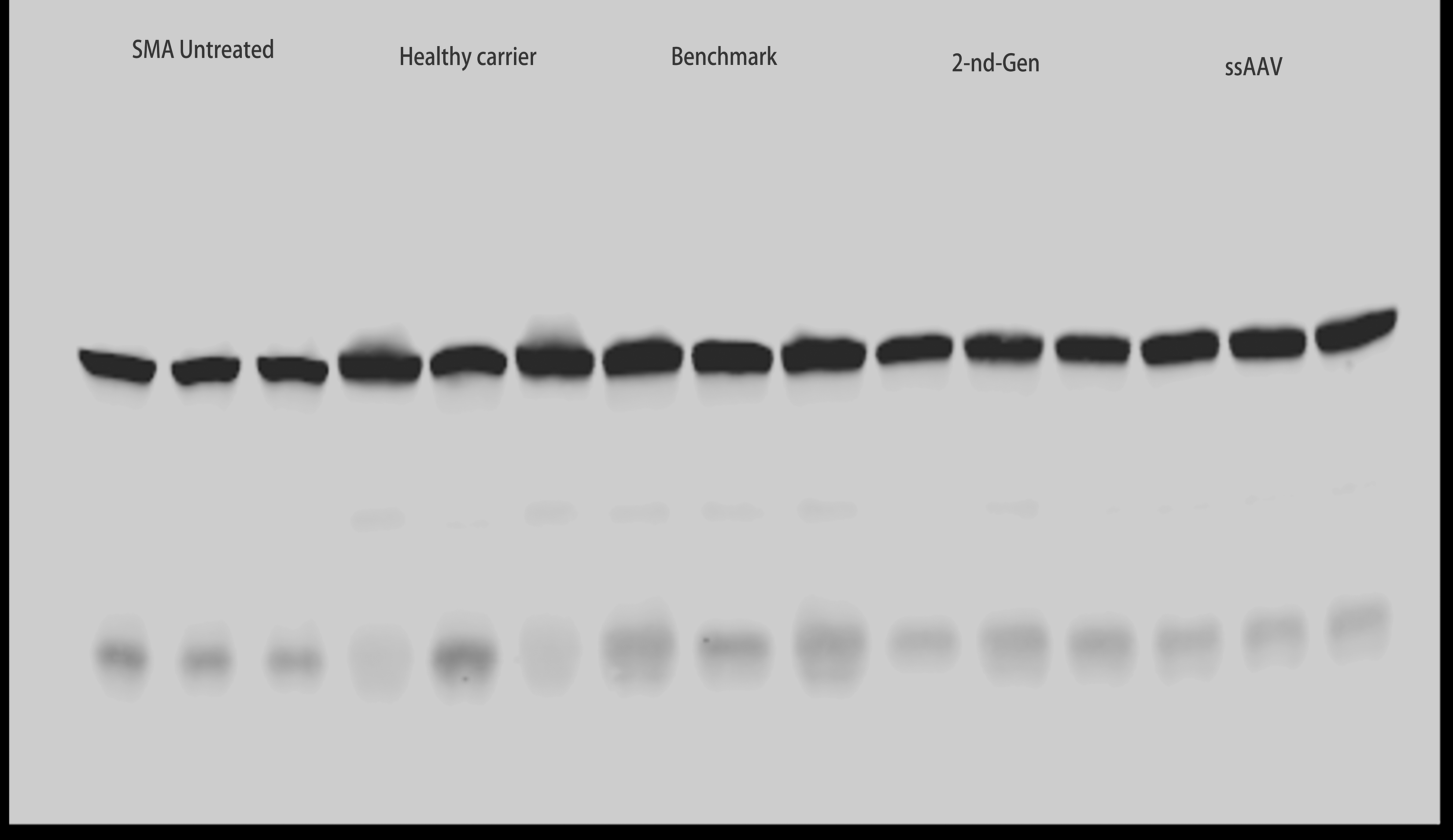

Supplement: Supplementary file 7 — Source Data Fig. 4 [file 44321_2024_37_MOESM7_ESM.zip › Fig 4/Fig4e/Figure 4e 90 days liver Tubulin 2.tif]

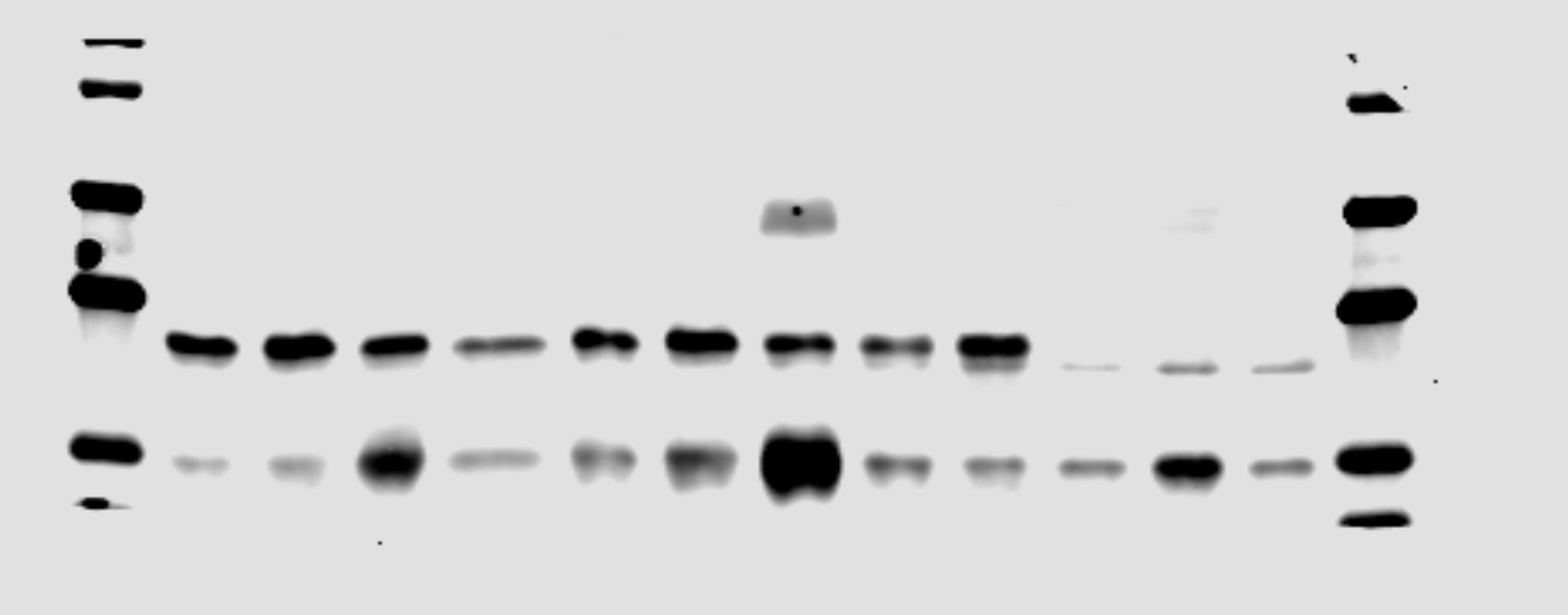

Supplement: Supplementary file 7 — Source Data Fig. 4 [file 44321_2024_37_MOESM7_ESM.zip › Fig 4/Fig4e/figure 4e day30 SMN.tif]

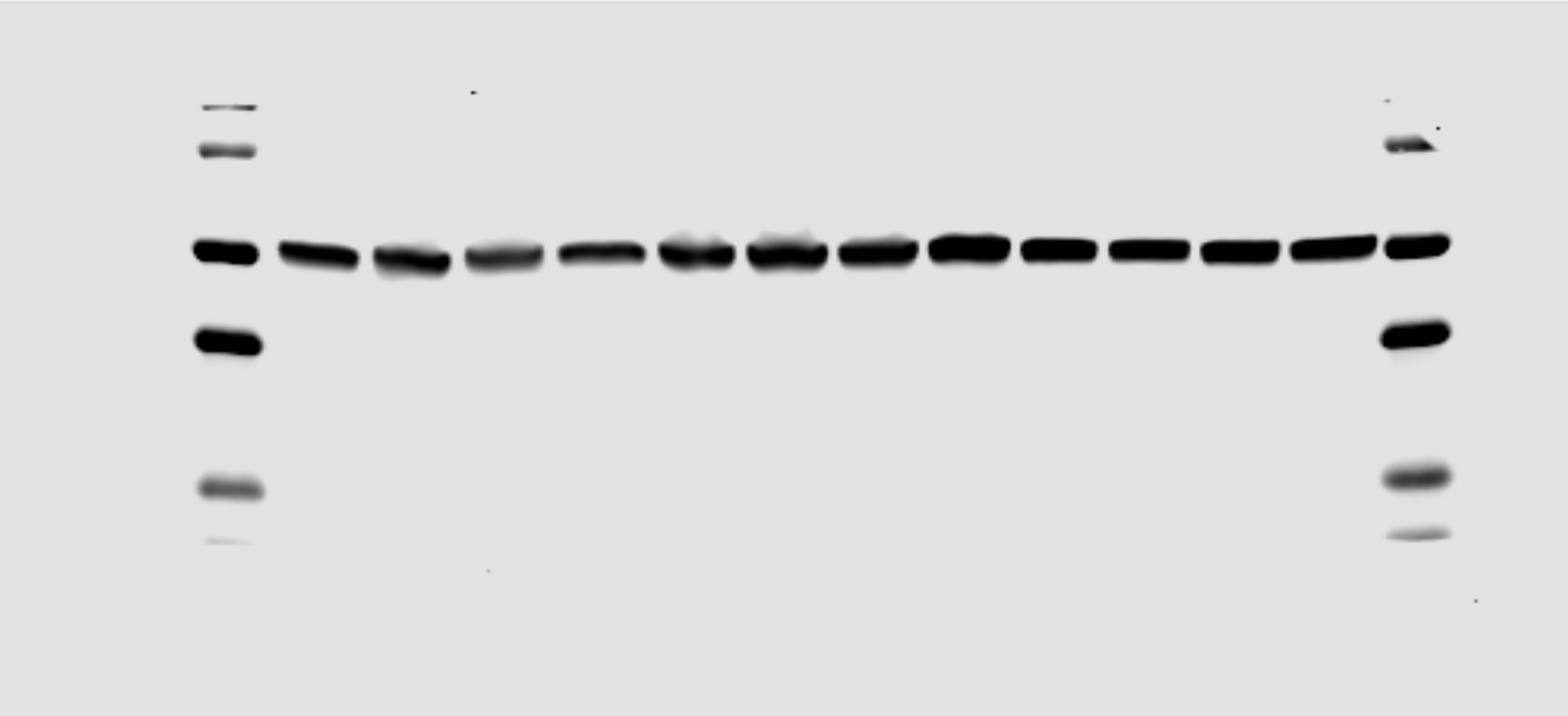

Supplement: Supplementary file 7 — Source Data Fig. 4 [file 44321_2024_37_MOESM7_ESM.zip › Fig 4/Fig4e/figure 4e day30 tubulin.tif]

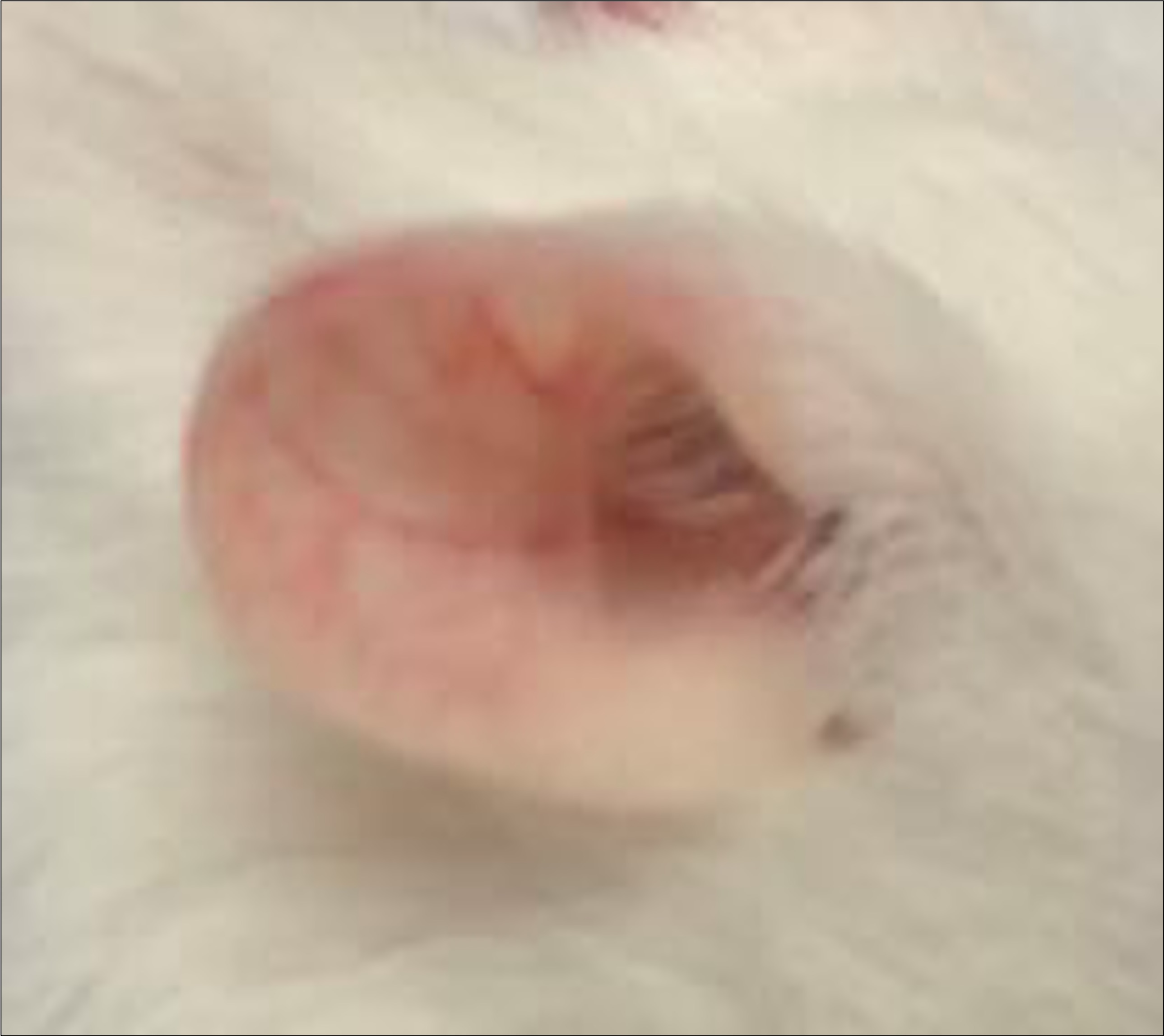

Supplement: Supplementary file 8 — Source Data Fig. 5 [file 44321_2024_37_MOESM8_ESM.zip › Fig 5/Fig5a/Figure 5a 2nd-Gen.tif]

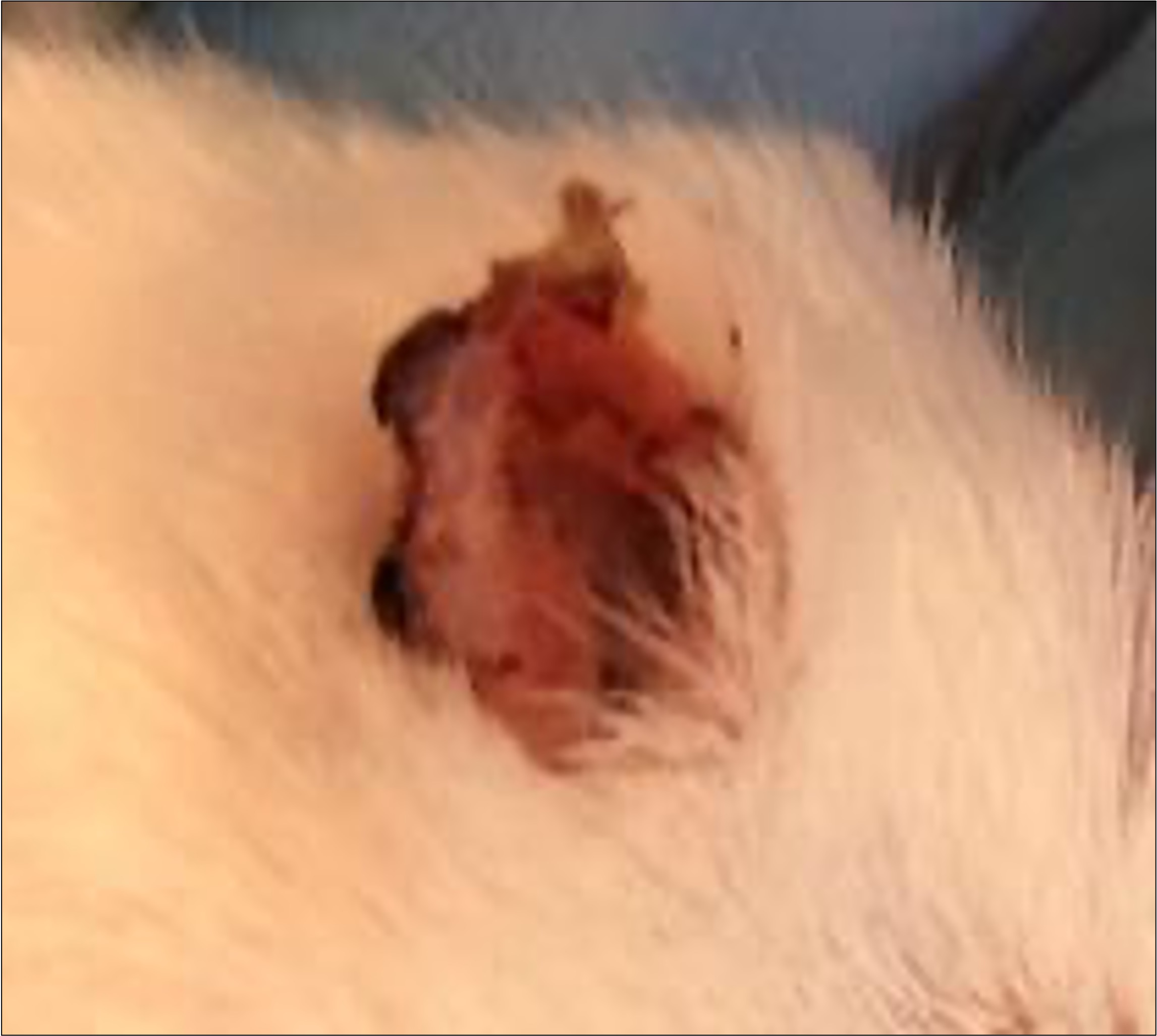

Supplement: Supplementary file 8 — Source Data Fig. 5 [file 44321_2024_37_MOESM8_ESM.zip › Fig 5/Fig5a/Figure 5a Benchmark.tif]

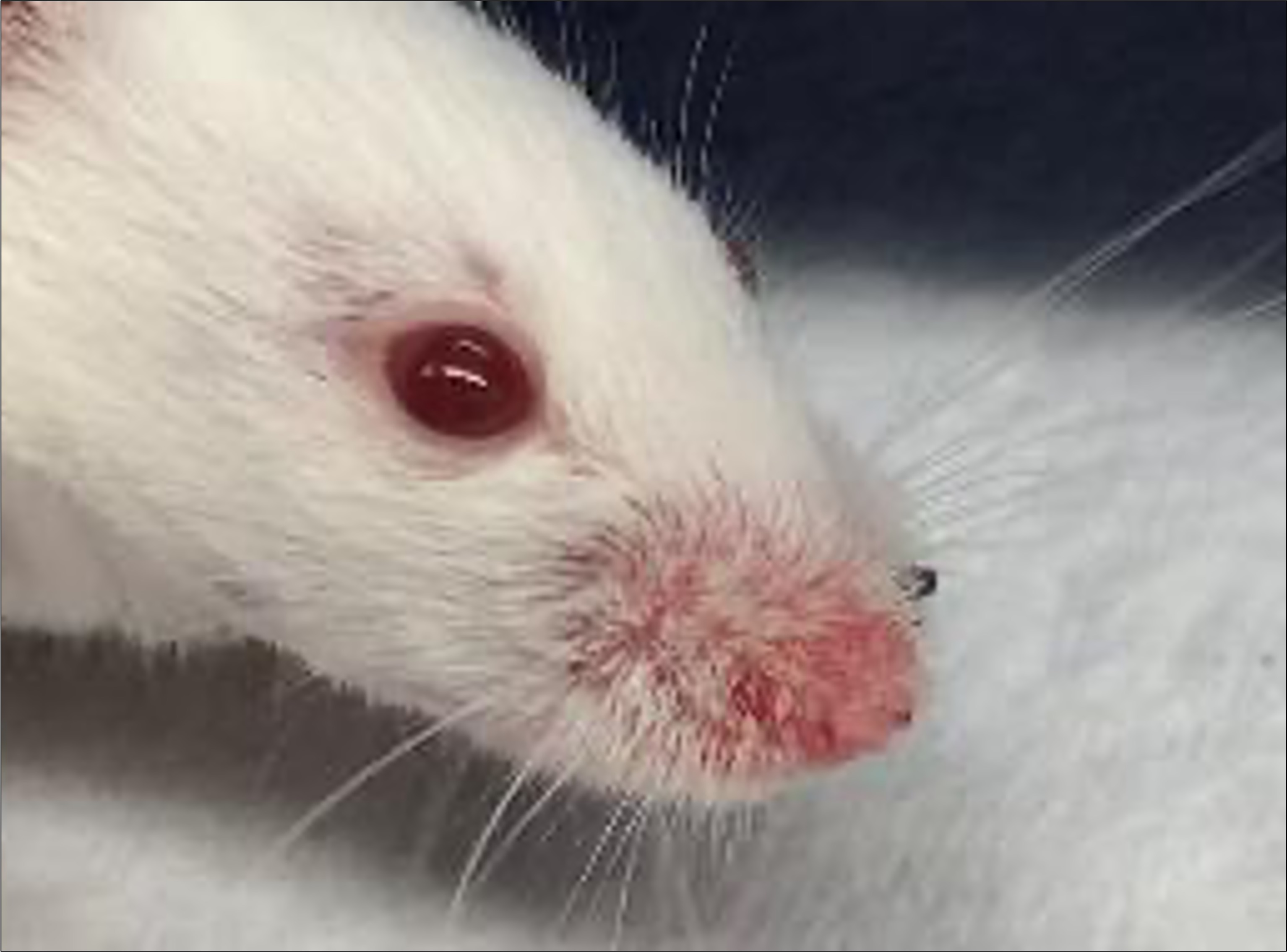

Supplement: Supplementary file 8 — Source Data Fig. 5 [file 44321_2024_37_MOESM8_ESM.zip › Fig 5/Fig5b/Figure 5b 2nd-Gen.tif]

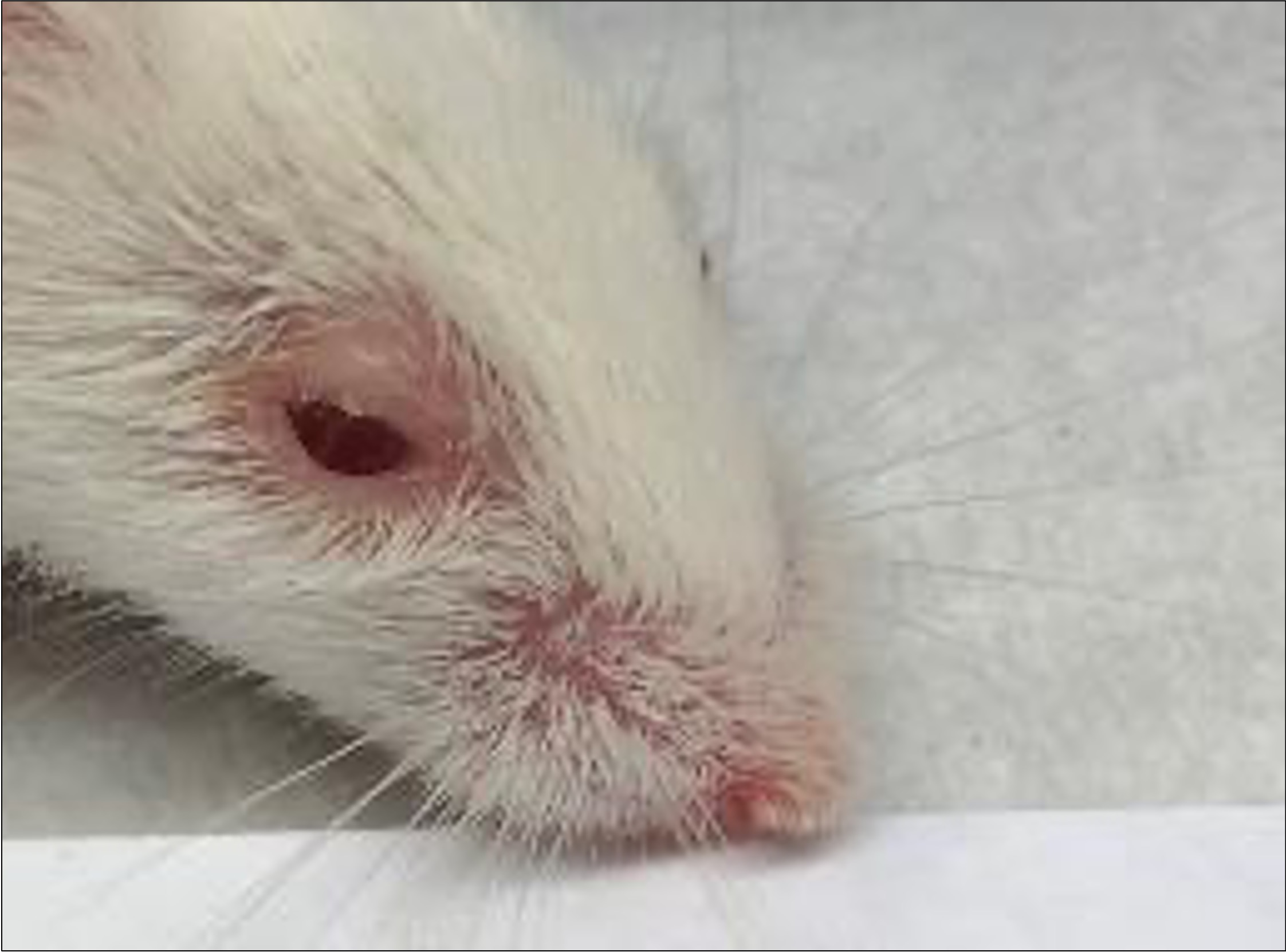

Supplement: Supplementary file 8 — Source Data Fig. 5 [file 44321_2024_37_MOESM8_ESM.zip › Fig 5/Fig5b/Figure 5b Benchmark.tif]

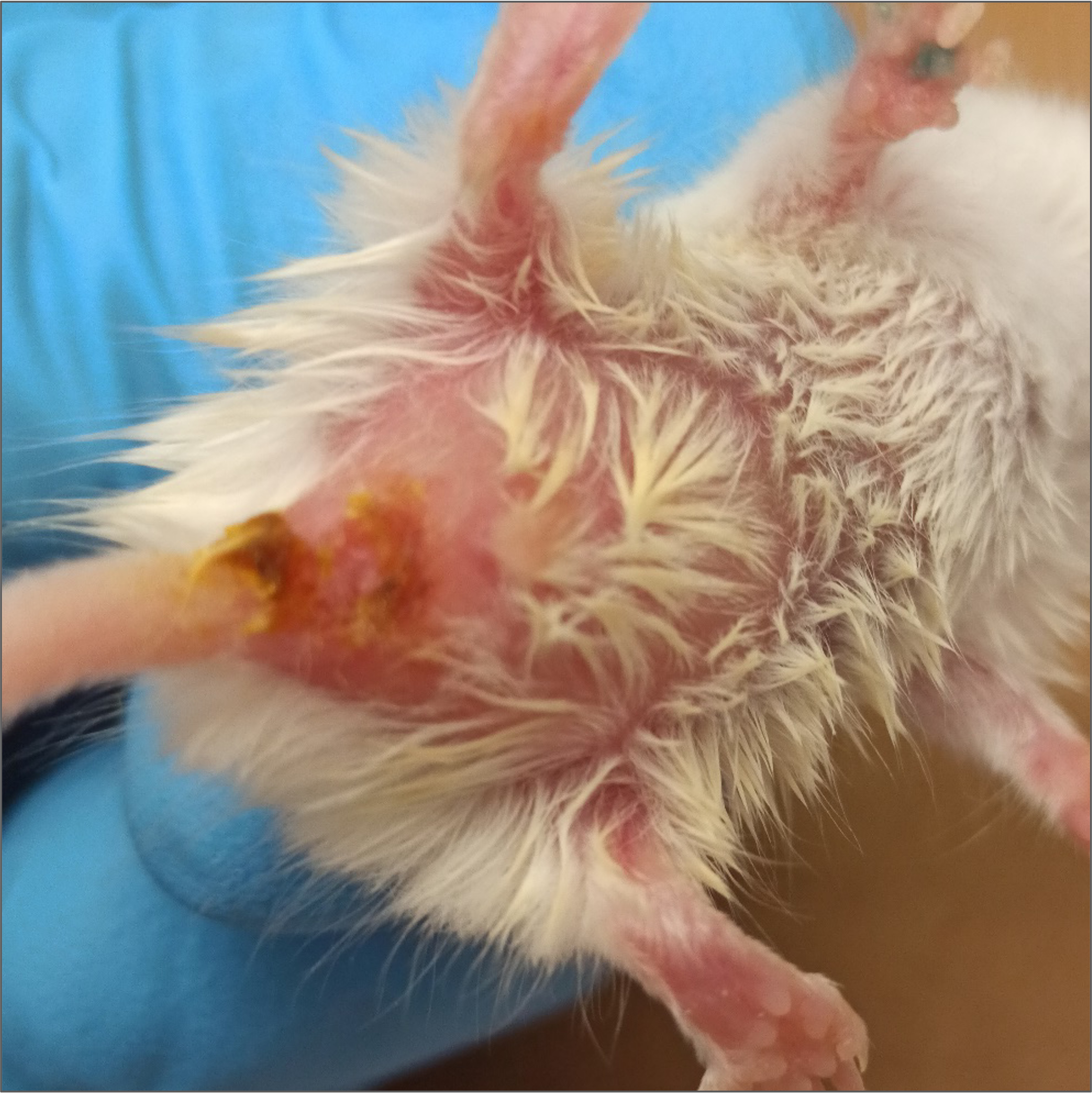

Supplement: Supplementary file 8 — Source Data Fig. 5 [file 44321_2024_37_MOESM8_ESM.zip › Fig 5/Fig5c/Figure 5c Benchmark.tif]

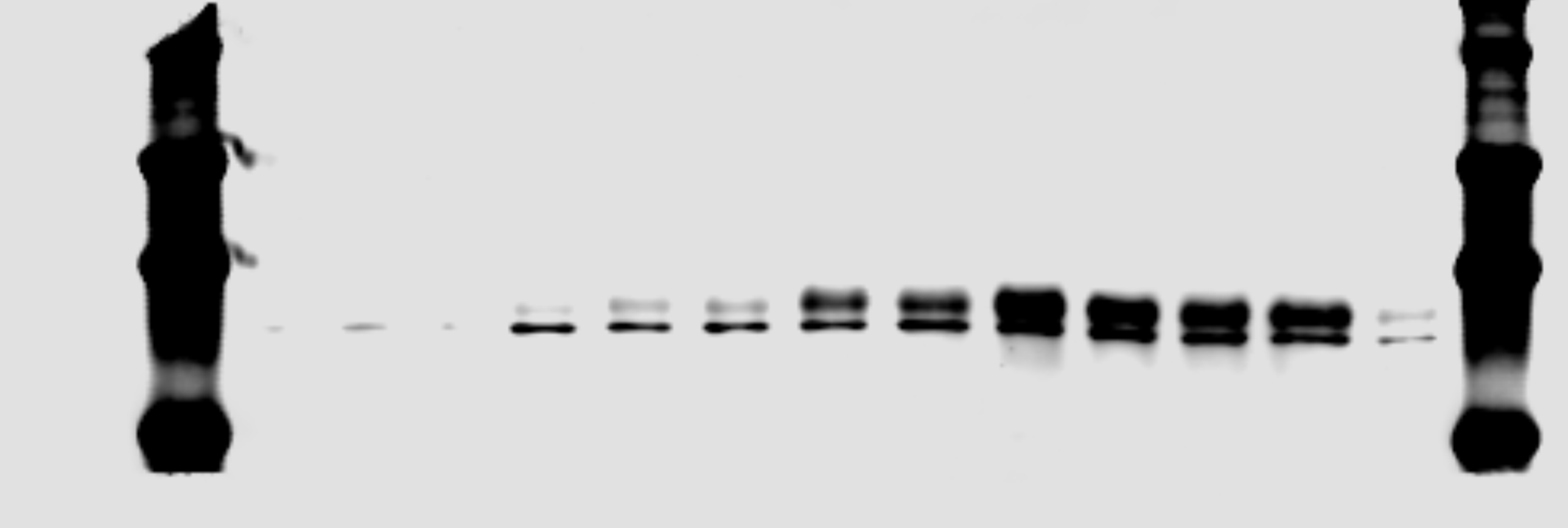

Supplement: Supplementary file 9 — Source Data Fig. 6 [file 44321_2024_37_MOESM9_ESM.zip › Fig 6/Fig6a/figure 6a brain SMN.tif]

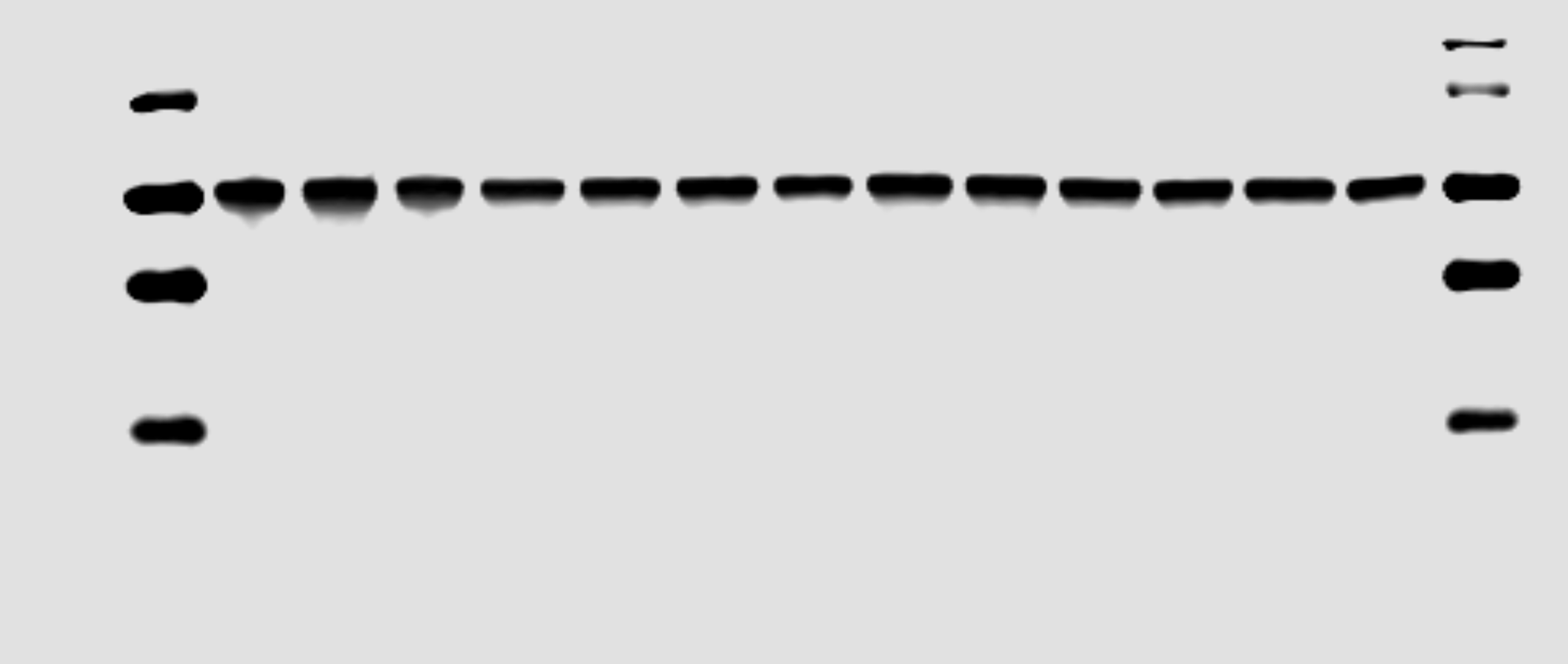

Supplement: Supplementary file 9 — Source Data Fig. 6 [file 44321_2024_37_MOESM9_ESM.zip › Fig 6/Fig6a/figure 6a brain tubulin.tif]

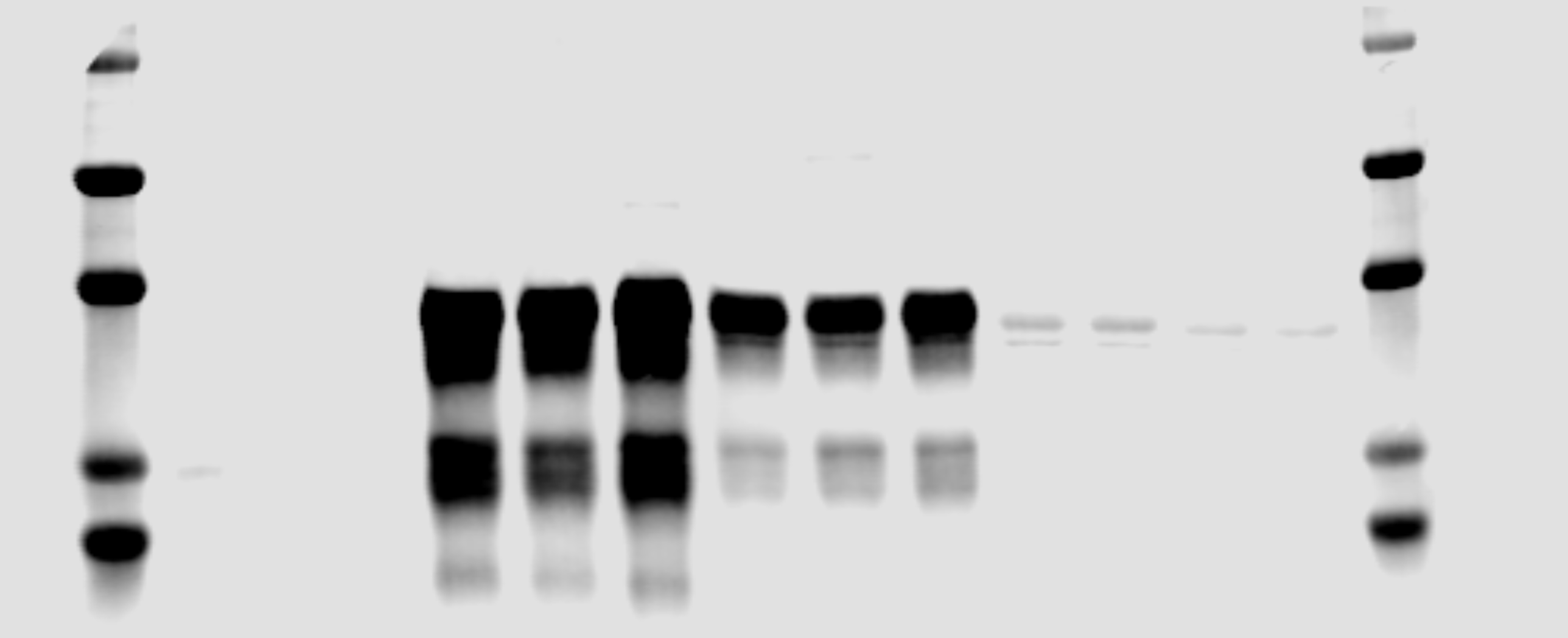

Supplement: Supplementary file 9 — Source Data Fig. 6 [file 44321_2024_37_MOESM9_ESM.zip › Fig 6/Fig6a/figure 6a heart SMN.tif]

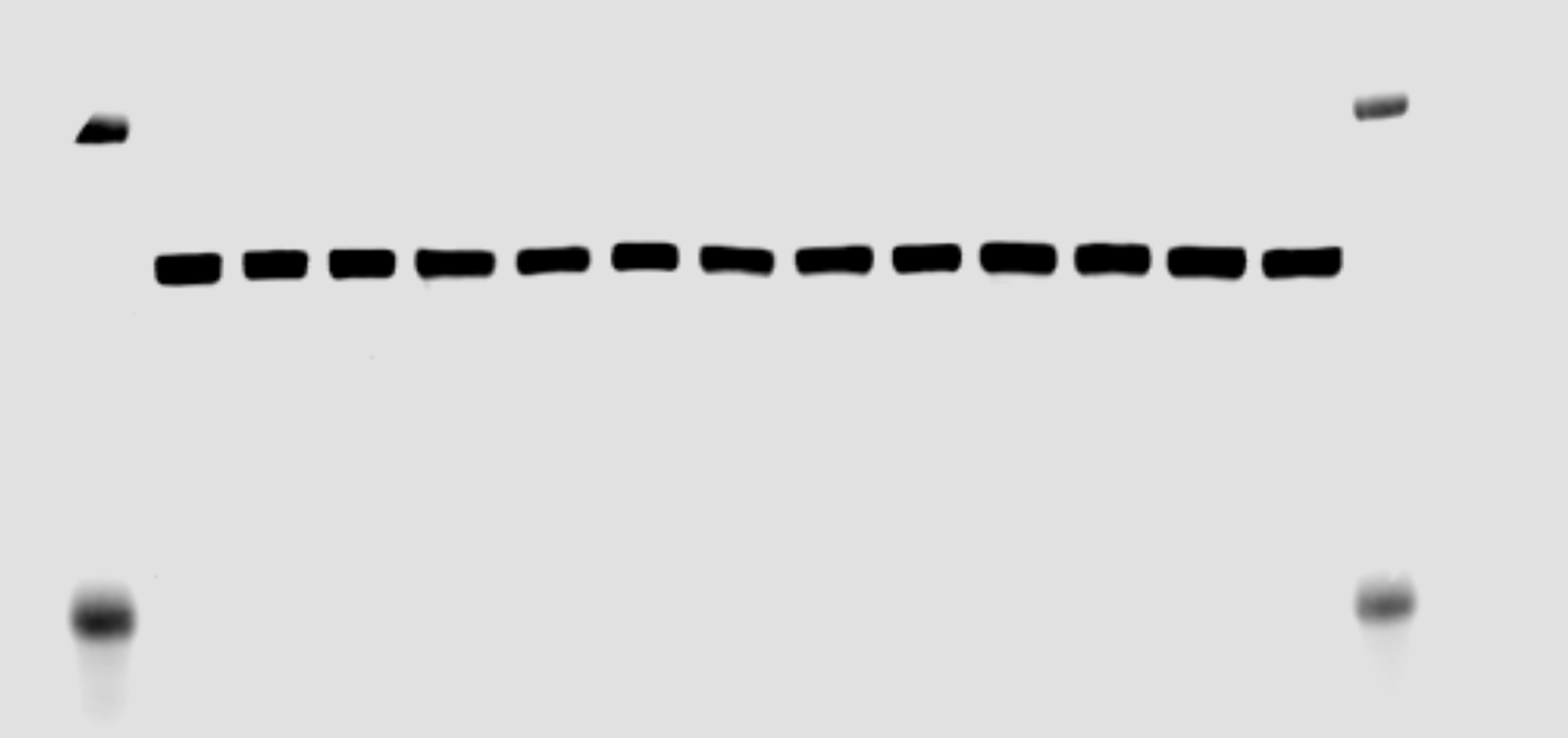

Supplement: Supplementary file 9 — Source Data Fig. 6 [file 44321_2024_37_MOESM9_ESM.zip › Fig 6/Fig6a/figure 6a heart tubulin.tif]

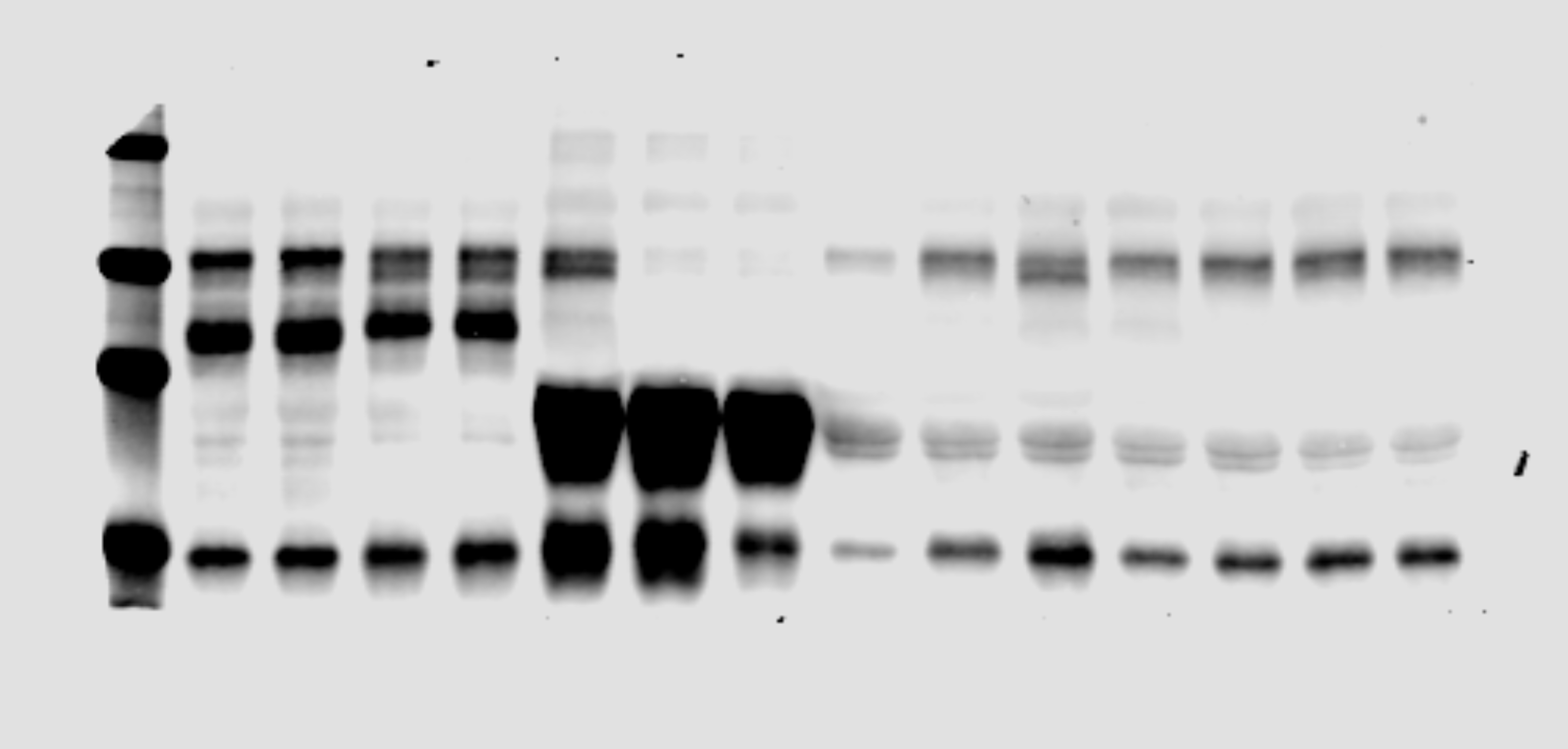

Supplement: Supplementary file 9 — Source Data Fig. 6 [file 44321_2024_37_MOESM9_ESM.zip › Fig 6/Fig6a/figure 6a quadriceps SMN.tif]

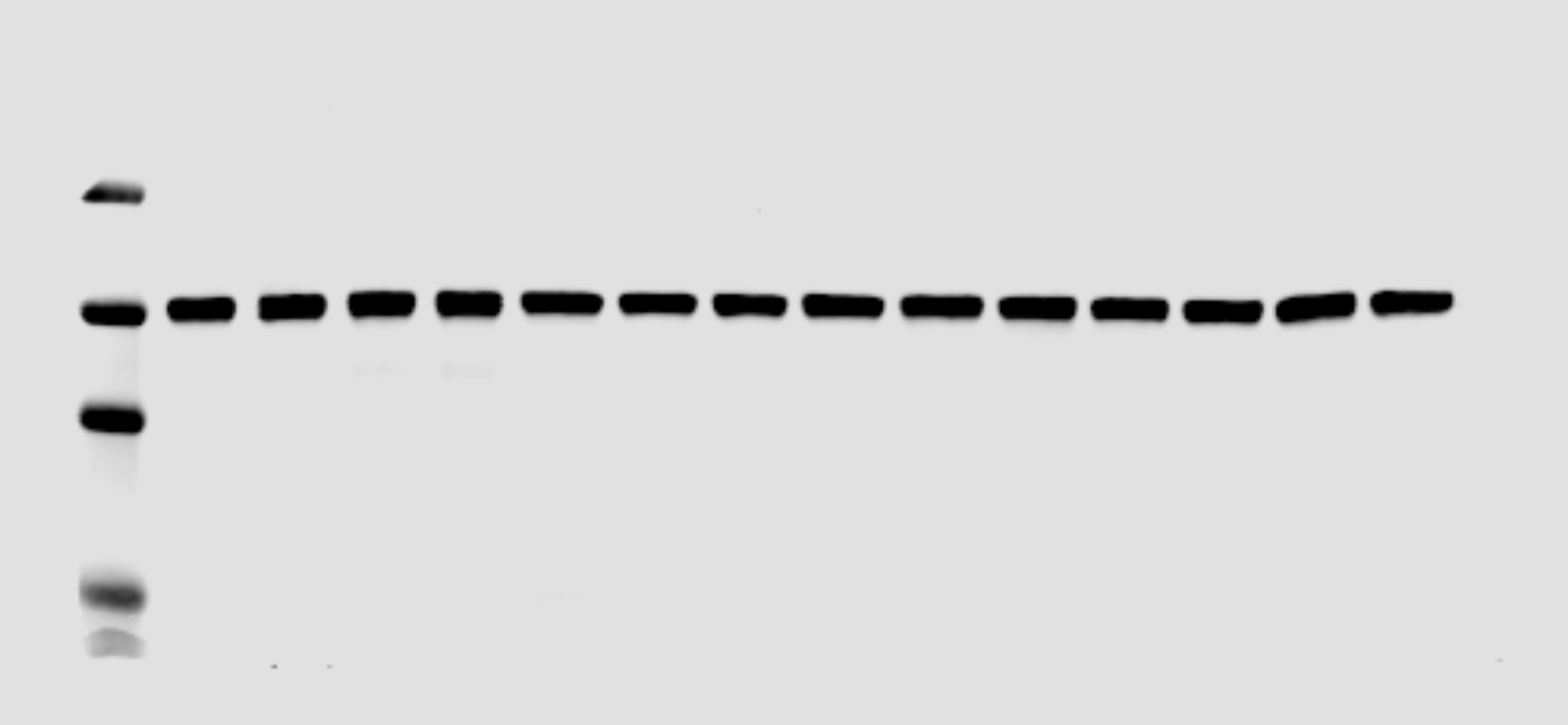

Supplement: Supplementary file 9 — Source Data Fig. 6 [file 44321_2024_37_MOESM9_ESM.zip › Fig 6/Fig6a/figure 6a quadriceps tubulin.tif]

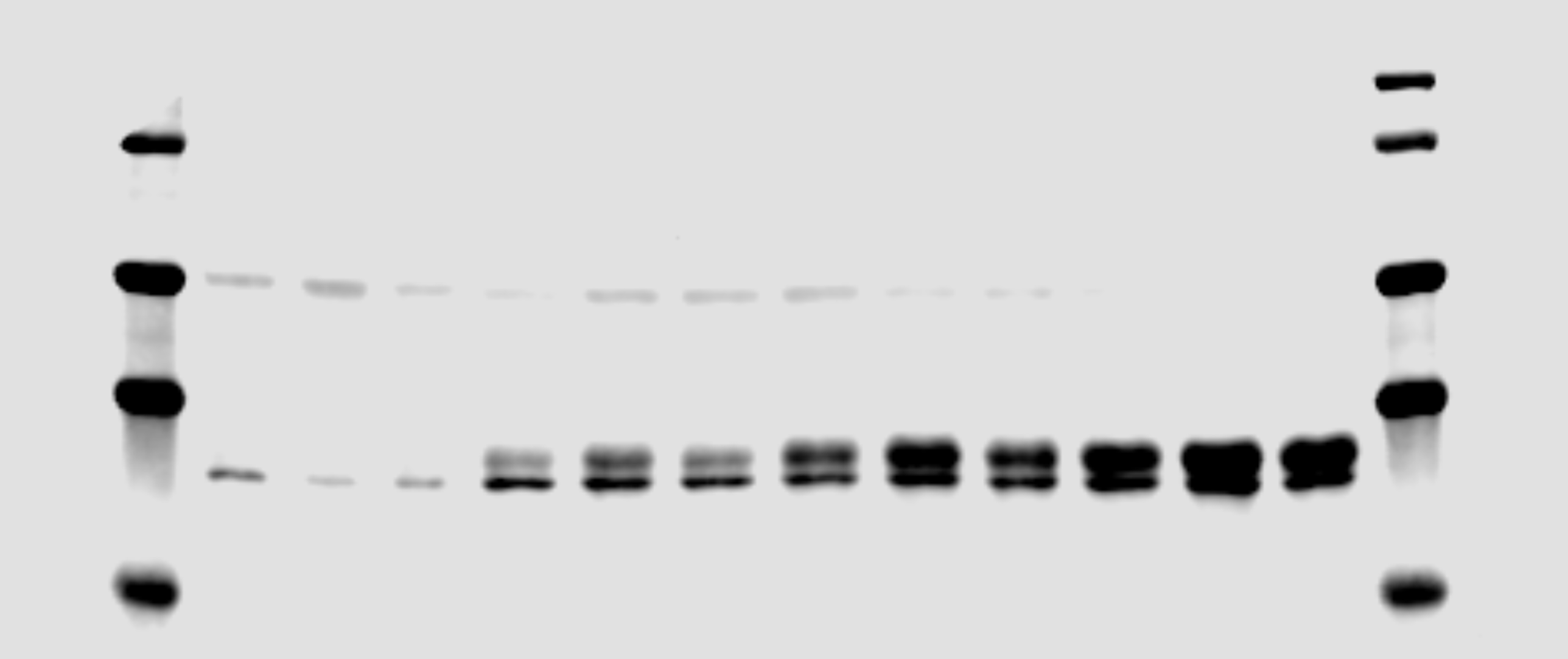

Supplement: Supplementary file 9 — Source Data Fig. 6 [file 44321_2024_37_MOESM9_ESM.zip › Fig 6/Fig6a/figure 6a spinal cord SMN.tif]

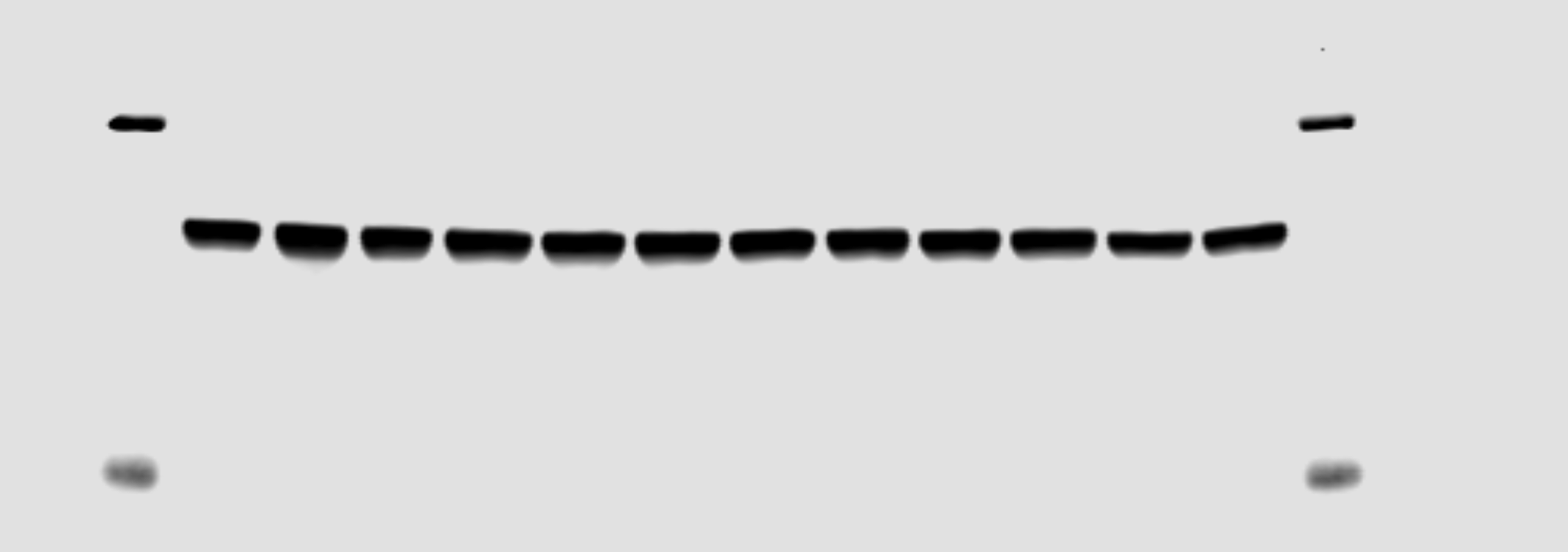

Supplement: Supplementary file 9 — Source Data Fig. 6 [file 44321_2024_37_MOESM9_ESM.zip › Fig 6/Fig6a/figure 6a spinal tubulin.tif]

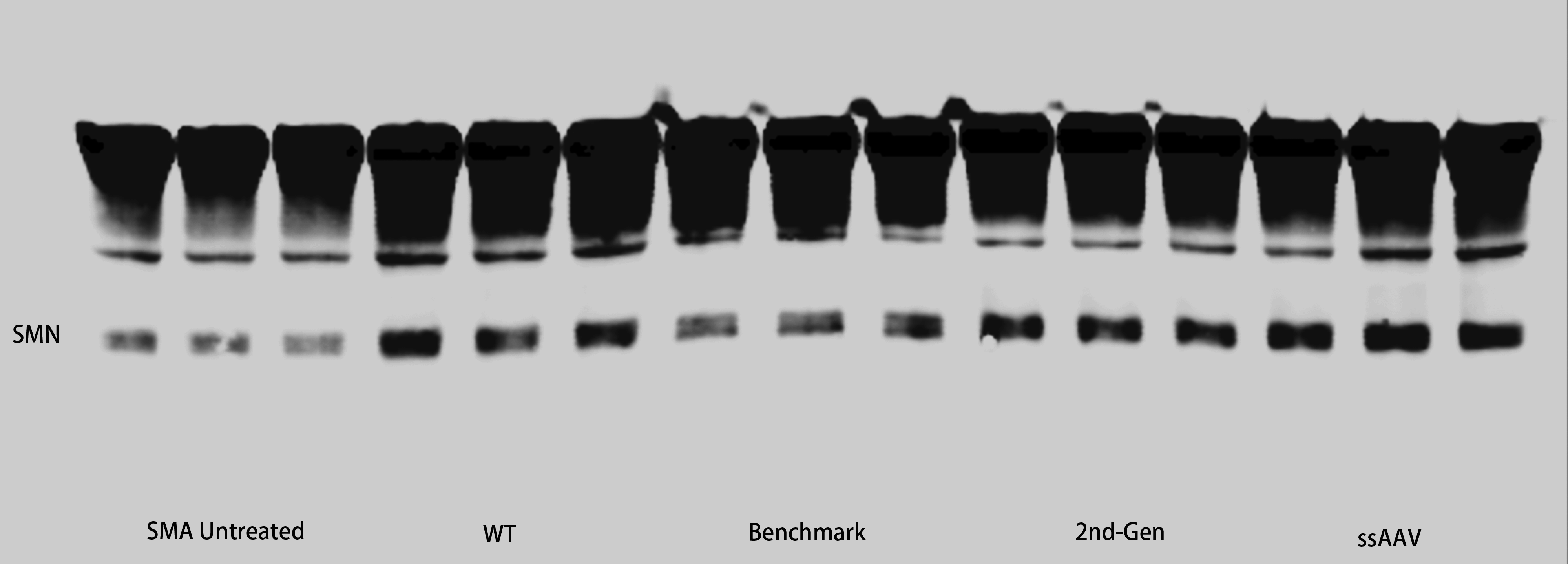

Supplement: Supplementary file 9 — Source Data Fig. 6 [file 44321_2024_37_MOESM9_ESM.zip › Fig 6/Fig6b/Figure 6b brain SMN.tif]

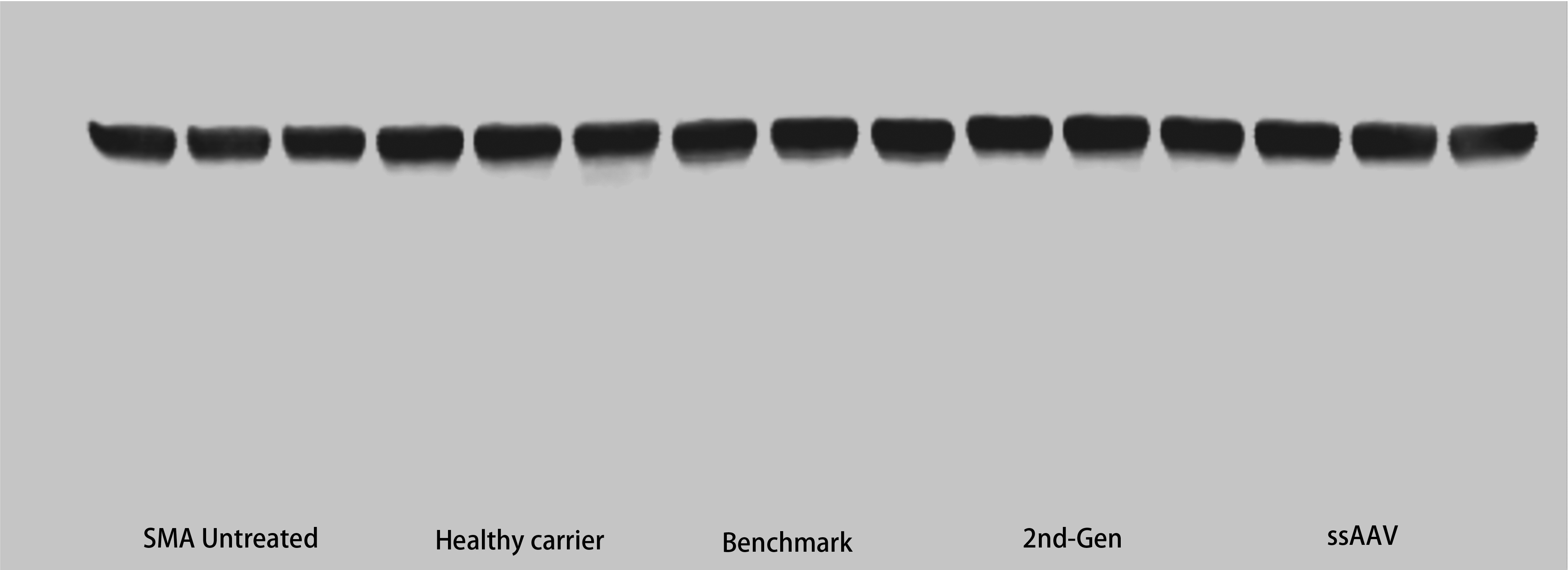

Supplement: Supplementary file 9 — Source Data Fig. 6 [file 44321_2024_37_MOESM9_ESM.zip › Fig 6/Fig6b/Figure 6b brain Tubulin.tif]

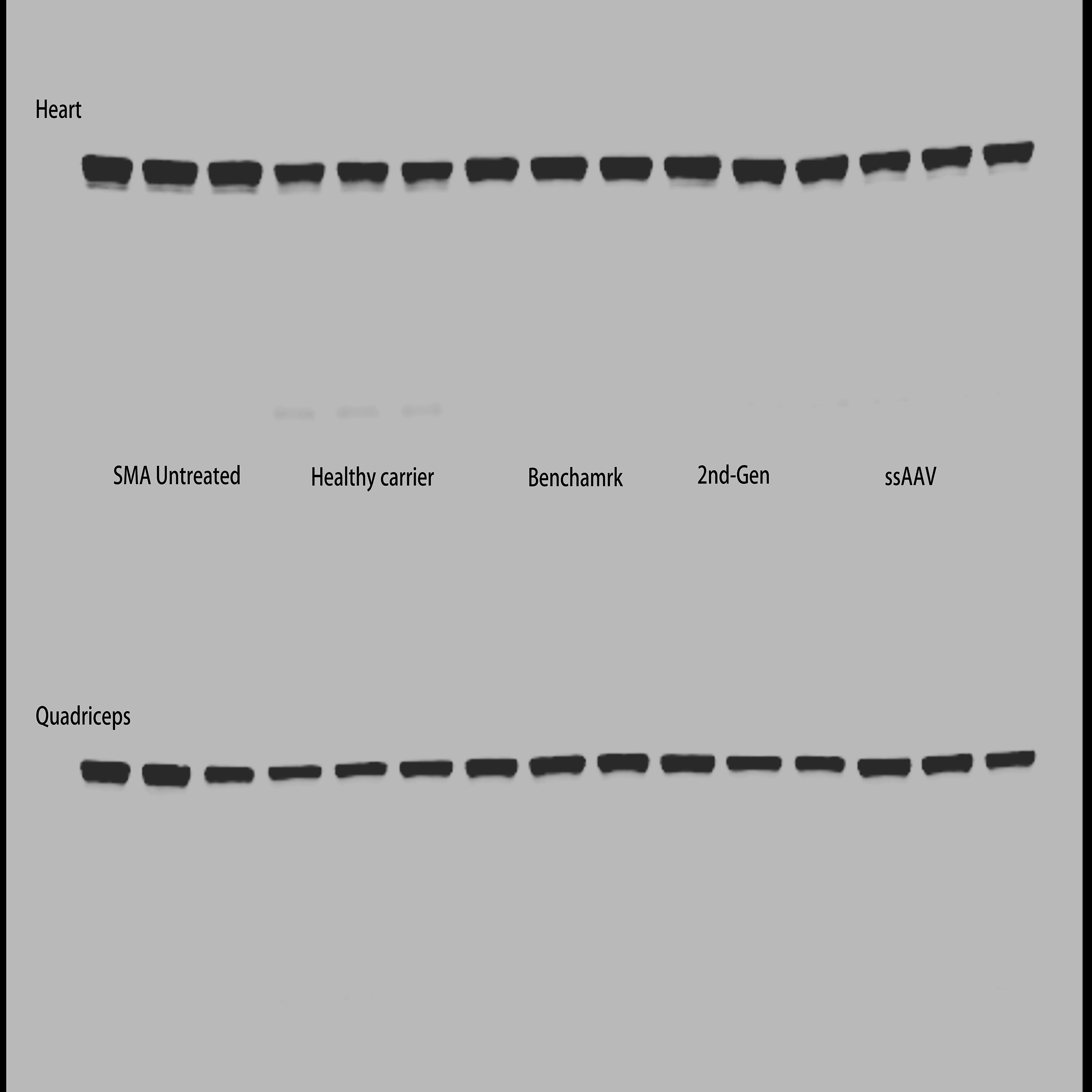

Supplement: Supplementary file 9 — Source Data Fig. 6 [file 44321_2024_37_MOESM9_ESM.zip › Fig 6/Fig6b/Figure 6b heart & quadriceps_Tubulin.tif]

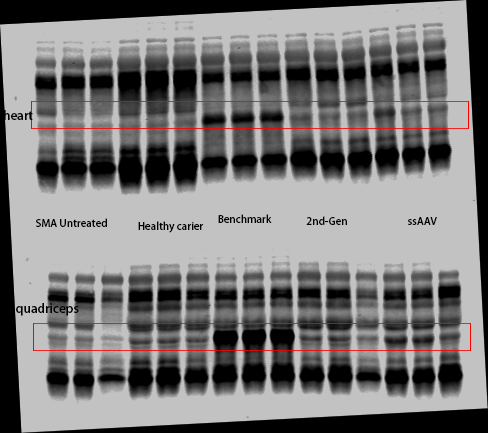

Supplement: Supplementary file 9 — Source Data Fig. 6 [file 44321_2024_37_MOESM9_ESM.zip › Fig 6/Fig6b/Figure 6b heart & quard-SMN.tif]

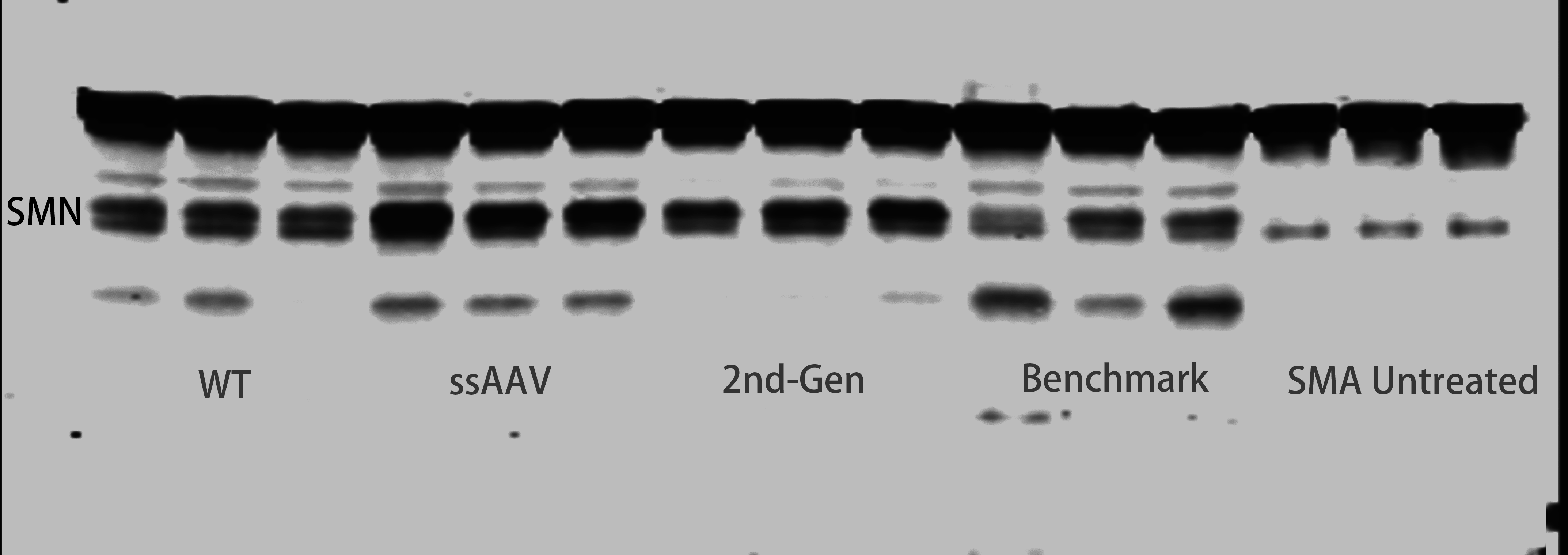

Supplement: Supplementary file 9 — Source Data Fig. 6 [file 44321_2024_37_MOESM9_ESM.zip › Fig 6/Fig6b/Figure 6b spinal cord SMN.tif]

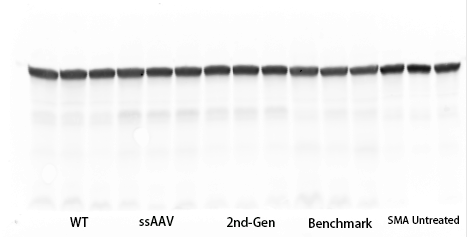

Supplement: Supplementary file 9 — Source Data Fig. 6 [file 44321_2024_37_MOESM9_ESM.zip › Fig 6/Fig6b/Figure 6b spinal cord Tubulin.tif]

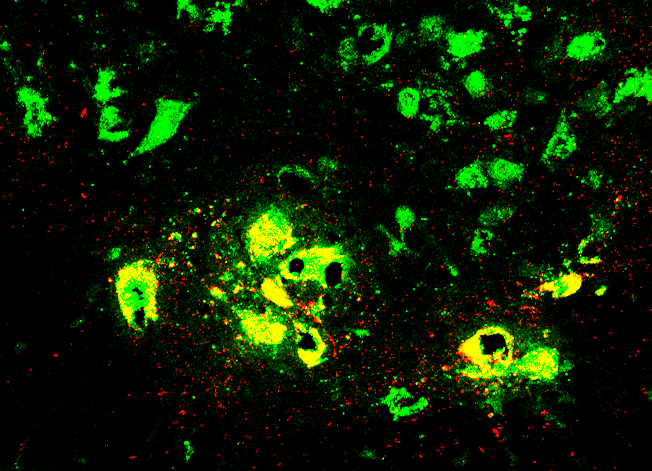

Supplement: Supplementary file 9 — Source Data Fig. 6 [file 44321_2024_37_MOESM9_ESM.zip › Fig 6/Fig6c/2nd Gen ChAT_SMN/Enlarged MD overlay.tif]

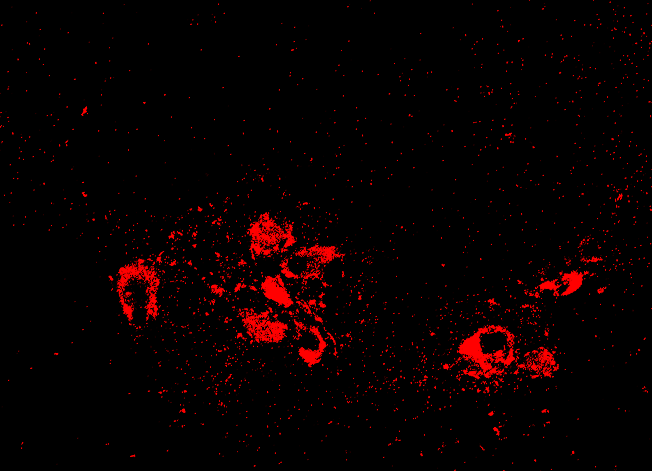

Supplement: Supplementary file 9 — Source Data Fig. 6 [file 44321_2024_37_MOESM9_ESM.zip › Fig 6/Fig6c/2nd Gen ChAT_SMN/Enlarged MD1.tif]

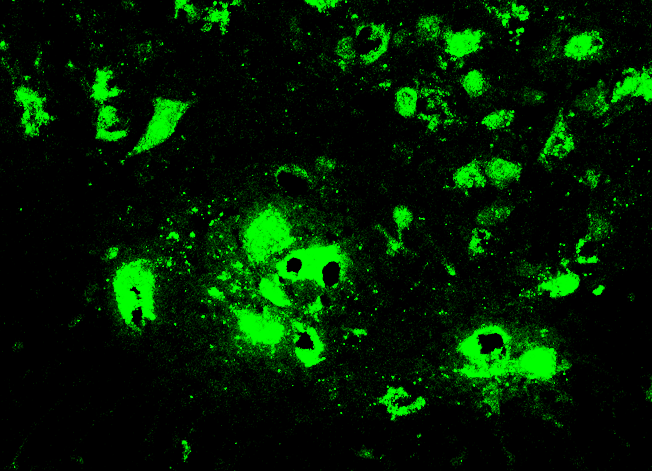

Supplement: Supplementary file 9 — Source Data Fig. 6 [file 44321_2024_37_MOESM9_ESM.zip › Fig 6/Fig6c/2nd Gen ChAT_SMN/Enlarged MD2.tif]

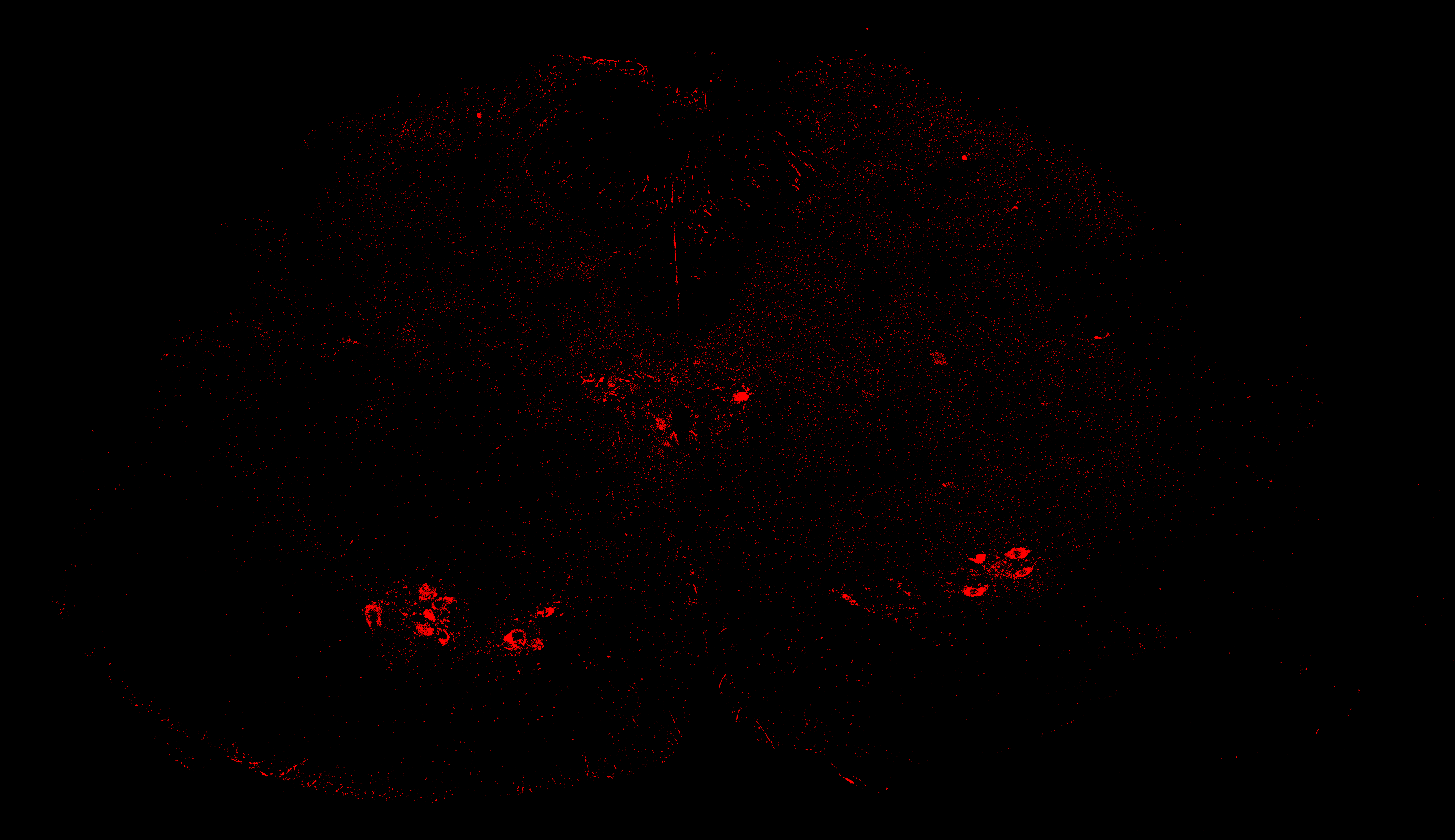

Supplement: Supplementary file 9 — Source Data Fig. 6 [file 44321_2024_37_MOESM9_ESM.zip › Fig 6/Fig6c/2nd Gen ChAT_SMN/MD_1.tif]

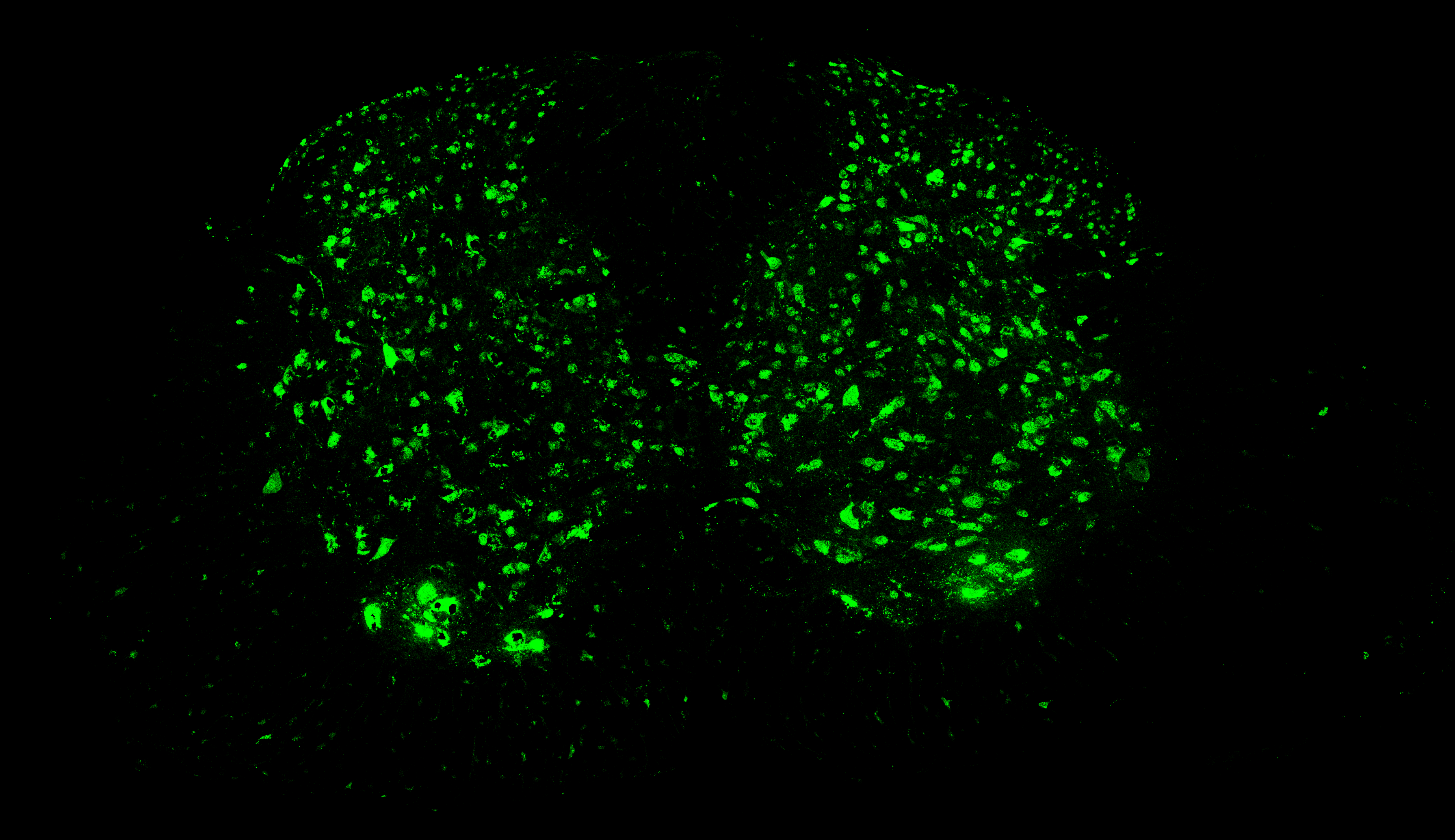

Supplement: Supplementary file 9 — Source Data Fig. 6 [file 44321_2024_37_MOESM9_ESM.zip › Fig 6/Fig6c/2nd Gen ChAT_SMN/MD_2.tif]

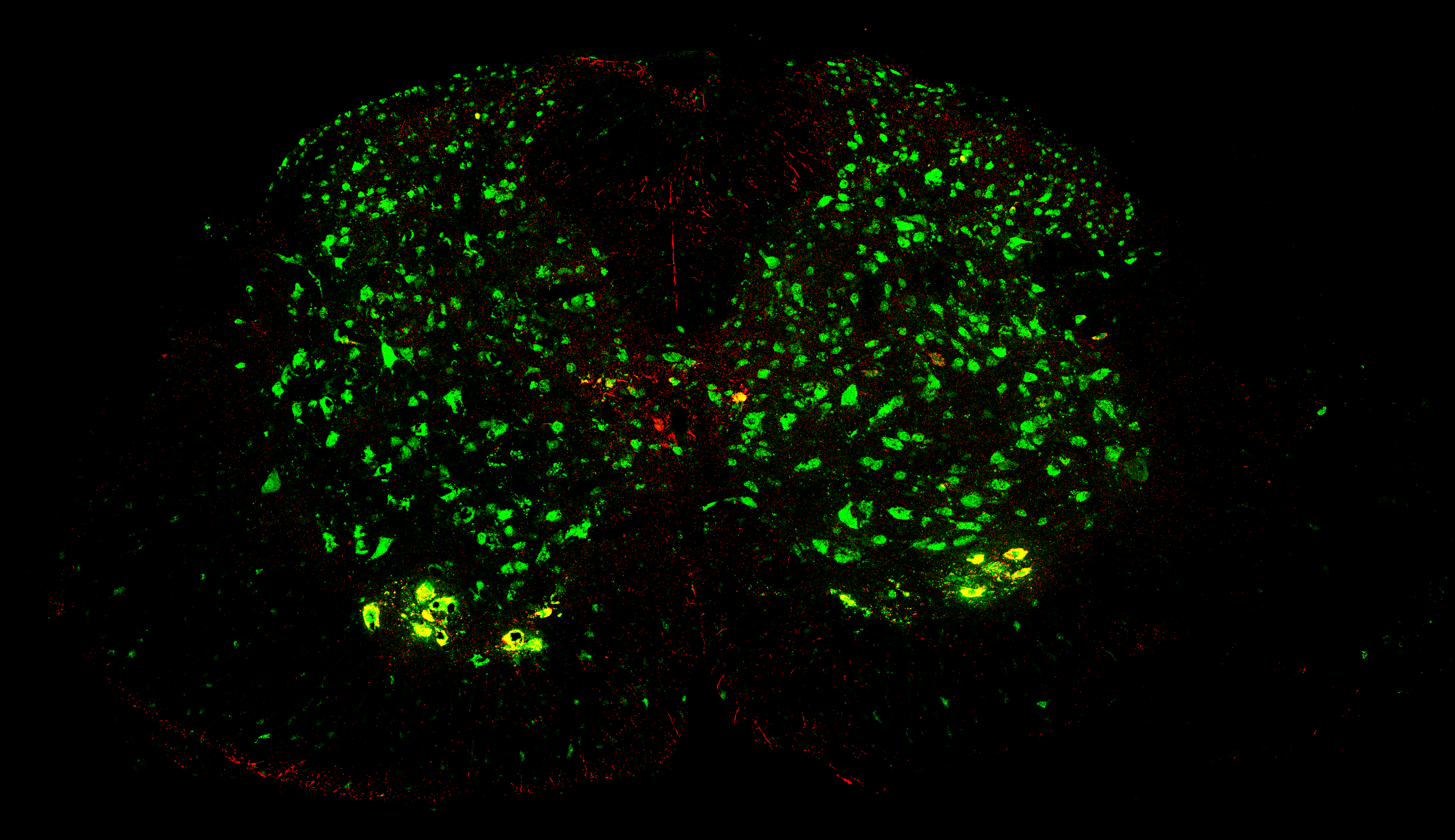

Supplement: Supplementary file 9 — Source Data Fig. 6 [file 44321_2024_37_MOESM9_ESM.zip › Fig 6/Fig6c/2nd Gen ChAT_SMN/MD_overlay.tif]

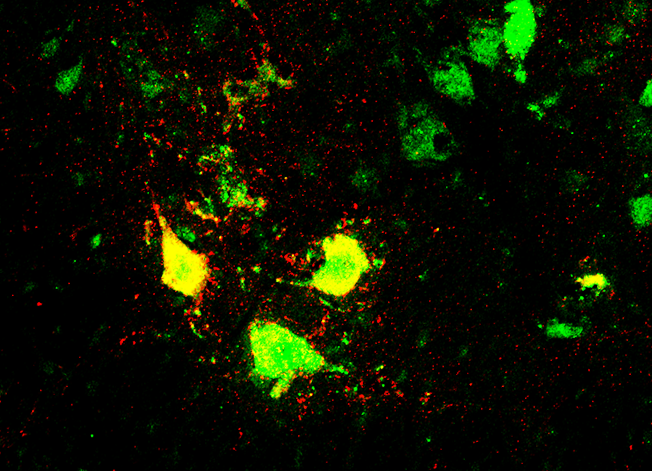

Supplement: Supplementary file 9 — Source Data Fig. 6 [file 44321_2024_37_MOESM9_ESM.zip › Fig 6/Fig6c/Benchmark ChAT_SMN/Enlarged _MD overlay.tif]

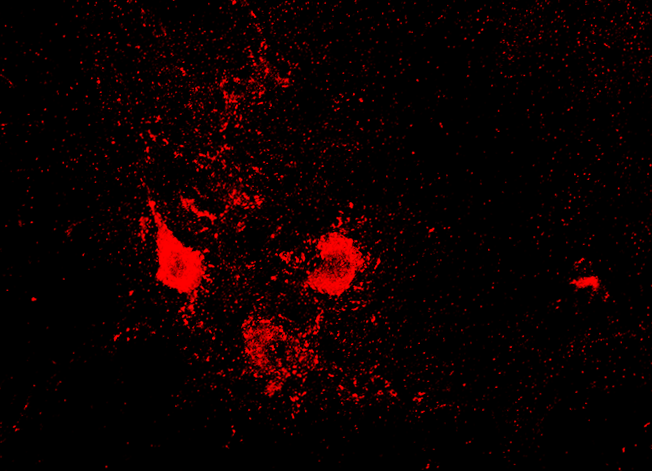

Supplement: Supplementary file 9 — Source Data Fig. 6 [file 44321_2024_37_MOESM9_ESM.zip › Fig 6/Fig6c/Benchmark ChAT_SMN/Enlarged MD_1.tif]

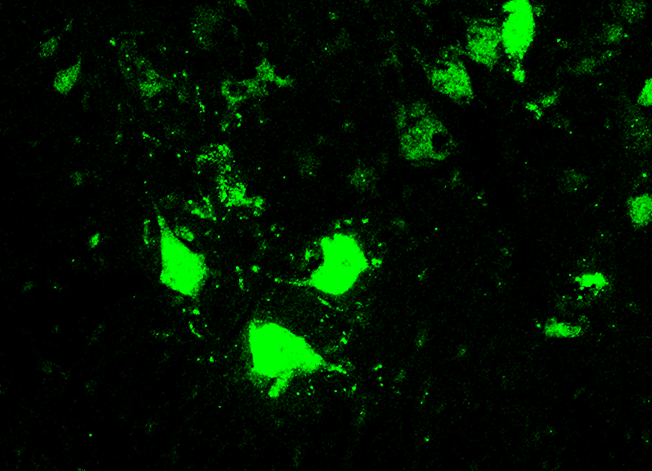

Supplement: Supplementary file 9 — Source Data Fig. 6 [file 44321_2024_37_MOESM9_ESM.zip › Fig 6/Fig6c/Benchmark ChAT_SMN/Enlarged MD_2.tif]

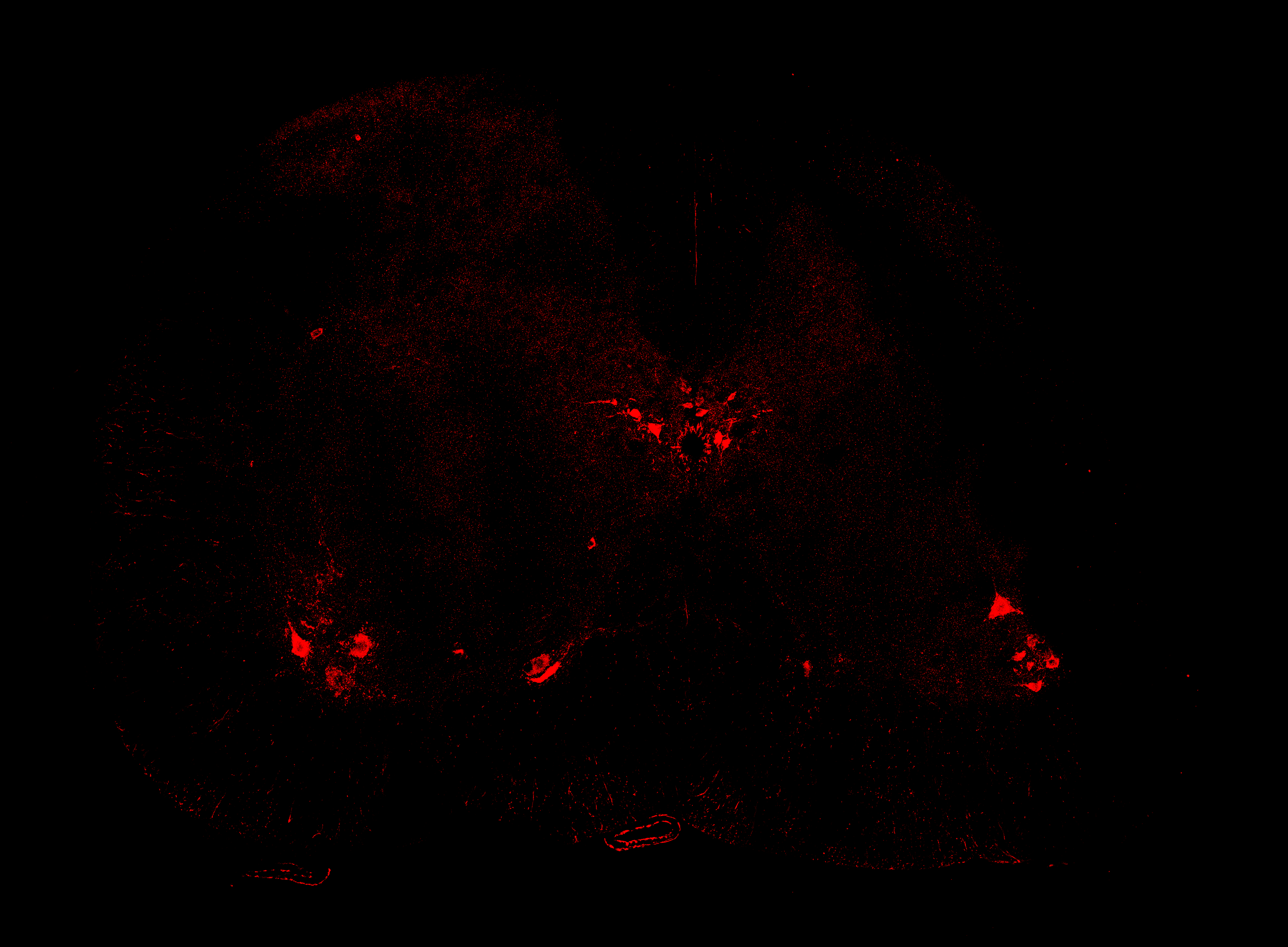

Supplement: Supplementary file 9 — Source Data Fig. 6 [file 44321_2024_37_MOESM9_ESM.zip › Fig 6/Fig6c/Benchmark ChAT_SMN/MD_1.tif]

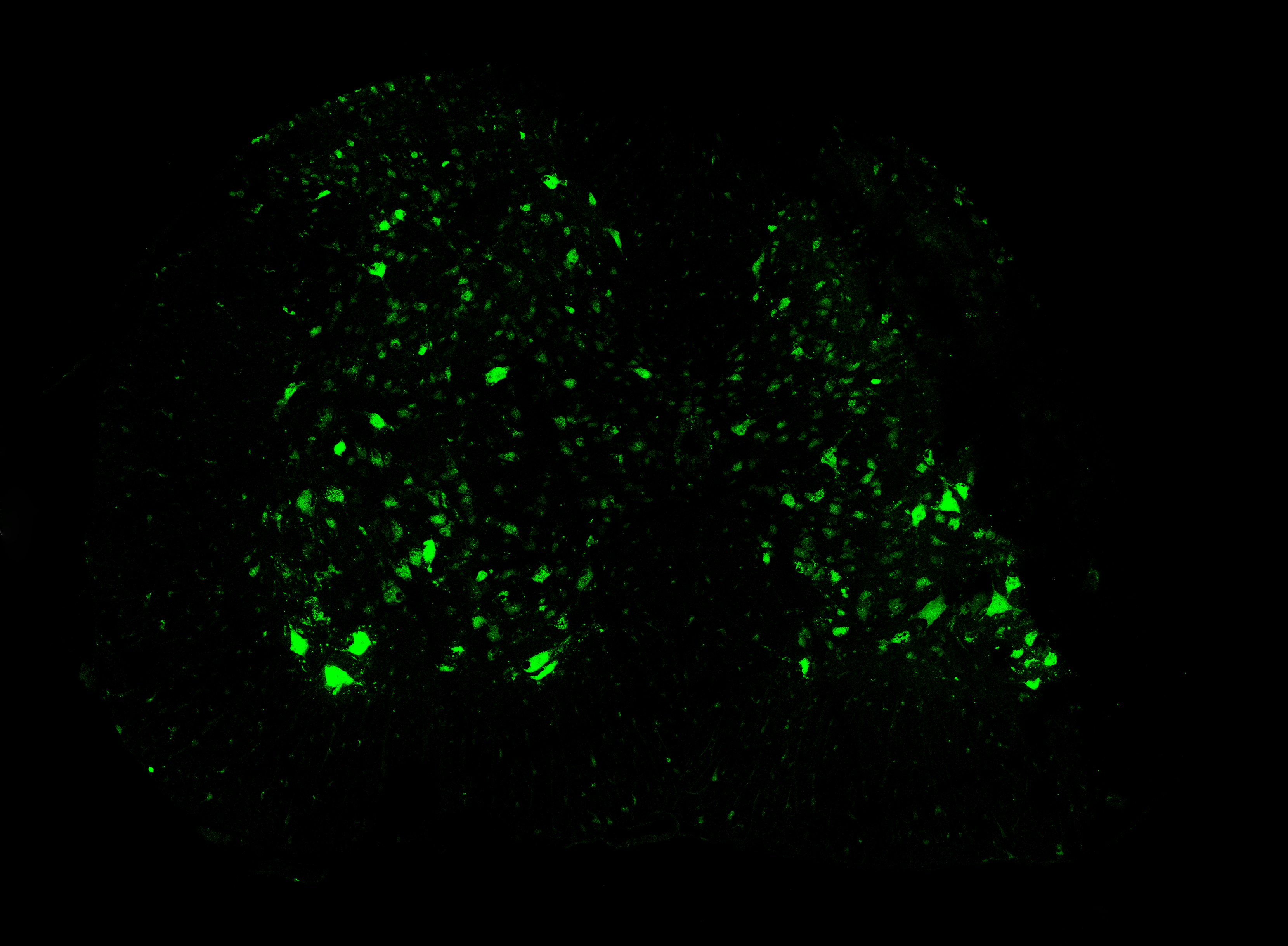

Supplement: Supplementary file 9 — Source Data Fig. 6 [file 44321_2024_37_MOESM9_ESM.zip › Fig 6/Fig6c/Benchmark ChAT_SMN/MD_2.tif]

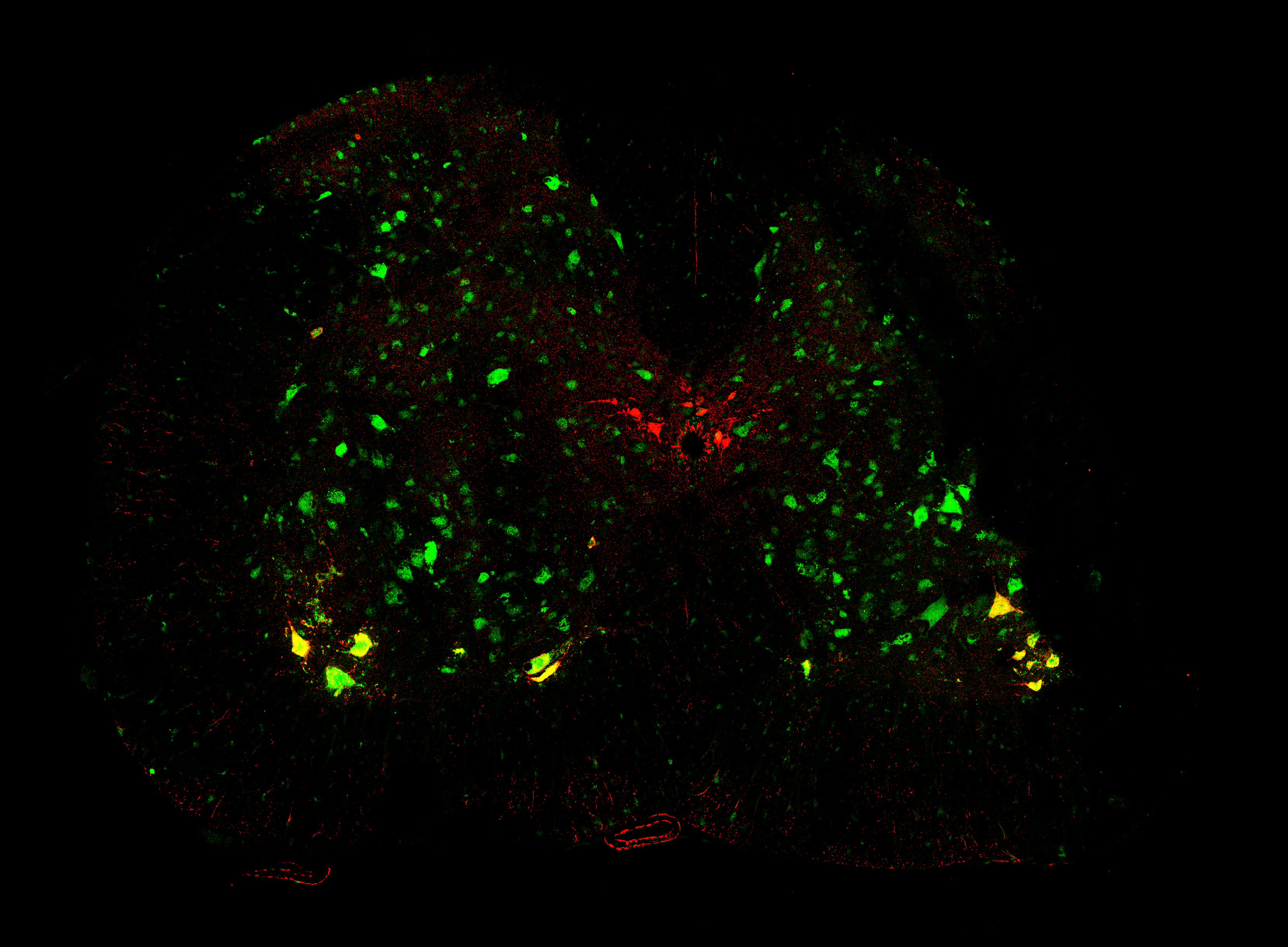

Supplement: Supplementary file 9 — Source Data Fig. 6 [file 44321_2024_37_MOESM9_ESM.zip › Fig 6/Fig6c/Benchmark ChAT_SMN/MD_overlay.tif]

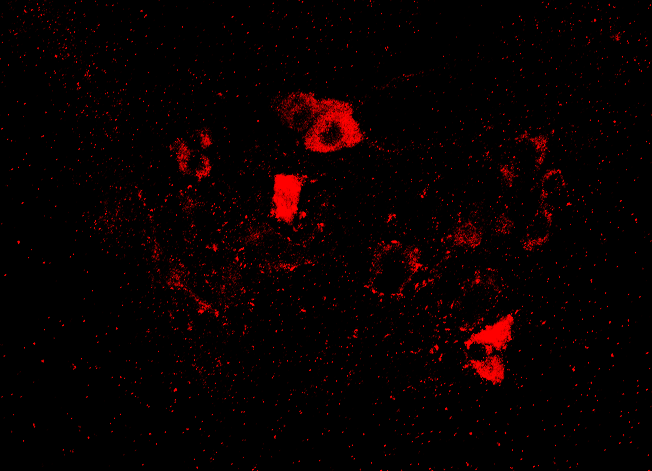

Supplement: Supplementary file 9 — Source Data Fig. 6 [file 44321_2024_37_MOESM9_ESM.zip › Fig 6/Fig6c/Healthy carrier ChAT_SMN/Enlarged MD1.tif]

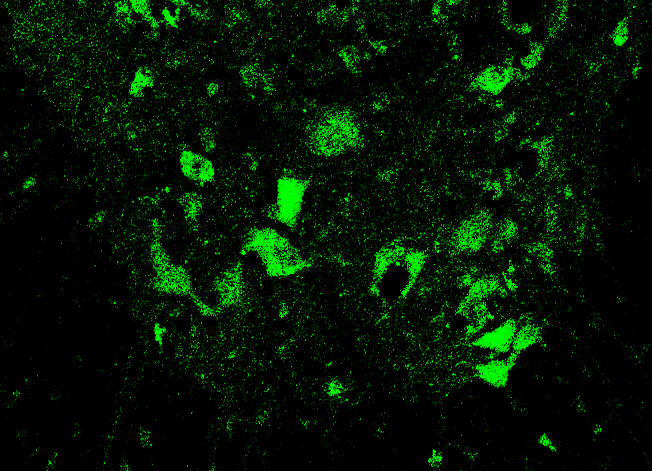

Supplement: Supplementary file 9 — Source Data Fig. 6 [file 44321_2024_37_MOESM9_ESM.zip › Fig 6/Fig6c/Healthy carrier ChAT_SMN/Enlarged MD2.tif]

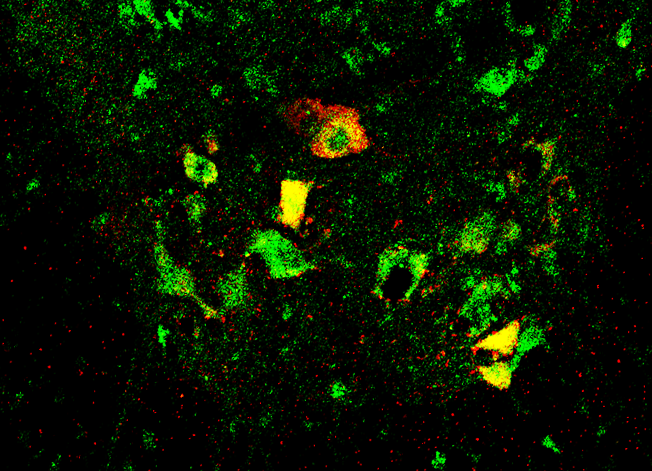

Supplement: Supplementary file 9 — Source Data Fig. 6 [file 44321_2024_37_MOESM9_ESM.zip › Fig 6/Fig6c/Healthy carrier ChAT_SMN/Enlarged MD3.tif]

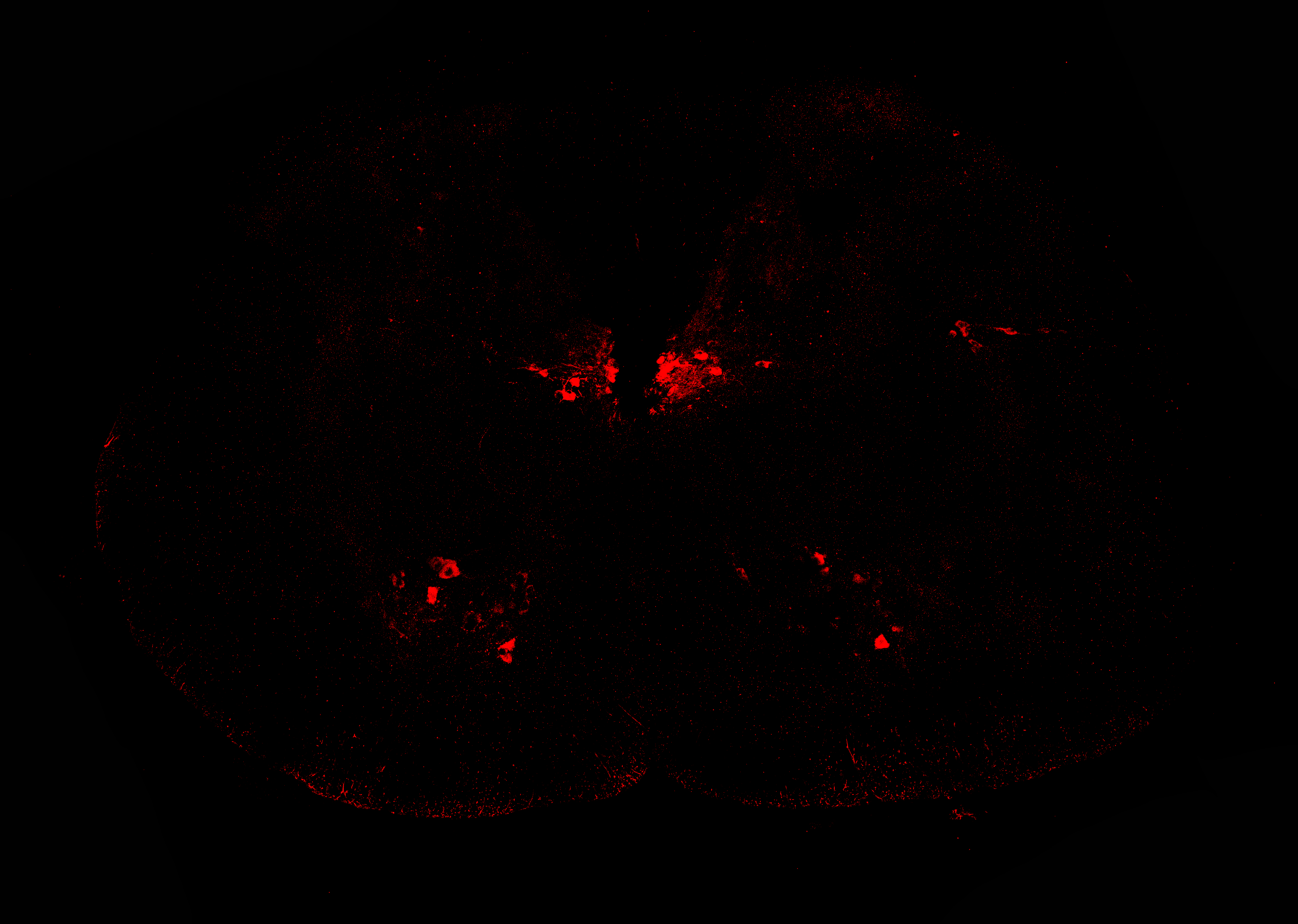

Supplement: Supplementary file 9 — Source Data Fig. 6 [file 44321_2024_37_MOESM9_ESM.zip › Fig 6/Fig6c/Healthy carrier ChAT_SMN/MD1.tif]

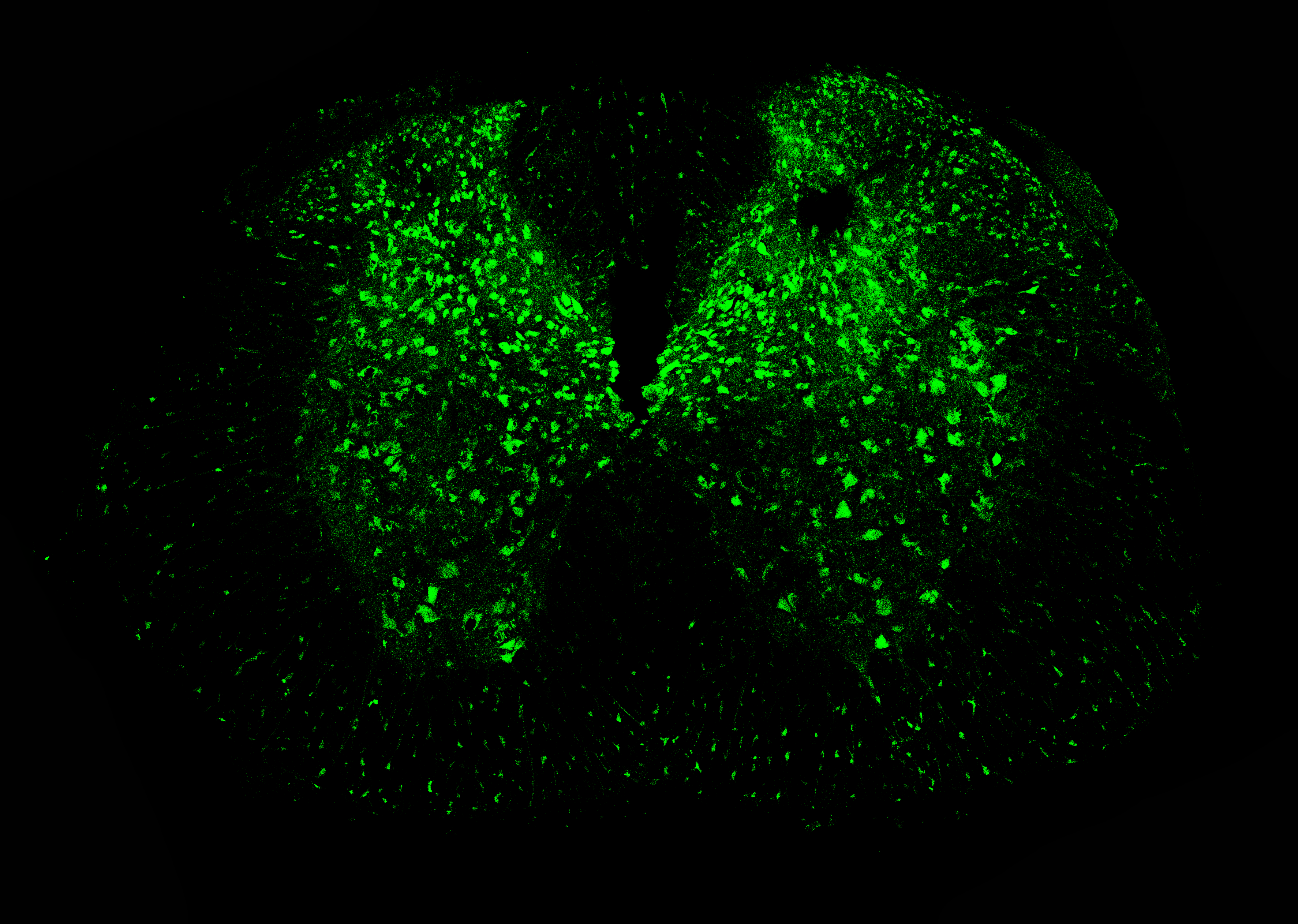

Supplement: Supplementary file 9 — Source Data Fig. 6 [file 44321_2024_37_MOESM9_ESM.zip › Fig 6/Fig6c/Healthy carrier ChAT_SMN/MD2.tif]

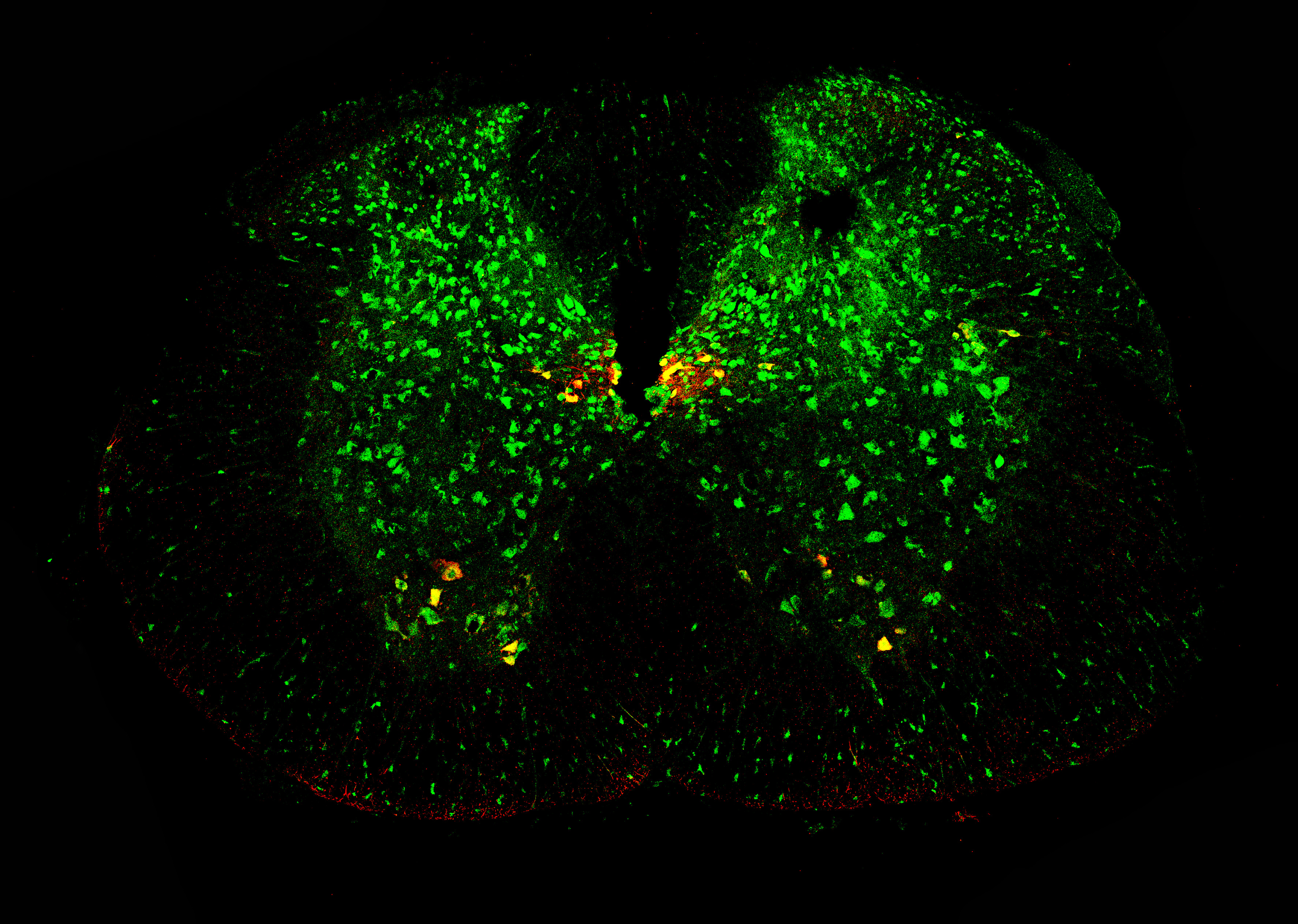

Supplement: Supplementary file 9 — Source Data Fig. 6 [file 44321_2024_37_MOESM9_ESM.zip › Fig 6/Fig6c/Healthy carrier ChAT_SMN/MD3.tif]

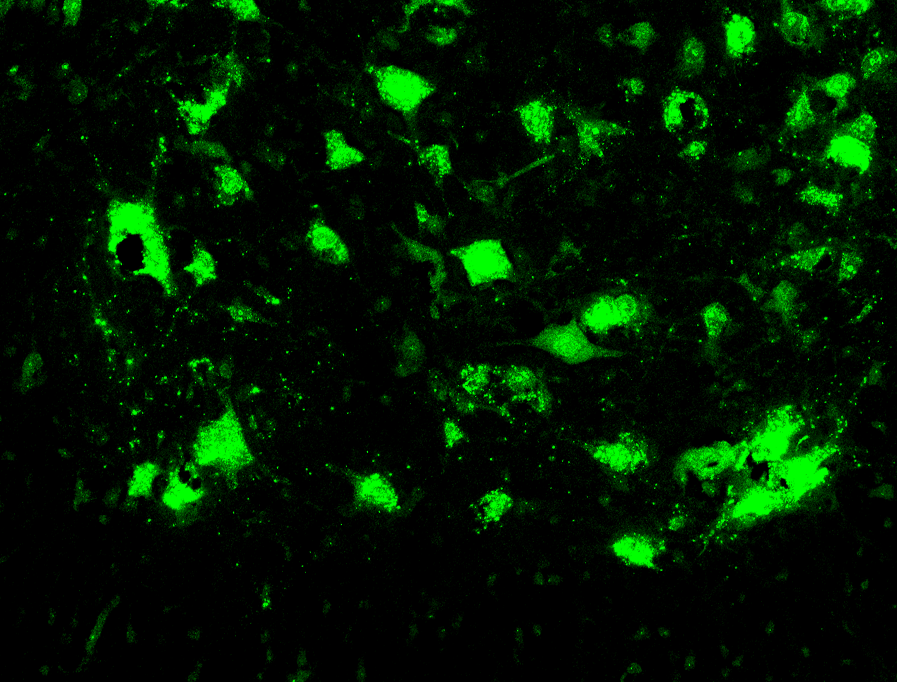

Supplement: Supplementary file 9 — Source Data Fig. 6 [file 44321_2024_37_MOESM9_ESM.zip › Fig 6/Fig6d/2nd Gen NeuN_SMN/Enlarged MD 2.tif]

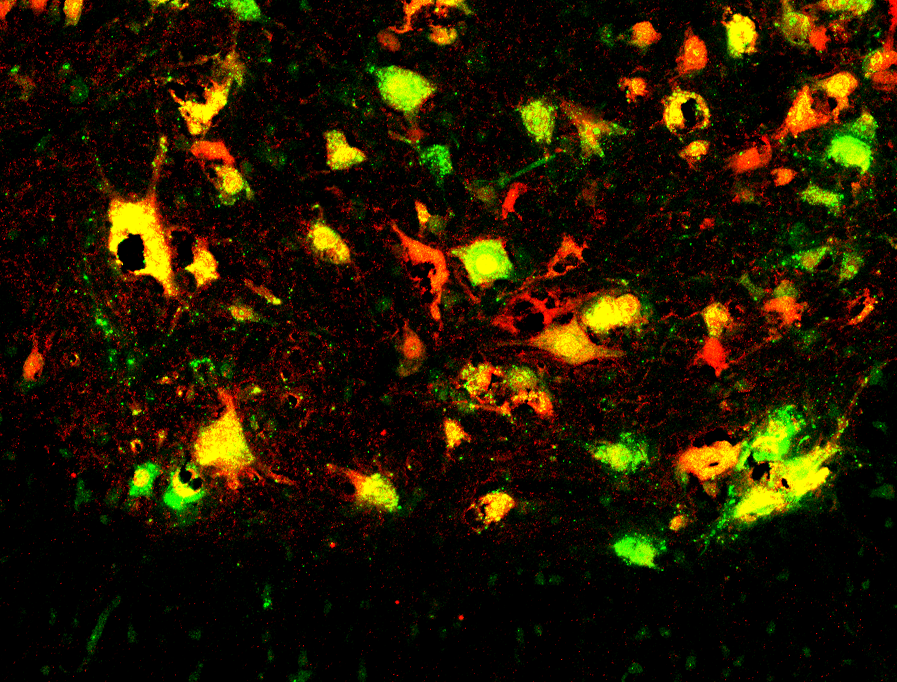

Supplement: Supplementary file 9 — Source Data Fig. 6 [file 44321_2024_37_MOESM9_ESM.zip › Fig 6/Fig6d/2nd Gen NeuN_SMN/Enlarged MD overlay.tif]

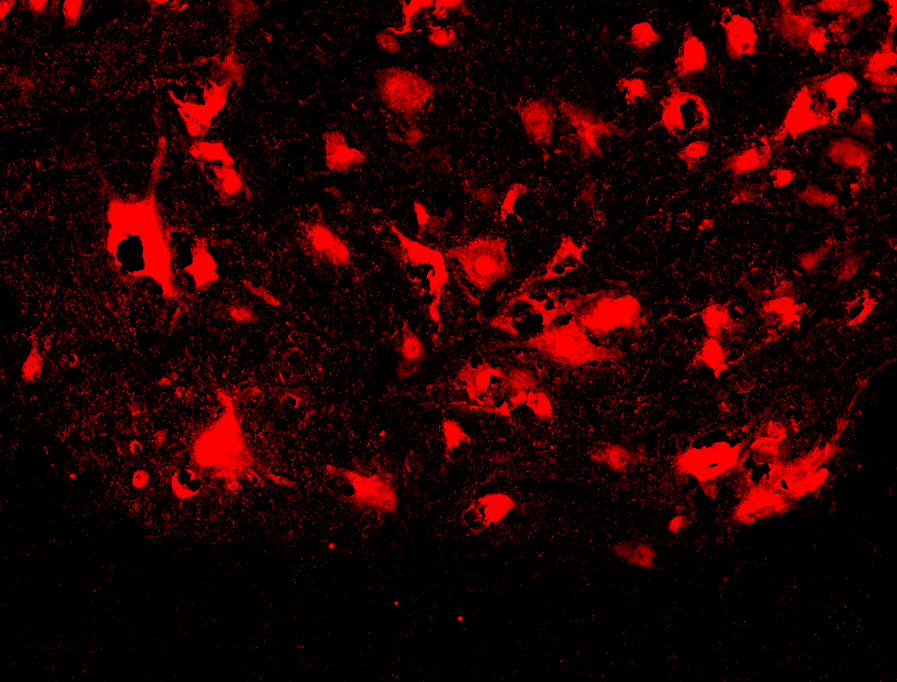

Supplement: Supplementary file 9 — Source Data Fig. 6 [file 44321_2024_37_MOESM9_ESM.zip › Fig 6/Fig6d/2nd Gen NeuN_SMN/Enlarged MD1.tif]

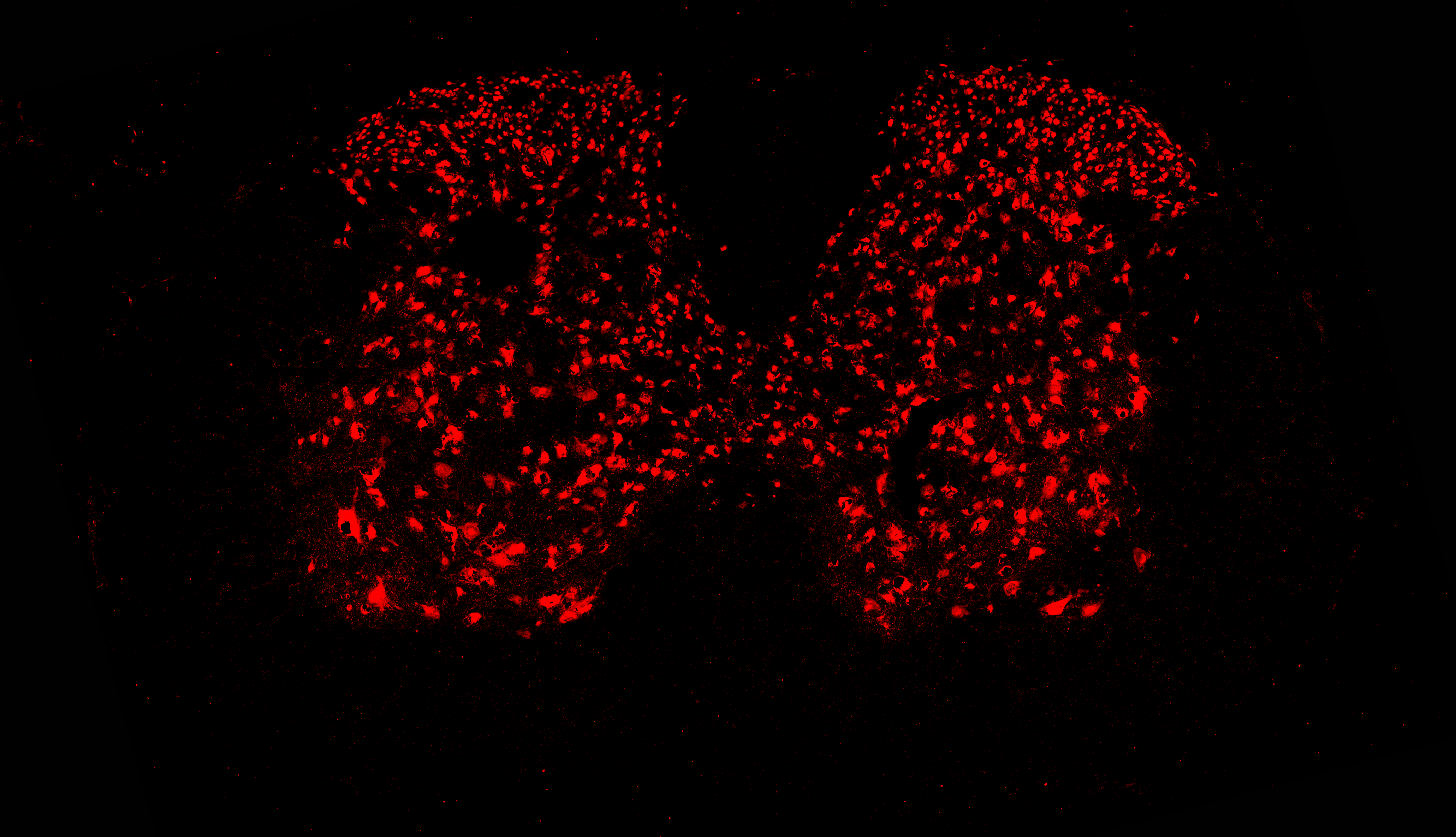

Supplement: Supplementary file 9 — Source Data Fig. 6 [file 44321_2024_37_MOESM9_ESM.zip › Fig 6/Fig6d/2nd Gen NeuN_SMN/MD 1.tif]

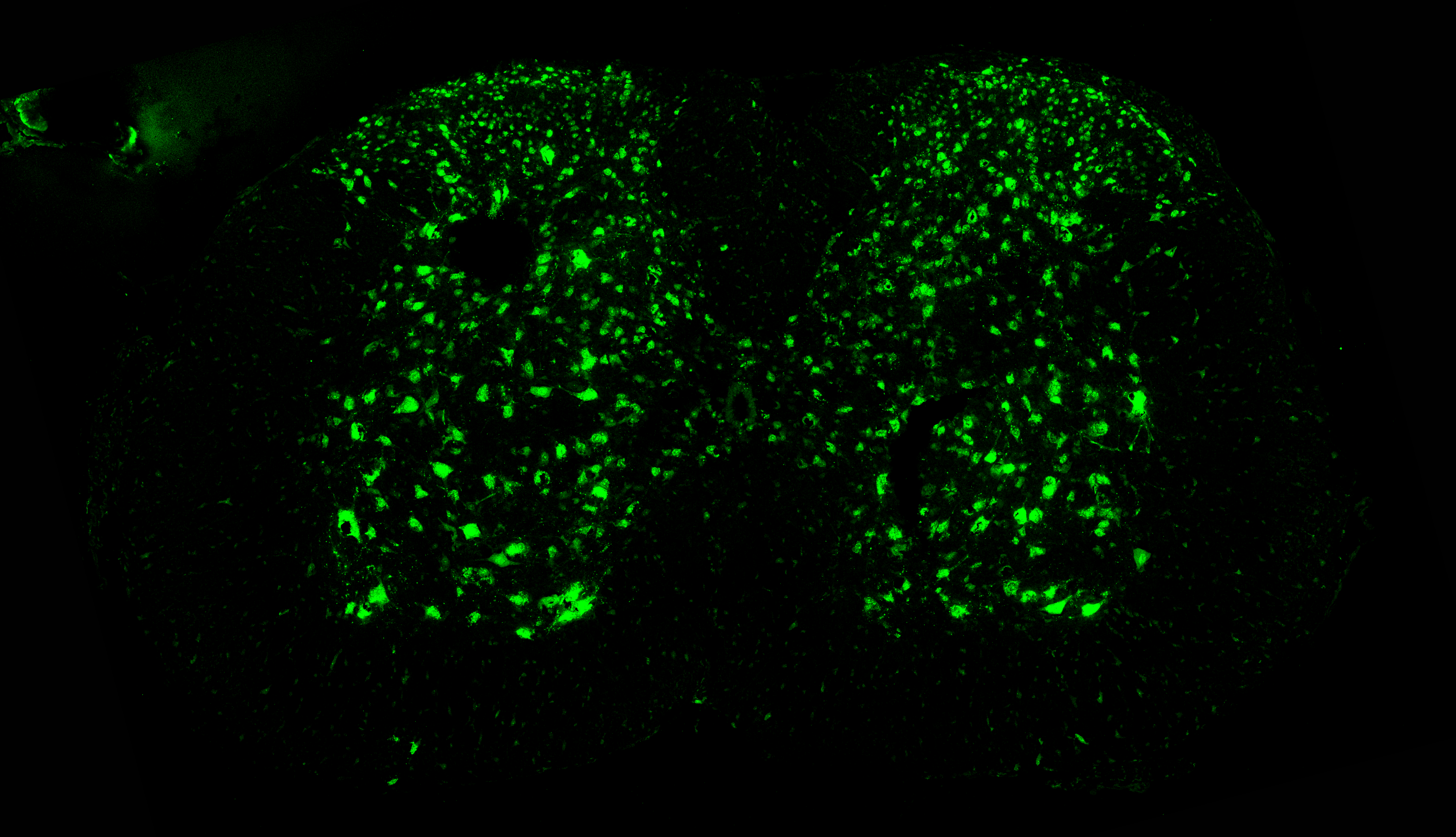

Supplement: Supplementary file 9 — Source Data Fig. 6 [file 44321_2024_37_MOESM9_ESM.zip › Fig 6/Fig6d/2nd Gen NeuN_SMN/MD 2.tif]

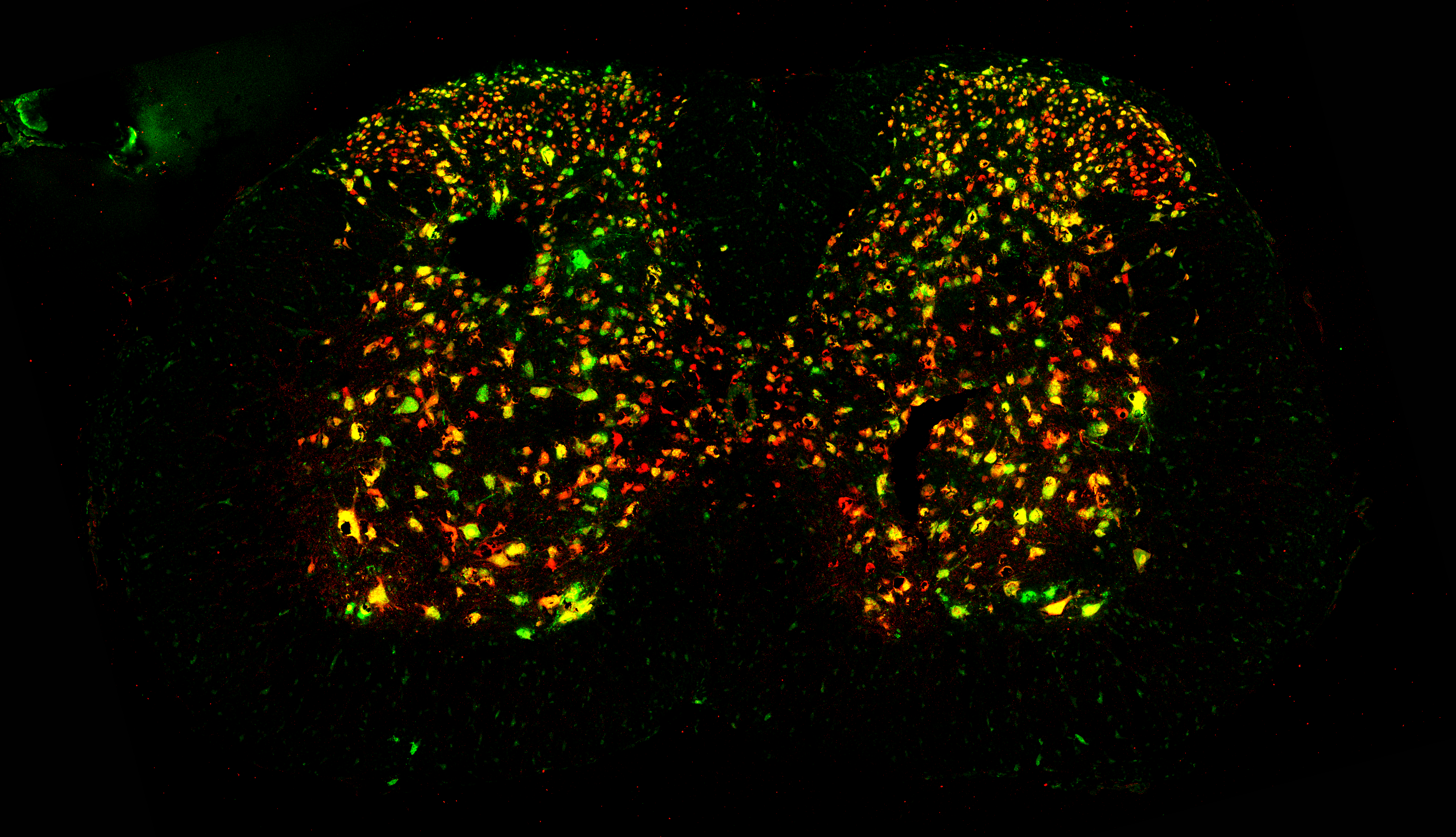

Supplement: Supplementary file 9 — Source Data Fig. 6 [file 44321_2024_37_MOESM9_ESM.zip › Fig 6/Fig6d/2nd Gen NeuN_SMN/MD overlay.tif]

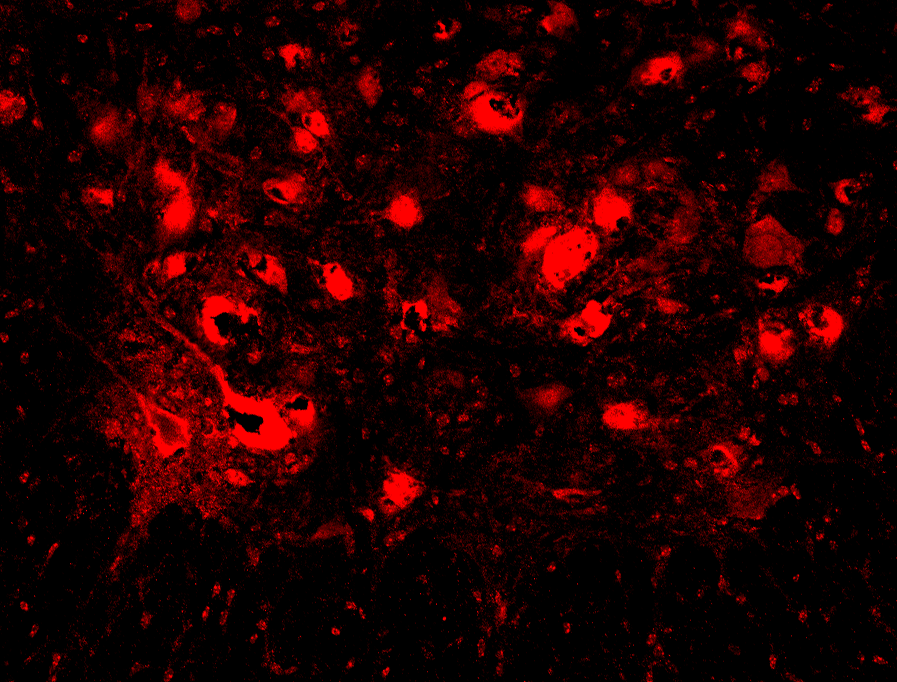

Supplement: Supplementary file 9 — Source Data Fig. 6 [file 44321_2024_37_MOESM9_ESM.zip › Fig 6/Fig6d/Benchmark NeuN_SMN/Enlarged MD1.tif]

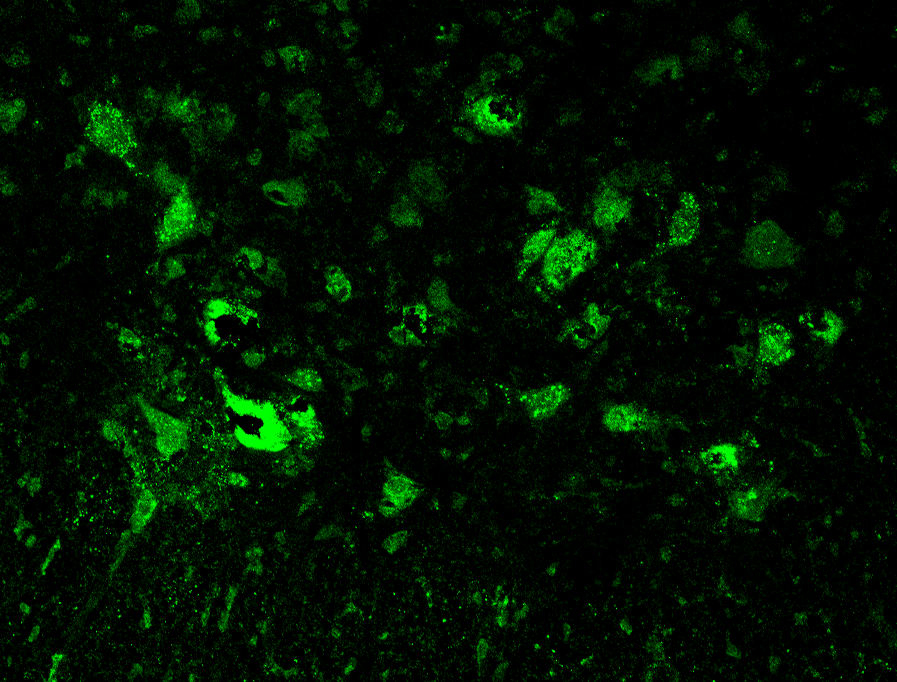

Supplement: Supplementary file 9 — Source Data Fig. 6 [file 44321_2024_37_MOESM9_ESM.zip › Fig 6/Fig6d/Benchmark NeuN_SMN/Enlarged MD2.tif]

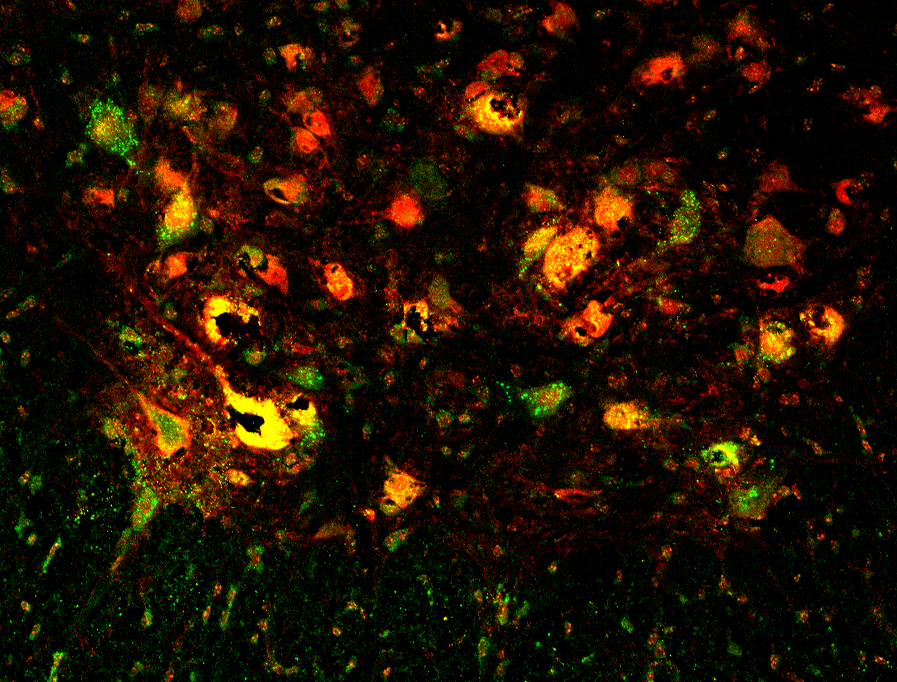

Supplement: Supplementary file 9 — Source Data Fig. 6 [file 44321_2024_37_MOESM9_ESM.zip › Fig 6/Fig6d/Benchmark NeuN_SMN/Enlarged MD3.tif]

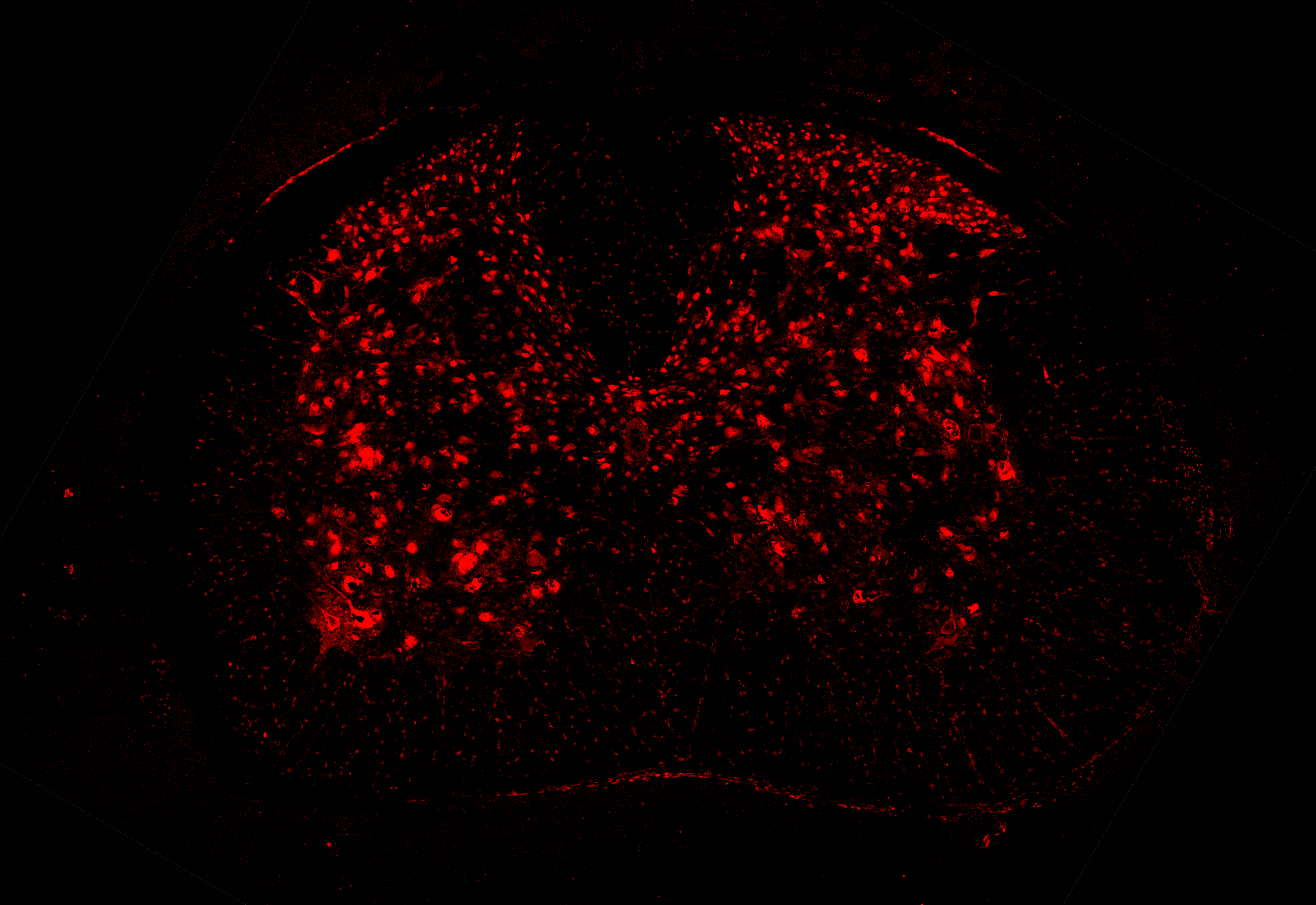

Supplement: Supplementary file 9 — Source Data Fig. 6 [file 44321_2024_37_MOESM9_ESM.zip › Fig 6/Fig6d/Benchmark NeuN_SMN/MD1.tif]

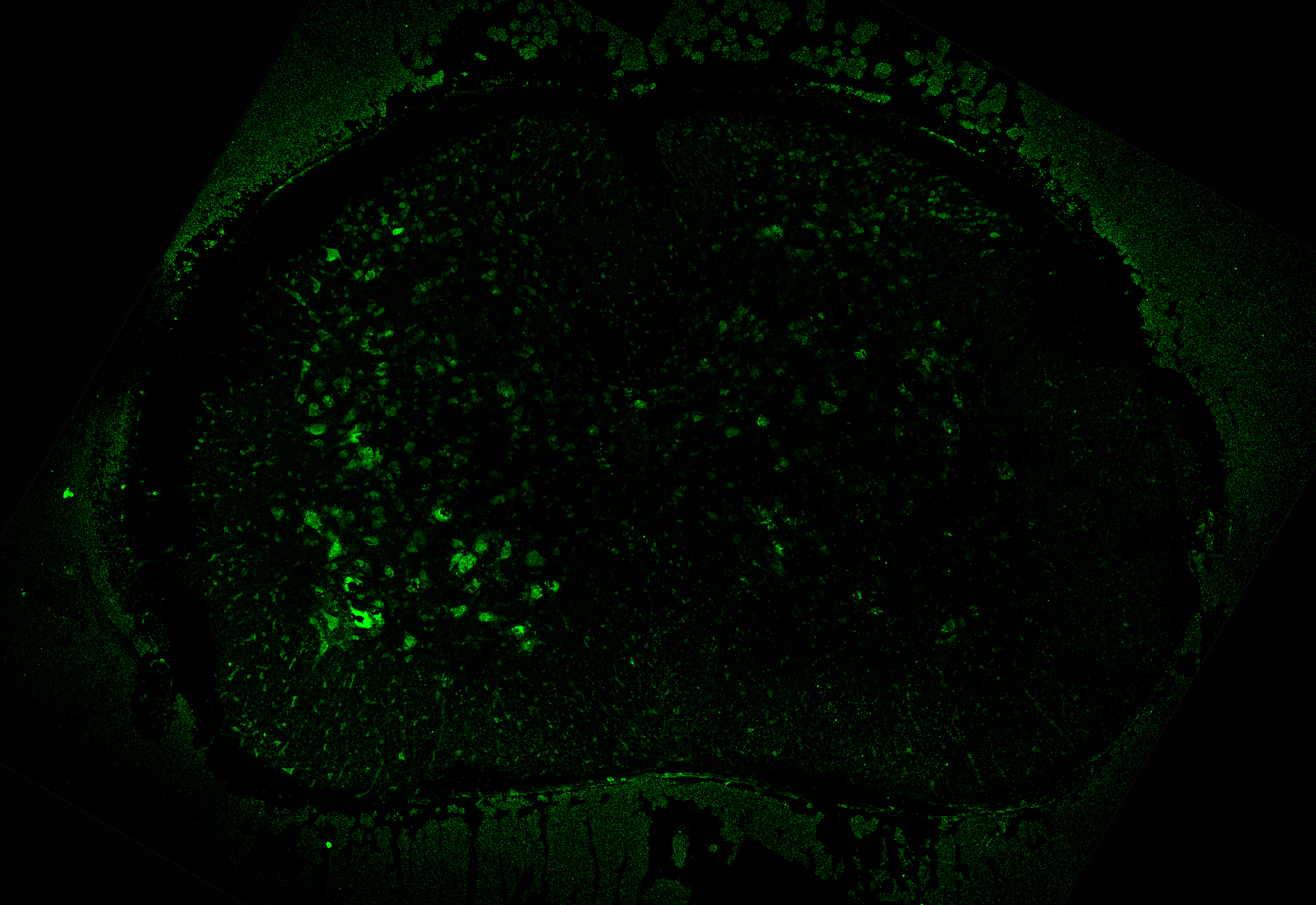

Supplement: Supplementary file 9 — Source Data Fig. 6 [file 44321_2024_37_MOESM9_ESM.zip › Fig 6/Fig6d/Benchmark NeuN_SMN/MD2.tif]

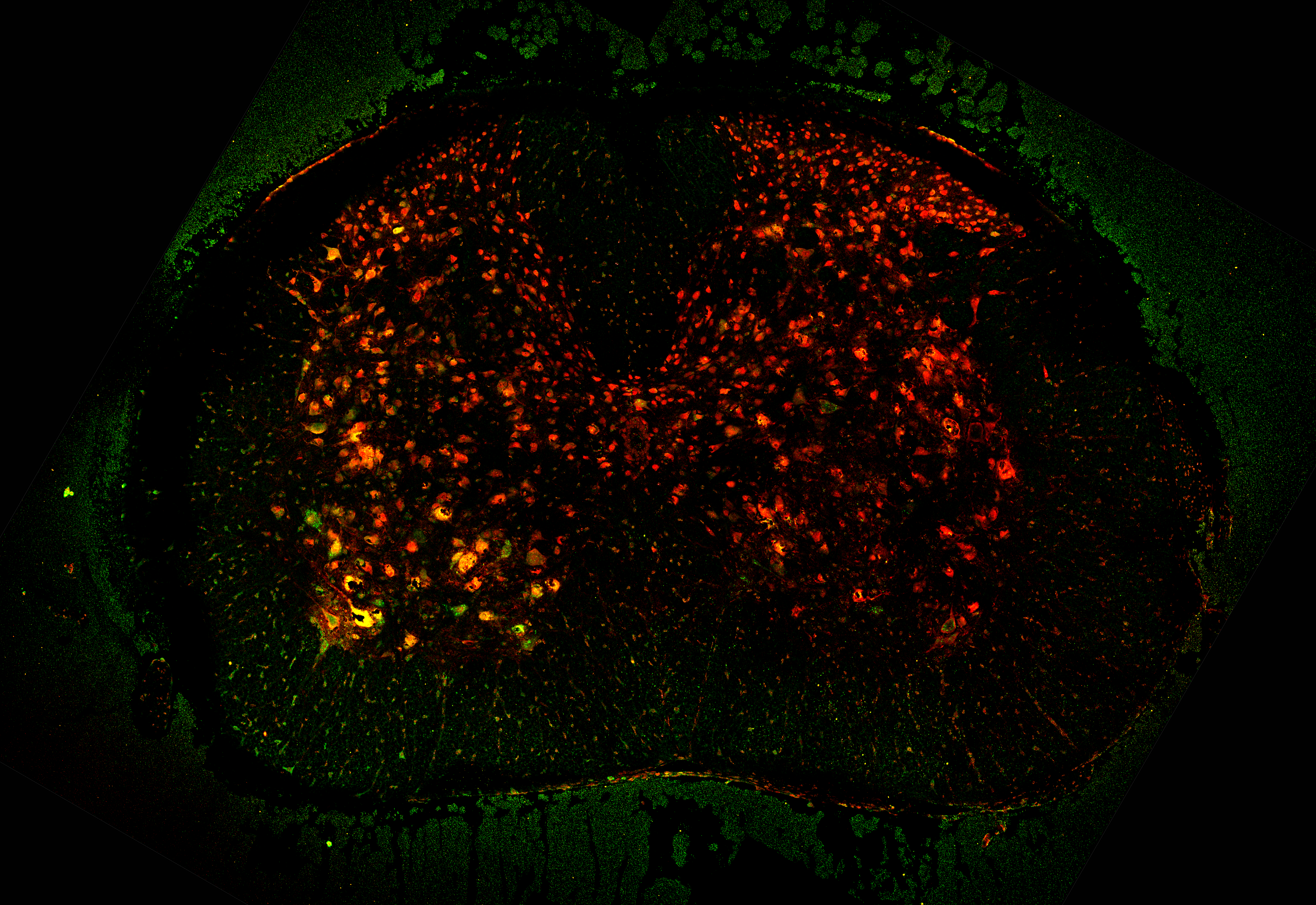

Supplement: Supplementary file 9 — Source Data Fig. 6 [file 44321_2024_37_MOESM9_ESM.zip › Fig 6/Fig6d/Benchmark NeuN_SMN/MD3.tif]

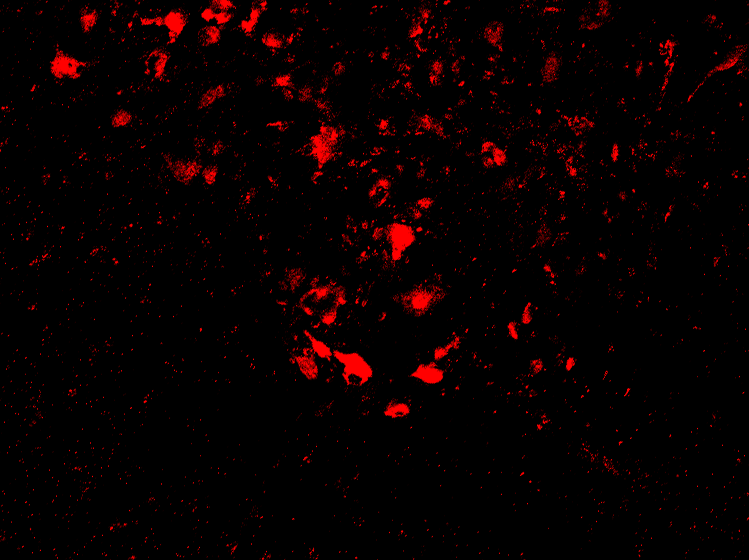

Supplement: Supplementary file 9 — Source Data Fig. 6 [file 44321_2024_37_MOESM9_ESM.zip › Fig 6/Fig6d/Healthy carrier NeuN_SMN/Enlarged MD1.tif]

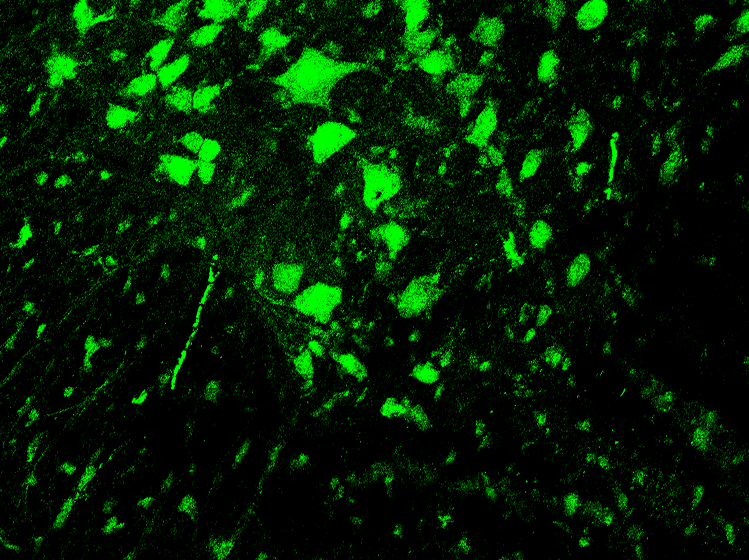

Supplement: Supplementary file 9 — Source Data Fig. 6 [file 44321_2024_37_MOESM9_ESM.zip › Fig 6/Fig6d/Healthy carrier NeuN_SMN/Enlarged MD2.tif]

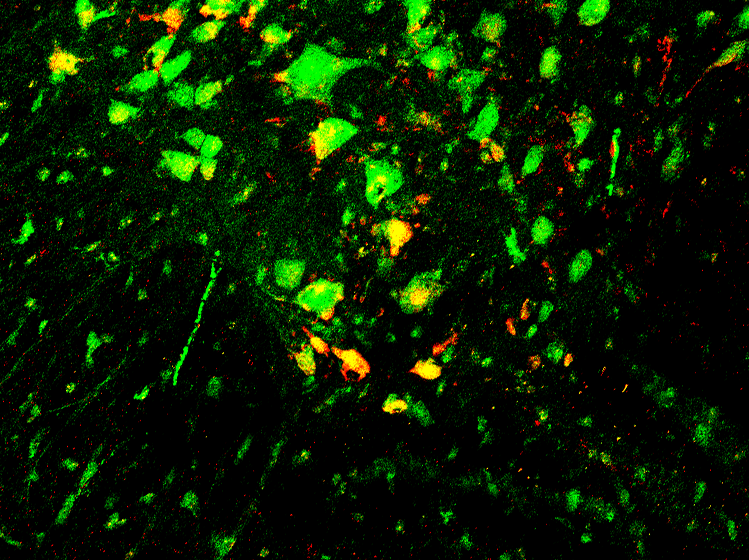

Supplement: Supplementary file 9 — Source Data Fig. 6 [file 44321_2024_37_MOESM9_ESM.zip › Fig 6/Fig6d/Healthy carrier NeuN_SMN/Enlarged MD3.tif]
