# Supplementary figures and images for: Improved gene therapy for spinal muscular atrophy in mice using codon-optimized hSMN1 transgene and hSMN1 gene-derived promotor (part 2 of 2)
Source: EMBO Mol Med. 2024 Feb 27;16(4):20. doi: 10.1038/s44321-024-00037-x (PMC11018631; doi:10.1038/s44321-024-00037-x)

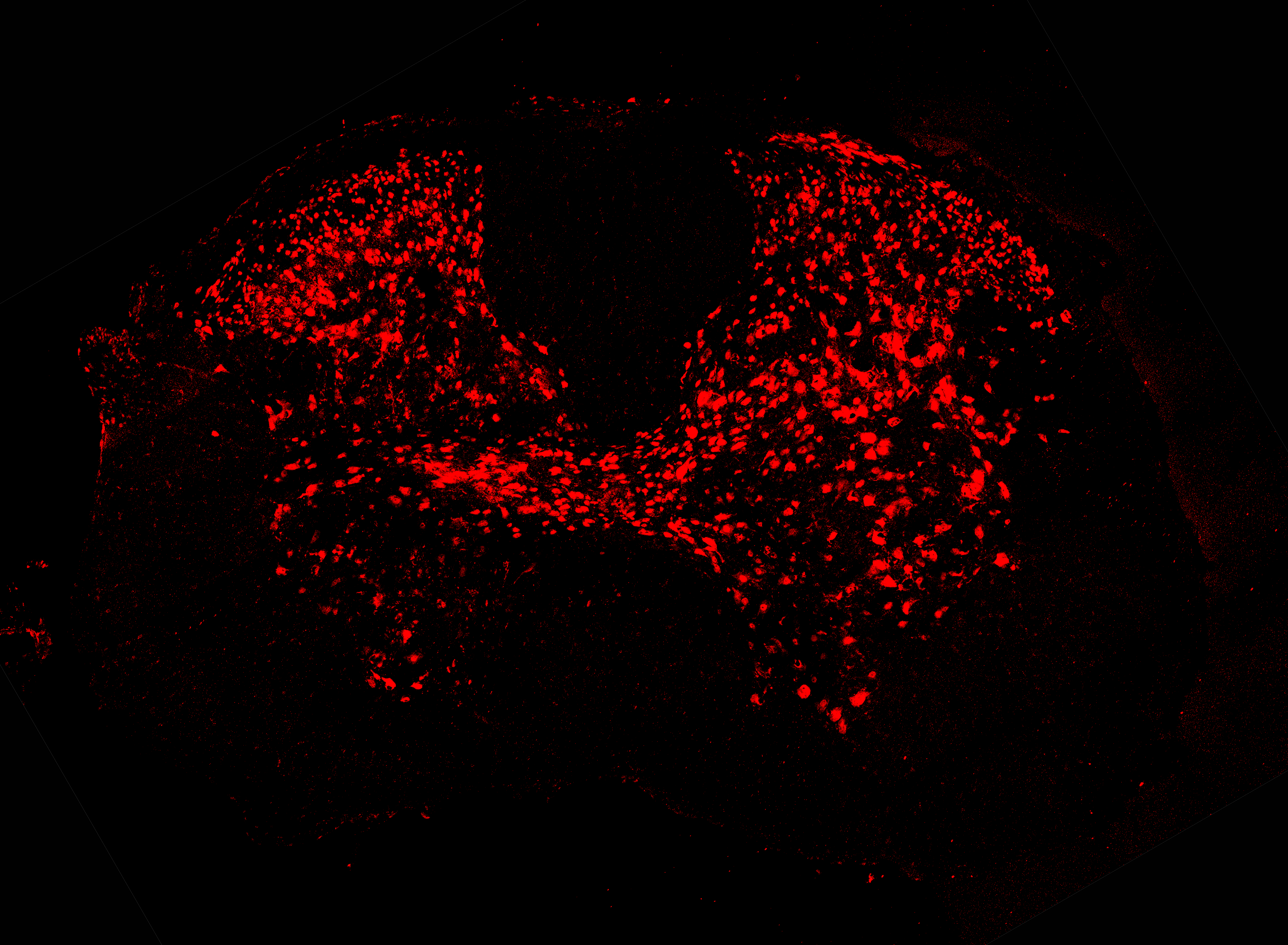

Supplement: Supplementary file 9 — Source Data Fig. 6 [file 44321_2024_37_MOESM9_ESM.zip › Fig 6/Fig6d/Healthy carrier NeuN_SMN/MD1.tif]

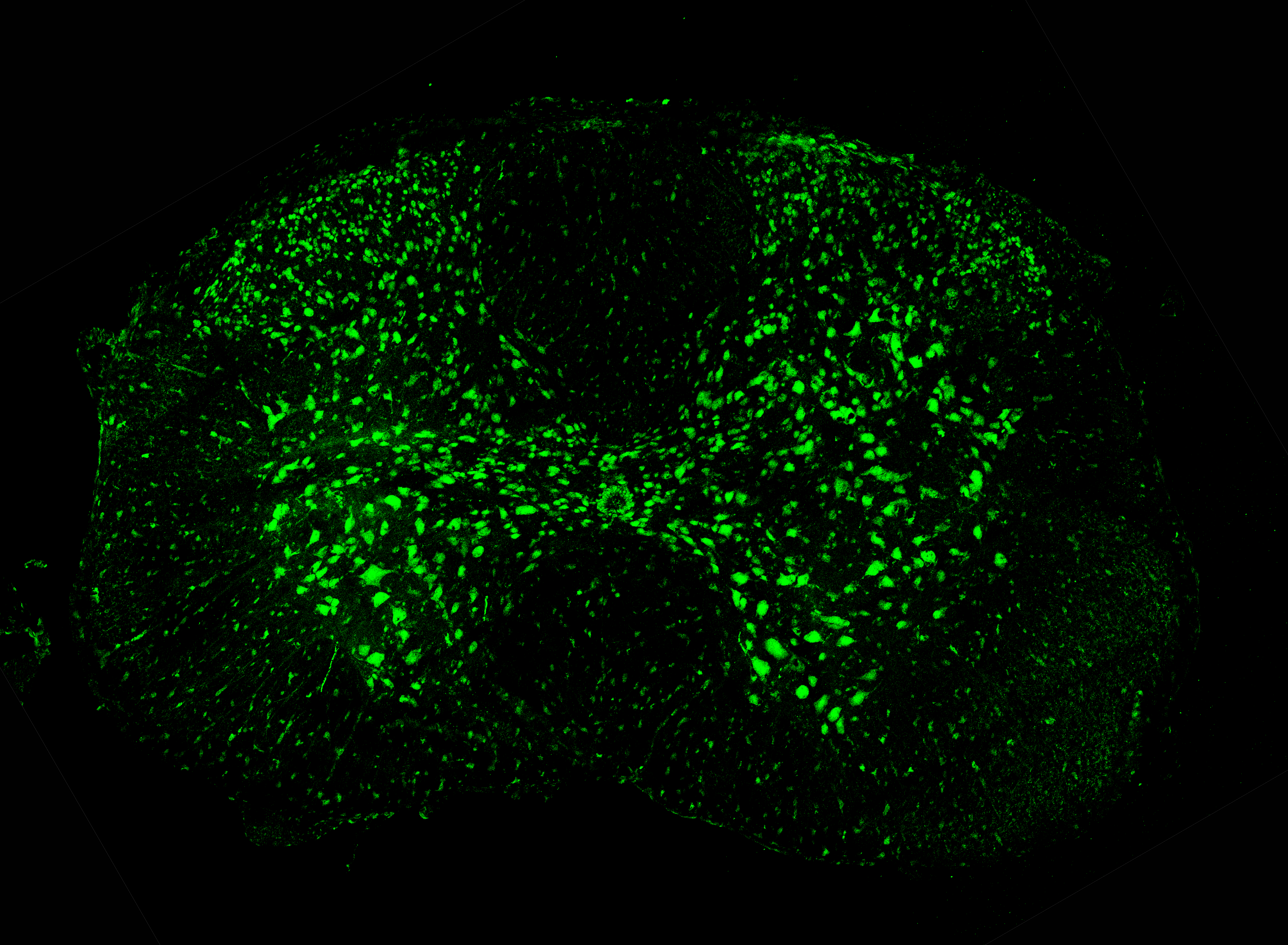

Supplement: Supplementary file 9 — Source Data Fig. 6 [file 44321_2024_37_MOESM9_ESM.zip › Fig 6/Fig6d/Healthy carrier NeuN_SMN/MD2.tif]

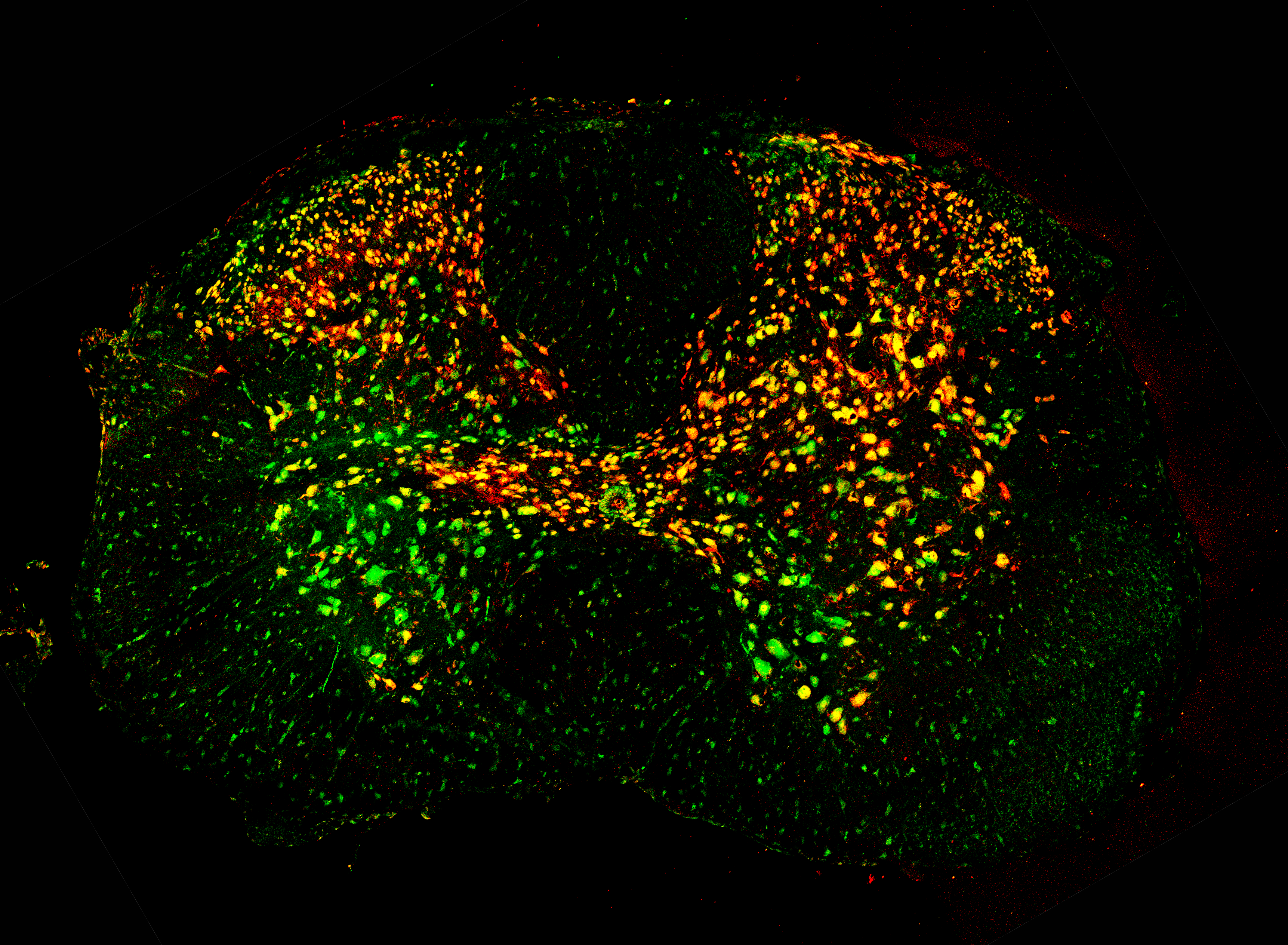

Supplement: Supplementary file 9 — Source Data Fig. 6 [file 44321_2024_37_MOESM9_ESM.zip › Fig 6/Fig6d/Healthy carrier NeuN_SMN/MD3.tif]
