# Supplementary material for: Improved gene therapy for spinal muscular atrophy in mice using codon-optimized hSMN1 transgene and hSMN1 gene-derived promotor
Source: EMBO Mol Med. 2024 Feb 27;16(4):20. doi: 10.1038/s44321-024-00037-x (PMC11018631; doi:10.1038/s44321-024-00037-x)
Supplement: Supplementary file 11 — Expanded View Figures [file 44321_2024_37_MOESM11_ESM.pdf]

Expanded View Figures

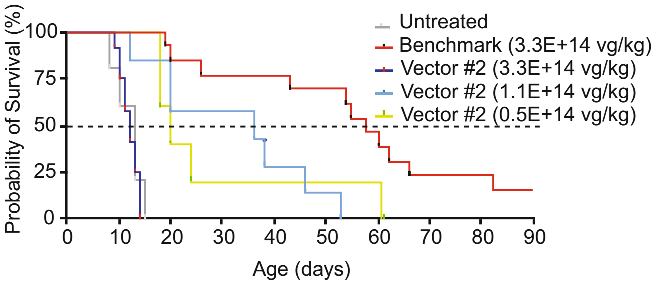

Figure EV1. Survival of SMA mice injected at P0 with Vector 2 at three different doses.

Non-injected SMA mice were used as a control.

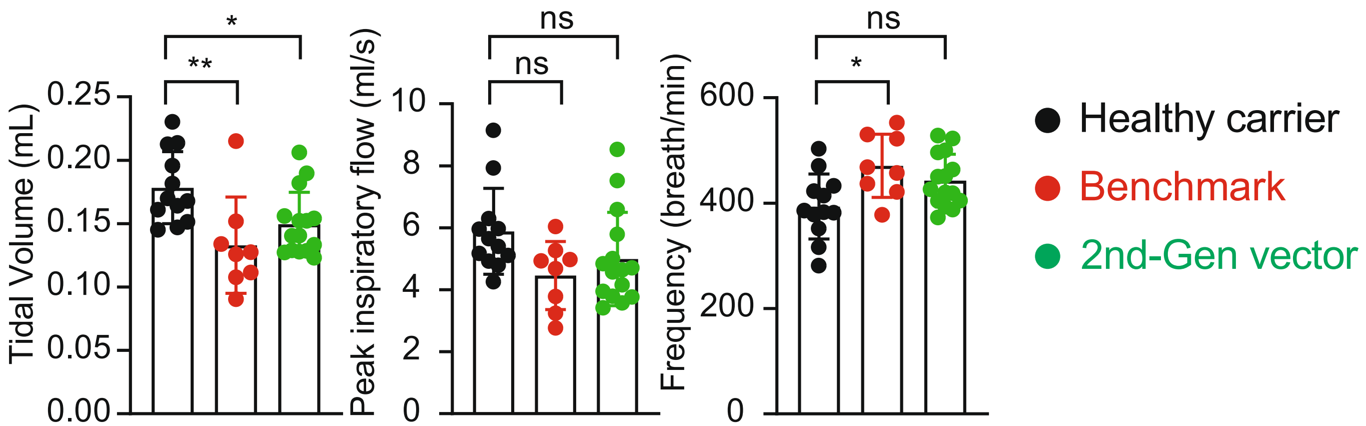

Figure EV2. Respiratory function assessment by plethysmography in SMA-treated mice and healthy carriers.

On Day 30, respiratory function was assessed by tidal volume, peak inspiratory flow, and respiratory rate. Bars represent the mean and error bars represent SD. One-way ANOVA, $*P < 0.05$; $**P < 0.01$; ns, not significant.

 © The Author(s)

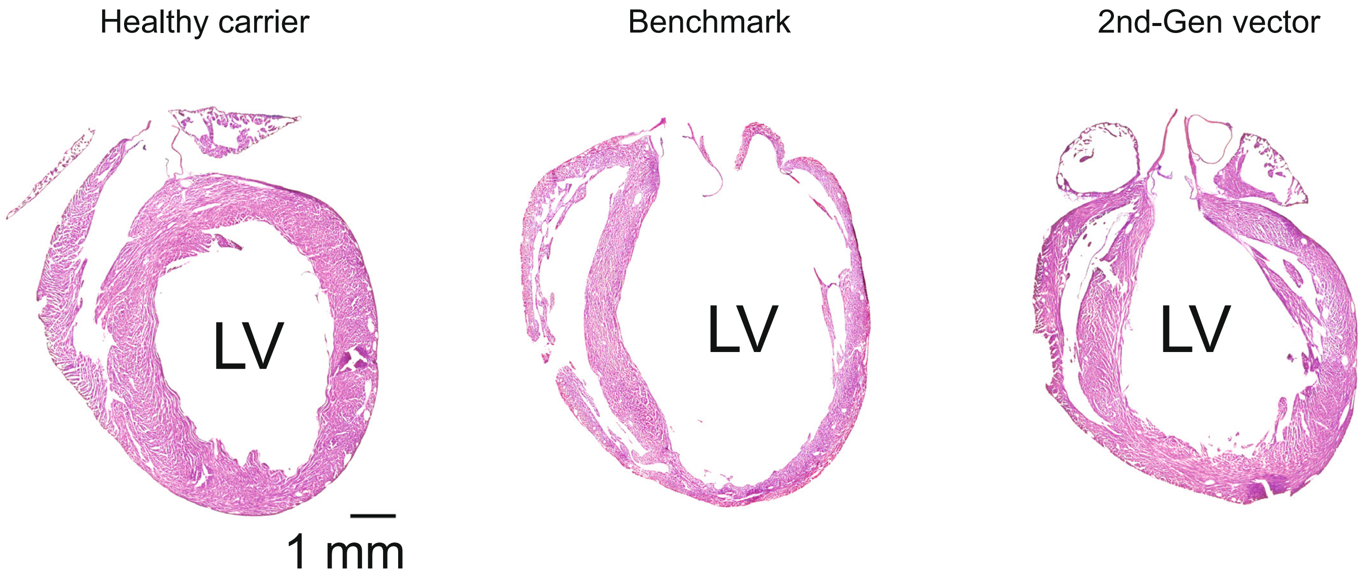

Figure EV3. Heart H&E staining on Day 90 of SMA injected at P0 with the benchmark and 2nd-generation vectors.

Healthy littermates were used as a control.

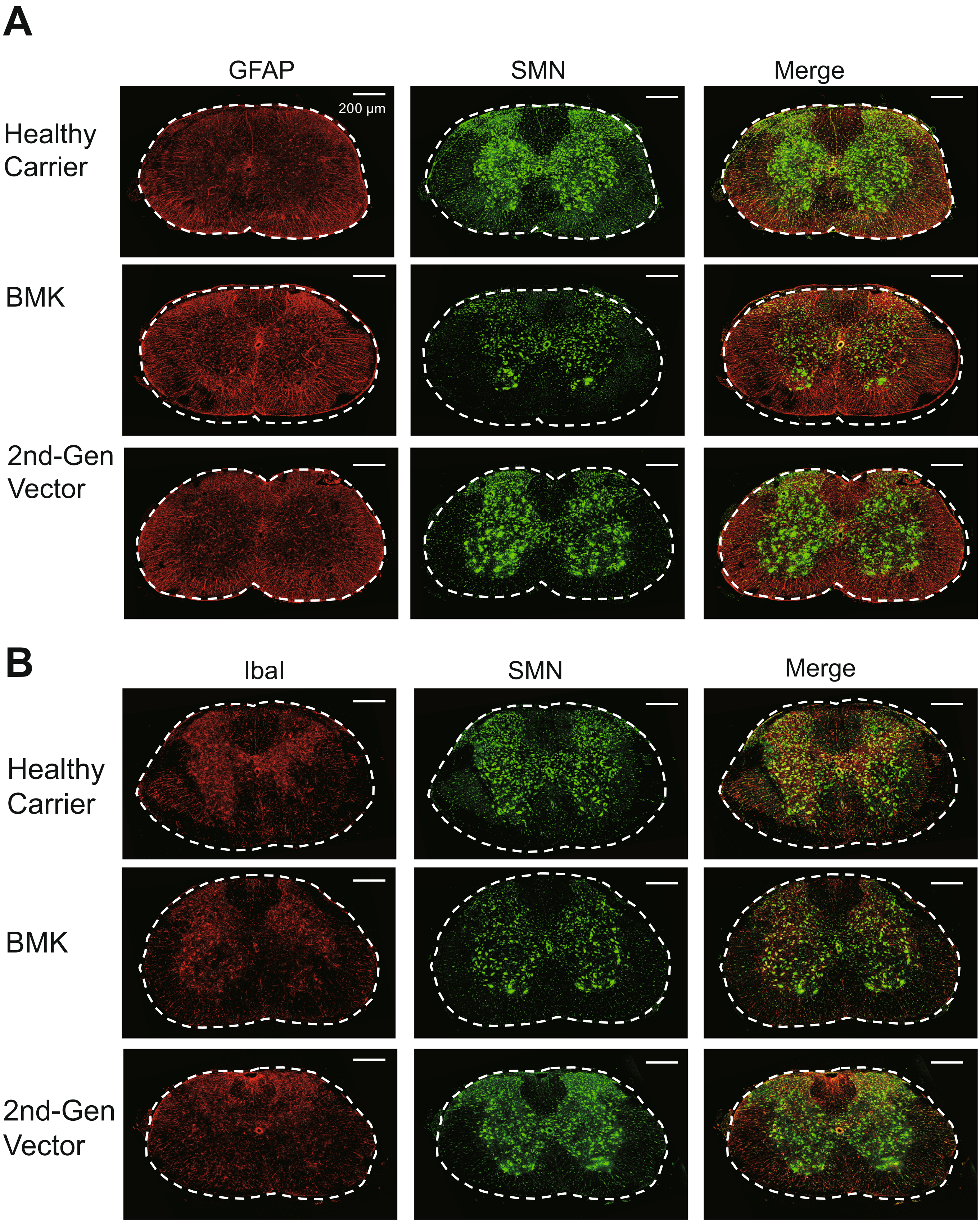
A
GFAP
SMN
Merge
200 μm
Healthy Carrier
BMK
2nd-Gen Vector
B
IbaI
SMN
Merge
Healthy Carrier
BMK
2nd-Gen Vector

 © The Author(s)

Figure EV4. Immunostaining of mouse lumbar spinal cord on Day 30.

(**A**) GFAP (red, an astrocyte marker), SMN (green), and merged (yellow). (**B**) Immunostaining of mouse lumbar spinal cord on Day 30. Iba1 (red, a microglial marker), SMN (green) and merged (yellow).

© The Author(s)
